# Multi-Omics Reveals the Effects of *Spirulina platensis* Powder Replacement of Fish Meal on Intestinal Metabolism and Stress in Zig-Zag Eel (*Mastacembelus armatus*)

**DOI:** 10.3390/antiox13070851

**Published:** 2024-07-15

**Authors:** Di Sun, Dongqiang Hou, Yushun Zheng, Wenzhou Xiang, Yingshi Huang, Hualian Wu, Jixing Zou

**Affiliations:** 1 College of Marine Sciences, South China Agricultural University, No. 483, Wushan Road, Wushan Street, Tianhe District, Guangzhou 510642, China; sundi815@163.com (D.S.); houdongqiang1026@163.com (D.H.); 20232150019@stu.scau.edu.cn (Y.Z.); 2 CAS Key Laboratory of Tropical Marine Bio-Resources and Ecology, Guangdong Key Laboratory of Marine Materia Medica, RNAM Center for Marine Microbiology, South China Sea Institute of Oceanology, Chinese Academy of Sciences, No. 164, West Xingang Road, Haizhu District, Guangzhou 510301, China; xwz@scsio.ac.cn; 3 Faculty of Mathematics, University of Waterloo, Waterloo, ON N2L 3G1, Canada; yingshi.huang@uwaterloo.ca

**Keywords:** algae utilization, transcriptomics, metabolomics, non-fishmeal protein sources, lipid metabolism

## Abstract

The booming aquaculture industry has created a strong demand for fishmeal and increased environmental pressures. Spirulina, as a potential alternative to fishmeal, has been shown to have growth-promoting and animal health-enhancing properties. In this study, 600 large spiny loaches, divided into five experimental groups, F0, F1, F2, F3, and F4, were reared for 10 weeks using *Spirulina platensis* powder (SPP) as a substitute for 0%, 5%, 10%, 15%, and 20% of fishmeal, respectively. The results of intestinal physiological indexes showed that superoxide dismutase was lower than F0 in all treatment groups, and the activity of F3 was significantly lower than F0 (*p* < 0.05). The activity of malondialdehyde was significantly higher than that of F0 in all groups except F3 (*p* < 0.05). The addition of SPP also led to a decrease in the activity of acid phosphatase in the intestine, which was significantly lower in all treatment groups compared to the F0 group (*p* < 0.05). The results of serum physiology showed that the activity of superoxide dismutase in serum gradually increased with the increase in the percentage of SPP addition, and the F3 group produced a significant difference from the F0 group (*p* < 0.05). The transcriptomics results showed that DEGs in the low percentage substitution group (<15%) were mostly enriched in metabolism-related pathways, such as bile secretion; DEGs in the high percentage substitution group (>15%) were mostly enriched in inflammation-related pathways, such as complement *p* and coagulation cascades. Metabolomics confirmed that nicotinate and nicotinamide metabolism and glycerophospholipid metabolism were the two pathways that were significantly enriched in the treatment groups of fishmeal replacement by SPP. The present study demonstrated that a low percentage (<15%) of fishmeal replacement by SPP in feed mobilized MA digestive metabolism, whereas a high percentage (>15%) of replacement induced intestinal stress. Considering the health and farm efficiency aspects, the proportion of SPP in feed formulation for MA should be less than 15%.

## 1. Introduction

Unlike land-based animal husbandry, the feeds used in aquaculture require a much higher protein content than livestock feeds [1]. Fishmeal is a feed ingredient used in large quantities (about 70% of commercial fishmeal) in the aquaculture industry, with a high protein content and rich in a variety of trace elements, as well as unknown growth factors [2]. The fishmeal used at present is mainly dependent on the harvesting of wild fish resources by the distant-water fishing industry; 25% of captured fish are used for fishmeal and fish oil production, causing a significant negative impact on marine environment sustainability [3,4,5]. The boom in aquaculture has also fueled the demand for fishmeal as a raw material for the industry, and as fishmeal production rises, it is bound to exacerbate the destruction of natural resources [6]. The cost of all this is that global FM production has been progressively reducing, from 5551 metric tons in 2010 to 4819 metric tons in 2022 [7]. Researchers have taken note of this problem, and a variety of potential protein ingredients for aquaculture feeds have been developed and investigated in response to the high protein demand of the aquaculture industry [8].

The existing protein sources used to replace fishmeal mainly include animal sources, plant sources, and microbial sources [9,10,11,12]. For various alternative sources, researchers have focused on the health status of the animals in addition to their growth performance after substitution. Inappropriate ingredients and inappropriate ratios can have a negative effect on animal health and growth, which can lead to a decrease in farm profitability and defeat the purpose of using alternative proteins in the first place [13]. Zhang et al. found that the use of soybean meal in place of 40% fishmeal in the diet exacerbated intestinal stress and resulted in reduced growth benefits [14]. The replacement of 30% fishmeal by black soldier fly meal increased the digestive enzyme activity but negatively affected the growth performance and intestinal health of the pearl gentian grouper (*Epinephelus fuscoguttatus* ♀ × *E. lanceolatus* ♂) [15].

Existing studies have demonstrated that marine microalgae proteins have better solubility and digestibility than other protein-based feeds and that, in addition to proteins, they contain high-value biomolecules that have a great potential to improve feed quality [16]. *Spirulina platensis* has been widely used around the world, and in the field of aquaculture, it not only stimulates the immune system of aquatic organisms but also serves as a source of protein in the feed and has a significant growth-promoting effect on aquatic organisms [17]. Antibiotics are recognized to be an important means of preventing and treating infectious diseases in fish, but they are prone to potential public food safety problems, so increasing the immune function of fish and improving the level of immunity is an important measure for preventing fish diseases in the aquaculture process [18]. Experiments have shown that rational feed additives have a positive effect on fish immunity [19]. Serum lysozyme and survival after *Aeromonas hydrophila* infection in gangetic mystus (*Mystus cavasius*) increased with increasing proportions of *S. platensis* added [20]. The same protective effect was found in experiments with Nile tilapia (*Oreochromis niloticus*) [21]. The above studies confirm that *S. platensis* has great potential for application in promoting fish growth and stimulating immunity.

The intestine is an important organ for digestion and absorption in animals, as well as a barrier for the organism to fight against external unfavorable factors, and in nutritional studies about aquatic animals, the intestine is one of the organs with the most obvious adaptive response to food in animals [22]. The assessment of intestinal health can reflect the rationality and potential impact of nutritional intake in animals. Wang et al. showed that replacing 50% fishmeal with cottonseed protein concentrate affects muscle mass in addition to intestinal stress [23]. The results of Galafat et al. confirmed that diets supplemented with low proportions of microalgae or hardened turbot (*Scophthalmus maximus*) larvae were suitable for improving digestive function as well as reducing lipid oxidation in muscle [24]. Omics technologies respond to the physiological state of an animal at different stages of life through a comprehensive or situational assessment of molecules, such as transcriptomics and metabolomics [25]. Transcriptomics has been used to help researchers unravel cell- to organ-specific signaling pathways based on the entire set of RNA transcripts expressed in a cell, defining the level of gene expression therein and the differentially expressed genes in control and experimental groups [26,27]. Unlike the transcriptome, the metabolome is concerned with the quantification and analysis of all metabolites in the animal organism, aiming to reveal the link between metabolites and physiological states [28]. Since the emergence of the two techniques, their combined analysis has been recognized as an effective method for exploring metabolic diversity among different samples and for revealing the underlying molecular mechanisms of related phenomena [29].

Zig-zag eel (*Mastacembelus armatus*, MA) is an emerging aquaculture species in southern China, with less research on its nutritional aspects, and its high protein requirement (about 50%) has been found during the aquaculture process, resulting in an extremely high proportion of fishmeal in the feed composition, which is not conducive to the cost control of the aquaculture process. The health condition of the animals under high-density aquaculture is far inferior to that of their wild counterparts [30]. Therefore, exploring the nutrient metabolism level of *M. armatus*, seeking suitable alternative sources of protein, and revealing the alterations in immune metabolism aspects of *M. armatus* under the modified substitution pattern are important for the development of the industry. In this experiment, *S. platensis* powder was used to replace 0% (F1), 5% (F2), 10% (F3), 15% (F4), and 20% (F5) of fishmeal in the feed formulation for a 10-week culture trial on MA. The aim of this experiment was to explore the potential effects of fishmeal replacement by different ratios of SPP on the MA’s intestine by biochemical indices, transcriptomics, and metabolomics. The results of the study will guide the application of SPP in MA breeding.

## 2. Materials and Methods

### 2.1. Statement of Ethics in Animal Experimentation

Animal experiments for this study were licensed by the Ethics Committee for Laboratory Animals of South China Agricultural University (Guangzhou, 2023g025), and all experimental work was performed according to ARRIVE guidelines.

### 2.2. Diet and Nutrition

The *S. platensis* powder (SPP) used in the experiment was provided by Lanqiang Biological Co., Ltd. in Foshan City, Guangdong Province, China, and was processed from *S. platensis*. The fish meal was provided by Guangzhou City Ke Mu Biotechnology Co., Ltd. (Guangzhou, China). The SPP was used to replace the fish meal in the feed formulation according to the proportions of 0% (F1), 5% (F2), 10% (F3), 15% (F4), and 20% (F5) in accordance with the experimental design. The feeds used in the experiment were in. It was made at the Guangdong Academy of Agricultural Sciences, China, and all operations were carried out at room temperature (air temperature 24–29 °C and humidity 80–90%). All ingredients were re-crushed before use so that all components could pass through a 178 μm aperture screen, and moisture, crude fat, crude protein, and crude ash contents were determined before use. According to the standard of 50% crude protein and 5.5% crude fat, the re-milled feed ingredients (except fish oil) were evenly mixed, and the final feed was in the form of powder with a uniform composition. The feed was tested again for moisture, crude fat, crude protein, and crude ash content to achieve the level of isonitrogenous (about 50% crude protein) and isolipidous (about 5.5% crude fat) content after the feed was made, and the specific feed formula and feed composition are shown in Supplementary Material S1 Table S1. The feed was stored in a sealed bag protected from light in the refrigerator at −20 °C.

The fish used in the experiment were purchased from Hai Fu Cang Aquaculture Company in Guangzhou City, Guangdong Province, China. The fish were brought back from the farm and kept in the constant temperature recirculating aquaculture system of the College of Marine Sciences, South China Agricultural University (SCAU). During the feeding period, the fish were fed with the control group feed to help them adapt to the culture environment in the laboratory as soon as possible. After two weeks of temporary rearing, 600 fish of uniform size, sufficiently healthy and vigorous, and without deformities were selected and divided into 5 groups. A total of five groups were set up in the experiment, including one control group (F0) and four treatment groups (F1, F2, F3, and F4), and three fish tanks were set up as parallels for each group, with forty fish placed in each tank. During the rearing period, it was ensured that the water was circulated for 24 h, the oxygen content of the water body was >5 mg/L, and the light was set at 12:12 h. The rearing trial lasted for ten weeks.

At the beginning of the experiment, the feeds were fed according to the pre-grouping. A fixed mass of feed was weighed for each feeding, and after adding fish oil at the ratio designed for the feed formulation (Supplementary Material S1 Table S1), a mass-to-volume ratio of 1:1.5 (g/mL) of distilled water was added. The mixture was stirred well in a fixed direction using a stainless steel stirring bar until the mixture was lumpy, then the mixture was placed in a feeding dish and weighed. One hour after feeding, the remaining feed was collected and weighed again, and feed consumption was recorded (see Table S2 in the Supplementary Material S1 for feed consumption and growth indicators). The fish were fed twice a day at 9:00 and 16:00 during the 10-week experimental period.

### 2.3. Sample Collection

At the end of the experiment, after 24 h of fasting, all the fish were weighed and measured for body length. After excluding small and large fish based on their average weight and length per tank, six uniformly healthy fish were randomly selected from each tank by a specific experimenter without being informed of the grouping. The selected fish were sampled and anesthetized using MS-222. Blood was collected through the tail vein, and the collected blood was allowed to stand at room temperature for 6 h, then centrifuged (3000 rpm, 15 min) to obtain serum for subsequent serum biochemical analysis. After blood collection, the fish were dissected, and the intestinal tissues were collected, quick-frozen in liquid nitrogen, and then transferred to a refrigerator at −80 °C for subsequent intestinal biochemical analyses, transcriptomics, and metabolomics tests.

### 2.4. Biochemical Indices Assay

Serum biochemical indexes such as superoxide dismutase (SOD), total antioxidant capacity (T-AOC), malondialdehyde (MDA), catalase (CAT), total cholesterol (T-CHO), acid phosphatase (ACP), and alkaline phosphatase (AKP) were determined by using kits produced by Nanjing Jiancheng Bioengineering Institute (Nanjing, China). Serum samples were diluted by adding 0.9% saline according to the procedure for product use. The biochemical indices SOD, T-AOC, MDA, CAT, ACP, and AKP were measured using kits produced by the Nanjing Jiancheng Bioengineering Institute. Ice-cold 0.9% saline solution (*m*/*v* = 1 g: 9 mL) was added to the intestinal samples, the samples were ground into homogenate at 4 °C, and the supernatant was collected for the evaluation of biochemical indicators. In order to accurately calculate the biochemical indexes of intestinal tissues, the protein concentration of all samples was determined using a protein concentration kit produced by Nanjing Jiancheng Bioengineering Institute (Nanjing, China) [31].

### 2.5. Transcriptomics Testing

RNA library sequencing was performed on an Illumina HiseqTM 2500/4000 by Genetic Dino Biotech (Guangzhou, China). RNA was extracted from the samples using the Trizol reagent method, and the quality of the total RNA extracted in the study was assessed using RNase-free agarose gel electrophoresis and an Agilent 2100 Bioanalyzer. After extraction of enriched eukaryotic mRNA using Oligo (dT) beads, the enriched RNA was disaggregated and reverse transcribed into cDNA using the NEBNext Ultra RNA Library Preparation Kit for Illumina. Prior to sequencing, the resulting cDNA libraries undergo end repair, base addition, ligation to Illumina sequencing adapters, and PCR amplification. Bioinformatics methods included filtering of high-quality pure reads using Fastp, comparison with the ribosomal RNA (rRNA) database to exclude reads mapping to rRNA, and comparison with a reference genome using HISAT2, which was used for the experiment (*Mastacembelus armatus* GCA_900324485.3) [32,33,34]. Read assembly and gene abundance calculations were performed using StringTie [35,36,37]. Principal component analysis and correlation analysis were used to investigate sample relationships. deseq2 and edgeR were used to identify differentially expressed genes (DEGs) (the parameter of false discovery rate (FDR) below 0.05 and absolute fold change ≥ 2 was considered differentially expressed genes/transcripts) [38,39]. GO, KEGG, disease ontology, and reactome enrichment studies were then performed to identify biological functions associated with DEGs (taking FDR ≤ 0.05 as a threshold) [40,41,42,43,44]. Please refer to Supplementary Material S1 for specific experimental steps.

### 2.6. Metabolomics Assays

The chemicals used were from Sigma Aldrich, Merck, and Fisher and included NH4AC, acetonitrile, NH_4_OH, and methanol. Intestinal tissues were frozen in liquid nitrogen, homogenized, and metabolites were extracted with methanol/acetonitrile. LC-MS/MS analyses were performed using ultra-high-performance liquid chromatography with RPLC and HILIC separation. Raw MS data were converted to MzXML files for XCMS analysis, and CAMERA was available to aid in compound identification against internal databases. The KNN method resolves missing data and extreme values, and peak areas were subsequently normalized to ensure sample metabolite consistency. Metabolite assays use both positive and negative ion modes for enhanced coverage. Quality control samples were subjected to PCA using R to ensure reliability. Cluster analyses included heat map analysis with clusters to visualize similarities in metabolic composition and correlation analysis of replicate samples using the R package. Multivariate statistical analyses included PCA for initial visualization, PLS-DA for feature differentiation using the R (4.2.1) software package ropls, and OPLS-DA with orthogonal signal correction validated by cross-validation and permutation tests. Differential metabolite analyses relied on VIP scoring of the (O)PLS model and *t*-test screening for the detection of between-group differences (*p* value of *t*-test < 0.05, and VIP ≥ 1 was considered differential metabolites between two groups). KEGG analysis was used to identify biological functions associated with metabolites (taking FDR ≤ 0.05 as a threshold). See Supplementary Material S1 for detailed experimental procedures.

### 2.7. RT-qPCR to Detect the Expression Pattern of Related Genes

The total RNA was extracted using the Trizol method. cDNA synthesis was performed using the Prime Script™ RT kit and the gDNA Eraser (Perfect Real Time) (TAKARA, Dalian, China) kit. Finally, gene expression was determined by the SYBR method using a standard commercial kit (SYBR Green Premix Pro Taq HS qPCR Kit, Accurate Biology, Changsha, China). The equipment used for the experiments was the BIO-RAD Real-Time PCR Detection System (BIO-RAD, Hercules, CA, USA). Table 1 lists the qRT-PCR primers used in this study. All samples were normalized to *β*-actin as a control. The 2^−ΔΔCt^ method was used to calculate the relative expression of target genes [18].

### 2.8. Statistical Analysis

All experiments had at least three replicates. All experimental data were counted and calculated by Excel 2019 and expressed as mean ± standard deviation (mean ± SD). Statistical analyses included analysis of variance (ANOVA) and the Sidak test by IBM SPSS Statistics 25. The qRT-PCR data were tested for homogeneity of data variance using Levene’s test before statistical analysis, and Shapiro–Wilk’s test was used to confirm whether the data were normally distributed. The data analysis methods used in the transcriptomics and metabolomics section are detailed in 2.6 Transcriptomics testing and 2.7 Transcriptomics testing. The data of *p* < 0.05 were considered statistically significant.

## 3. Results

### 3.1. Effects of Different Ratios of SPP on the Antioxidant Capacity of MA’s Intestinal and Serum

**Figure 1:**
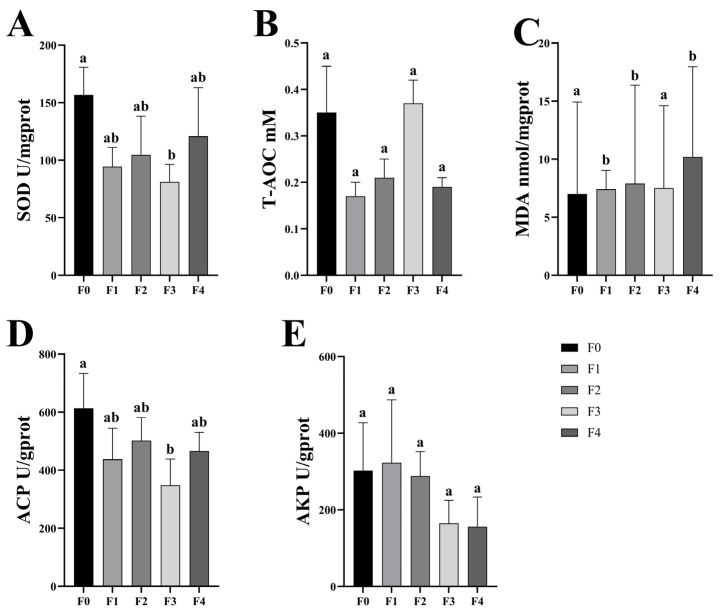
Antioxidant capacity measurement, including the superoxide dismutase (SOD), total antioxidant capacity (T-AOC), malondialdehyde (MDA), acid phosphatase (ACP), and alkaline phosphatase (AKP) detection in MA intestinal (**A**–**E**). Note: Values are means ± SD; *n* = 3. Different superscript letters indicate significant differences, and the significance level for all statistical tests is *p* < 0.05, below.

The antioxidant capacity of different ratios of SPP in the intestine of MA is shown in Figure 1. The biochemical indices included SOD, T-AOC, MDA, ACP, and AKP. The activity of SOD was lower than that of F0 in all the treatment groups, and it is noteworthy that the activity of F3 was significantly lower than that of F0 (*p* < 0.05). The activity of MDA was significantly higher (*p* < 0.05) than that of F0 in all groups except F3. The addition of SPP also led to a decrease in the activity of ACP in the intestine, which was significantly lower (*p* < 0.05) in all treatment groups than that of F0. There was a decrease in T-AOC and AKP activity in the treatment groups compared to the F0 group, but this difference was not statistically significant. This suggests that the addition of SPP leads to oxidative stress in the intestine.

**Figure 2:**
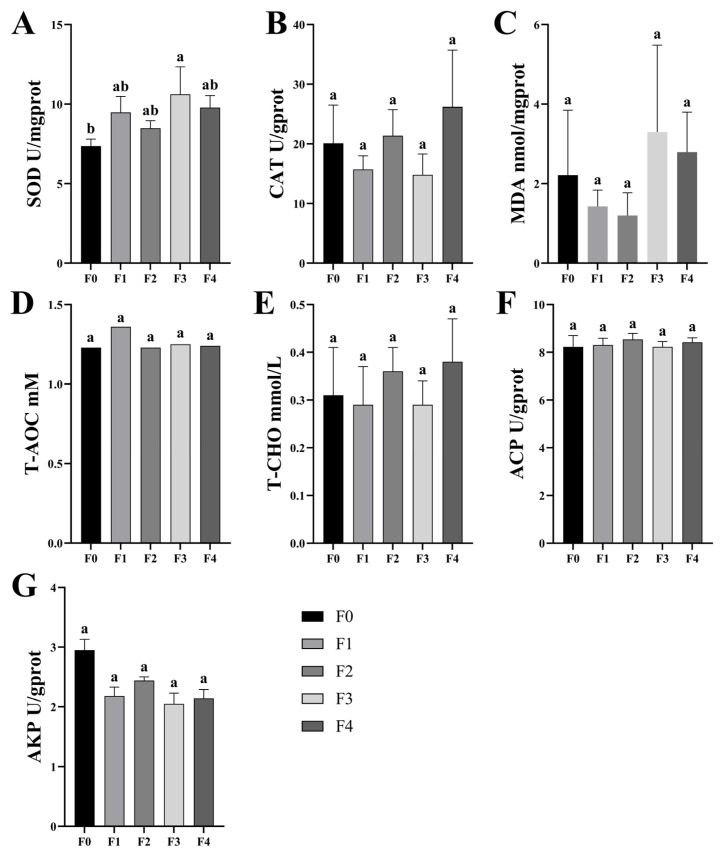
Antioxidant capacity measurement, including the superoxide dismutase (SOD), catalase (CAT), malondialdehyde (MDA), total cholesterol (T-CHO), total antioxidant capacity (T-AOC), acid phosphatase (ACP), and alkaline phosphatase (AKP) detection in MA serum (**A**–**G**). Different superscript letters indicate significant differences, and the significance level for all statistical tests is *p* < 0.05, below.

The effect of different ratios of SPP addition on the antioxidant power of MA serum is shown in Figure 2. The biochemical indices examined in the serum included SOD, T-AOC, MDA, CAT, cholesterol, ACP, and AKP. The activity of serum SOD increased gradually with the increase in the percentage of SPP addition, which produced significant differences between the F3 group and the F0 group (*p* < 0.05). The differences between the treatment groups and the F0 group for the remaining indexes were not statistically significant. This part of the experiment showed that the effect of SPP addition on the serum antioxidant capacity of MA was mainly to enhance the activity of SOD.

### 3.2. Transcriptome Analysis of Effects of Different Ratios of SPP on MA’s Intestine

Three replicate samples were set up for transcriptomics analysis in each of the five groups; a total of 104.01 G of raw data were obtained, and a total of 101.86 G of pure data were obtained after filtering, with an average of 5.66 G of pure data obtained for each sample. The average percentages of Q20 and Q30 scores for each sample were 97.57% and 95.5%, respectively, and the number of pure reads obtained ranged from 36,307,444 to 47,359,346 (Table 2). These data indicate that the analysis of the present transcriptome has high confidence and that the quality of the resulting sequences is adequate for subsequent analysis.

**Figure 3:**
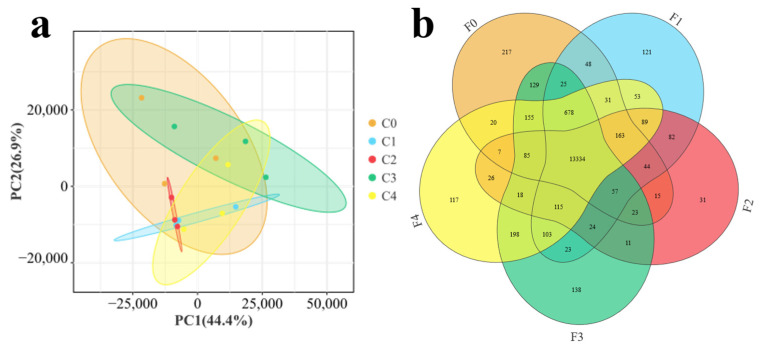
Correlations between samples of the intestinal transcriptome of different treatment groups in MA, including (**a**) between-sample PCoA analysis (ovals represent 95% confidence intervals and the same color is used for samples in the same group) and (**b**) Veen analysis between control and treatment groups (groups are filled in with different colors, with overlaps representing (2 or n) co-expressed genes between groups; numbers indicate the specific number of co-expressed genes).

In the present transcriptome data, principal component analysis showed that the samples from each group showed high correlation, and the samples from each treatment group were highly correlated with the expression pattern of the control group (Figure 3a). Venn analysis showed that there were 13,334 co-expressed genes between the control group and the experimental tissues, with genes specific to each group ranging from 31 to 217 (Figure 3b).

**Figure 4:**
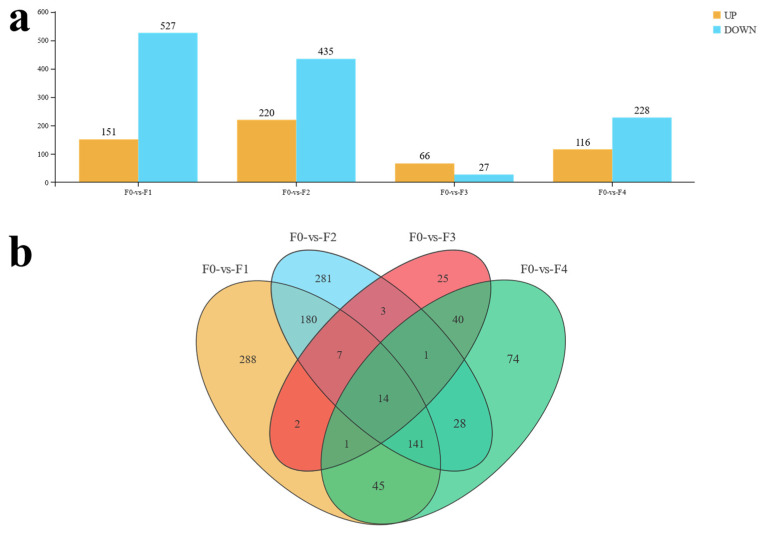
Results of DEGs screening between intestinal transcriptome samples from different treatment groups and controls in MA, including (**a**) baseline analysis of DEGs screened between each treatment group and the control group, and (**b**) Venn analysis of co-expressed and unique DEGs results (groups are filled with different colors, with overlapping portions representing (2 or n) co-expressed DEGs between groups and non-overlapping portions representing the number of DEGs specific to the comparison group; numbers indicate the specific number of DEG).

Figure 4a demonstrates the results of the differential expression analysis of DEGs between F0 and each treatment group in this experiment. Compared with the gene expression levels of the control group, F1 had the highest number of DEGs, with 151 up-regulated genes and 527 down-regulated genes, followed by F2, with a total of 655 DEGs, including 220 up-regulated genes and 435 down-regulated; F4 was enriched with a smaller number of DEGs, which was specifically represented by 116 up-regulated and 228 down-regulated genes; and F3 was enriched with the least number of DEGs, with only 66 up-regulated and 27 down-regulated genes. Veen analysis showed that there were 288, 281, 25, and 74 unique DEGs for each treatment group, respectively, and 14 DEGs were common to the treatment groups (Figure 4b).

**Figure 5:**
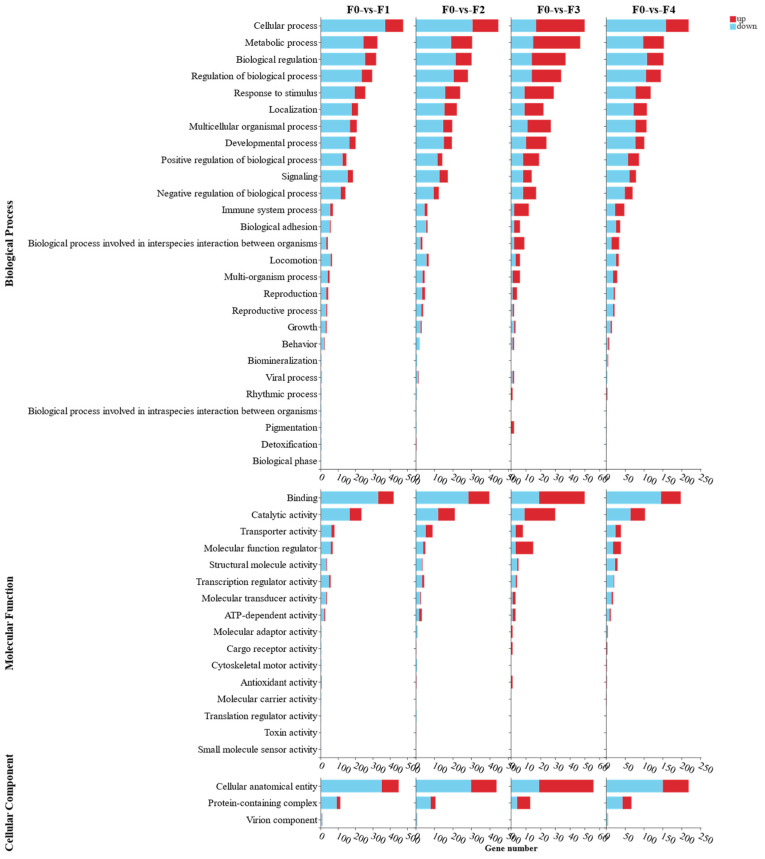
MA intestinal DEGs screened for GO functional annotation followed by primary and secondary GO term identification.

In order to categorize the DEGs enriched in each group, DEGs were mapped to GO terms contained within molecular function (MF), cellular component (CC), and biological process (BP) in the GO database (Figure 5). The significantly enriched GO analysis showed that BP was enriched with the most DEGs, and the main enriched GO terms were the cell process, metabolic process, and biological regulation, followed by MF with the main enriched GO terms binding, catalytic activity, and transporter activity. CC enriched the least DEGs, and the main enriched GO terms were cellular anatomical entity, protein-containing complex, and virion component.

**Figure 6:**
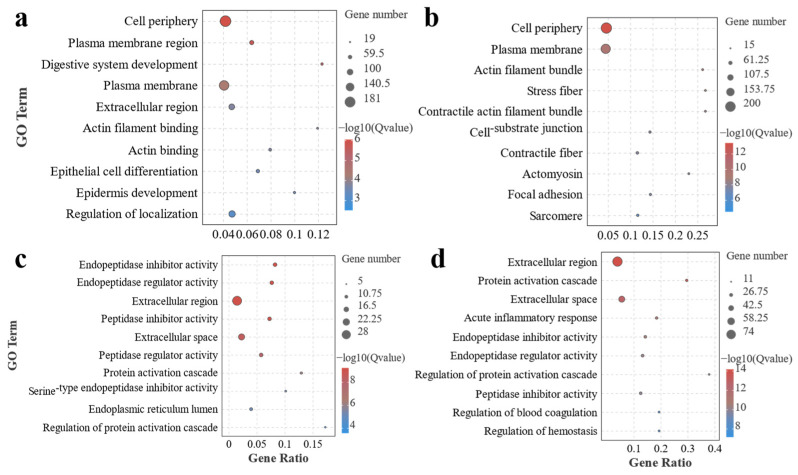
Significant enrichment of GO terms after GO functional annotation of MA gut-screened DEGs in each treatment group. Note: (**a**) F0-vs.-F1; (**b**) F0-vs.-F2; (**c**) F0-vs.-F3; (**d**) F0-vs.-F4.

To further determine the number of DEGs enriched to the relevant GO term in each group, the significance and gene ratio, and other related information, we further demonstrated this result by a significant bubble enrichment plot (Figure 6). This part of the results showed that the enriched GO terms had similar distribution patterns in F0 vs. F1 and F0 vs. F2, and cell periphery, plasma membrane region, and plasma membrane were the main GO terms enriched. In addition, digestive system development was also enriched in F0 vs. F1. In F0 vs. F3 and F0 vs. F4, inflammation-related GO terms seemed to dominate. Endopeptidase inhibitor activity, endopeptidase regulator activity, extracellular region, peptidase inhibitor activity, extracellular space, peptidase regulator activity, and protein activation cascade were the main GO terms enriched in F0 vs. F3. F0 vs. F4 was similar to F0 vs. F3, which was also enriched for extracellular region, protein activation cascade, extracellular space, endopeptidase inhibitor activity, and endopeptidase regulator activity. In addition, acute inflammatory response-related GO terms were also enriched by F0 vs. F4.

**Figure 7:**
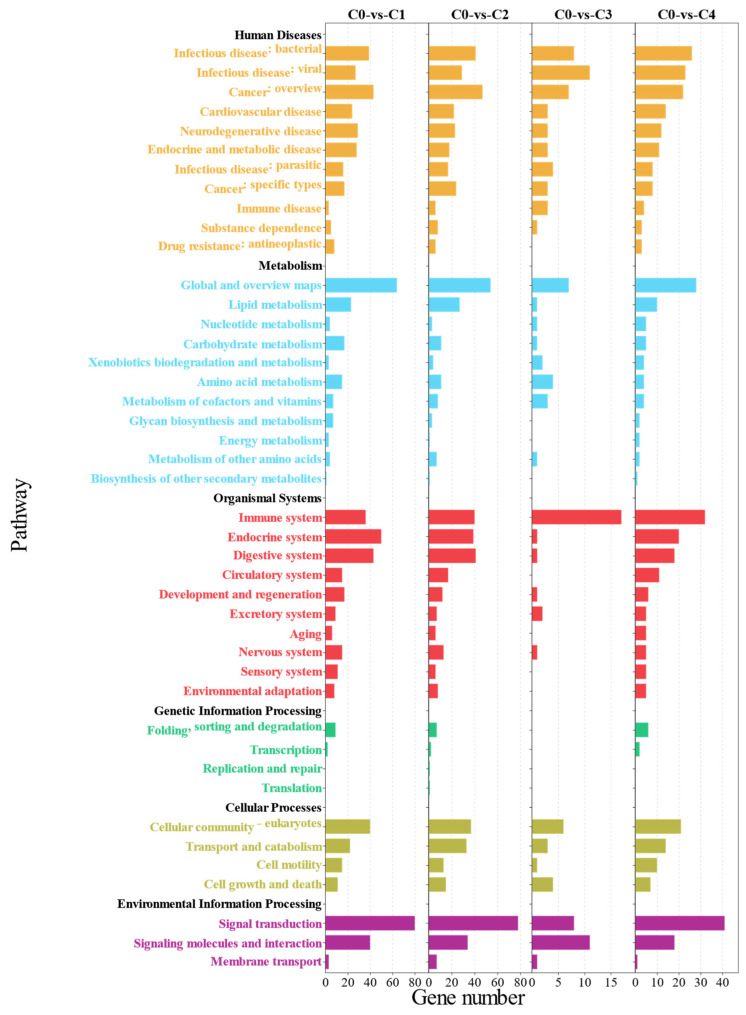
Identification of major class I and class II pathways enriched for KEGG functional annotations for MA intestinal DEGs.

In order to enrich the DEGs of each group to the pathways to which they belong, the DEGs were mapped to the KEGG database for human diseases, metabolism, organismal systems, genetic information processing, cellular processes, and environment information processing, and the significantly enriched pathways are shown (Figure 7). KEGG enrichment analysis showed that human diseases, organismal systems, and metabolism were enriched for the most pathways. Infectious disease: bacterial, infectious disease: viral, cancer: overview, cardiovascular disease, and neurodegenerative disease were the most enriched pathways to DEGs among human disease-related pathways. Organismal systems, the immune system, the endocrine system, and the digestive system are the most enriched pathways. Global and overview maps, carbohydrate global and overview maps, carbohydrates, amino acid metabolism, and lipid metabolism were the most enriched pathways in metabolism related pathways.

**Figure 8:**
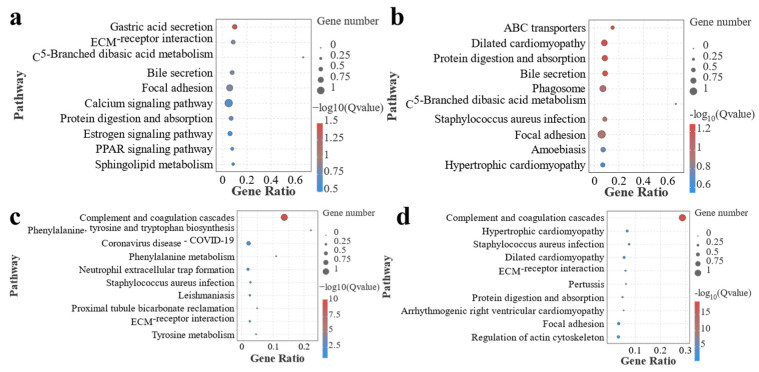
Pathways significantly enriched after annotation of KEGG function in MA intestinal screening DEGs in each treatment group. Note: (**a**) F0-vs.-F1; (**b**) F0-vs.-F2; (**c**) F0-vs.-F3; (**d**) F0-vs.-F4.

To further determine information on the number, significance, and gene ratio of DEGs enriched in the pathway in each group, we selected the top ten FDR values of each group for the resulting significance bubble plots to further visualize this result (Figure 8). The results showed that, similar to the GO analysis, the pathways enriched in F0 vs. F1 and F0 vs. F2 had similar characteristics, with metabolism-related pathways enriched in both groups, and the pathway significantly enriched in F0 vs. F1 was gastric acid secretion. Pathways significantly enriched for F0 vs. F2 were ABC transporters, dilated cardiomyopathy, and protein digestion and absorption. It is noteworthy that bile secretion-related pathways were enriched in both of the above groups. In F0 vs. F3 and F0 vs. F4, inflammation-related pathways were significantly enriched, and complement and coagulation cascades were the most significantly enriched pathways in both groups.

### 3.3. Metabolome Analysis of Effects of Different Ratios of SPP on MA’s Intestine

At least six samples from each of the five groups were tested in the metabolomics assay, and two ionization modes, positive ion mode (POS) and negative ion mode (NEG), were used for the detection of the samples. The results of the assay were combined with public databases such as Mass Bank, Metlin, and MoNA paired with self-constructed secondary mass spectrometry databases to match for substance annotation, and finally, a total of 6981 metabolites were obtained in the POS mode, with 1500 being accurately annotated, and 5770 metabolites were detected in the NEG mode, with 1317 finally being annotated (Table 3).

**Figure 9:**
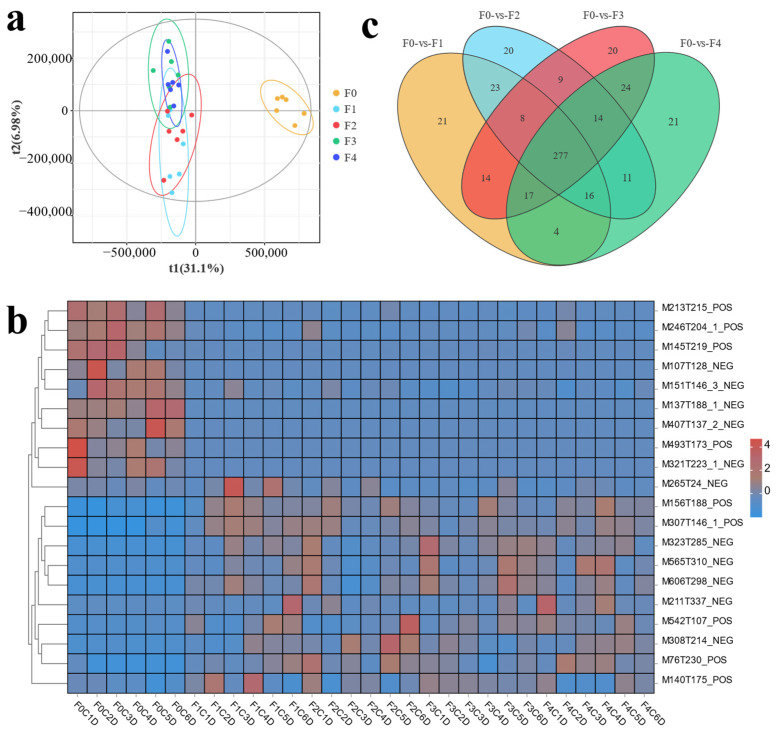
Sample relationships and differential metabolite screening results between intestinal metabolome samples from different treatment and MA control groups, including (**a**) PLS-DA-based analyses revealing sample relationships between each treatment and control group, ovals represent 95% confidence intervals and the same color is used for samples in the same group. (**b**) heat maps of metabolite clustering between samples (groups are filled with different colors, with overlap representing differential metabolites shared between (2 or n) comparator groups, and each non-overlap representing the number of differential metabolites specific to the comparator group; numbers indicate the specific number of shared and specific metabolites), and (**c**) Veen analyses revealing abundance of shared and unique differential metabolites between each treatment and control group.

In the present metabolome data, PLS-DA analysis showed that the samples from each treatment group showed high correlation and low correlation with F0 (Figure 9a). The clustering heatmap showed significant differences in metabolite composition between the treatment and F0 (Figure 9b). Venn analysis showed 277 co-metabolites between the control and experimental tissues and 20–21 metabolites specific to each group (Figure 9c).

**Figure 10:**
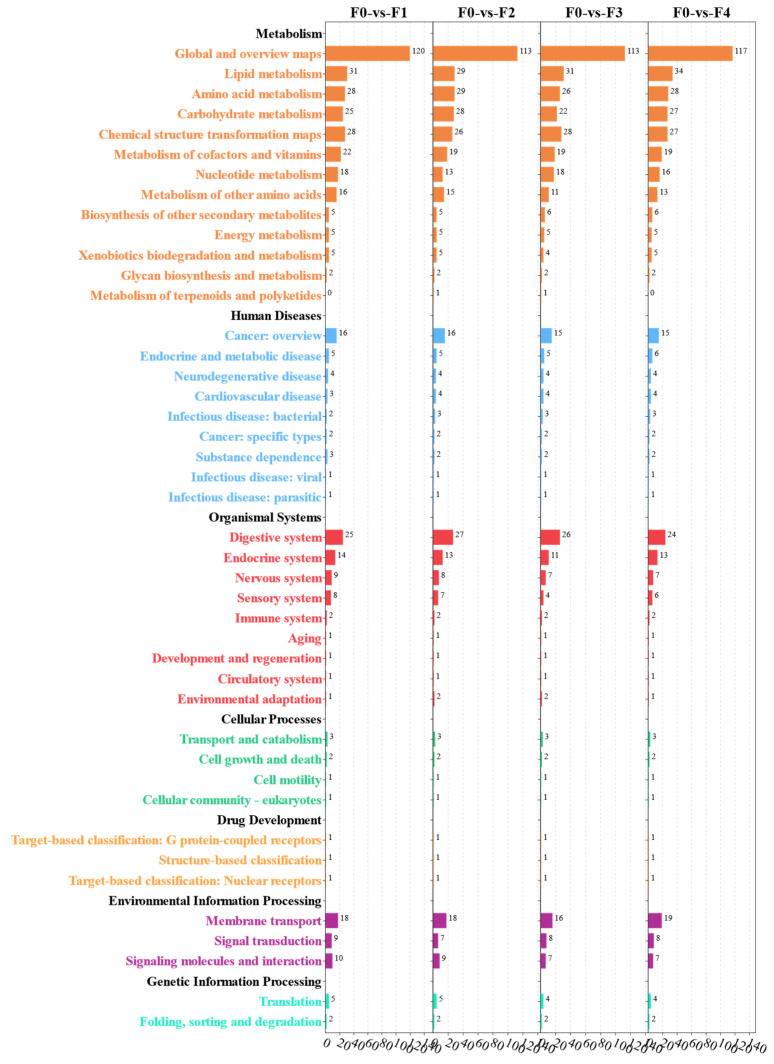
Major class I and class II pathways for different metabolites of the MA intestinal metabolome of the treatment group identified by KEGG functional annotation.

In order to enrich the differential metabolites of each group into the pathways to which they belonged, the metabolites were mapped to the pathways included within metabolism, organismal systems, cellular processes, drug development, genetic information processing, and environmental information processing in the KEGG database, and the results are shown in Figure 10. The analysis showed that metabolism was enriched by the most pathways, and most of the pathways were enriched in global and overview maps, amino acid metabolism, carbohydrate metabolism, and lipid metabolism-related pathways. The predominantly enriched pathway for human disease was cancer: overview. The organismal systems enriched by the most pathways were the digestive system, endocrine system, nervous system, and sensory system. Many pathways in environmental information processing were enriched for membrane transport, signal transduction, signaling molecules, and interaction.

**Figure 11:**
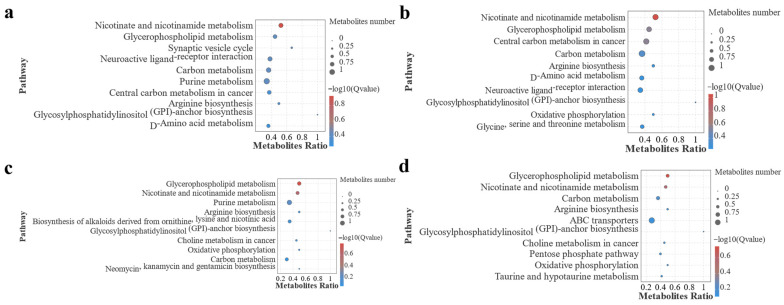
Pathways significantly enriched after functional annotation of the MA intestinal screen for the differential metabolite KEGG in each treatment group. Note: (**a**) F0-vs.-F1; (**b**) F0-v.s-F2; (**c**) F0-vs.-F3; (**d**) F0-vs.-F4.

To determine the number, significance, and metabolite ratio of metabolites enriched to the pathways in each group. We selected the pathways with the top ten FDR values in each group and this result is further visualized by a significance bubble plot (Figure 11). From the results, nicotinate and nicotinamide metabolism and glycerophospholipid metabolism were the two pathways that were significantly enriched in each group.

### 3.4. Co-Analysis of Transcriptome and Metabolome Reveals the Effects of Different Proportions of Spirulina on MA’s Intestine

**Figure 12:**
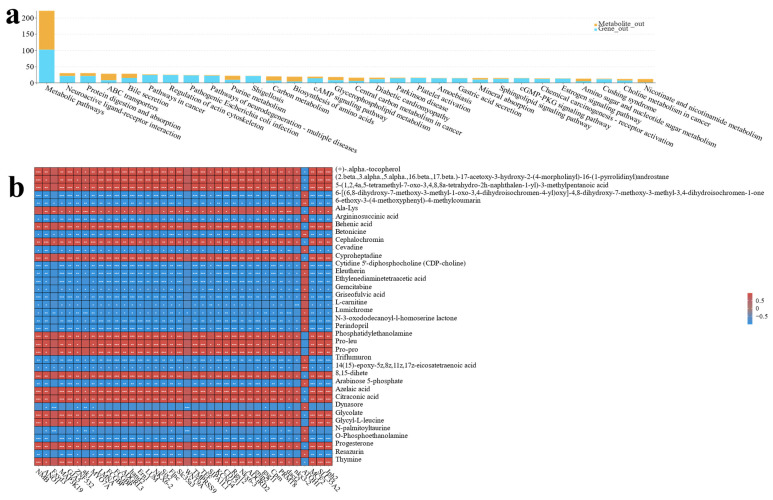
Results of the correlation analysis between the MA gut transcriptome and metabolome, including (**a**) the top 30 pathways significantly enriched in the correlation analysis shown in the bar charts, and (**b**) the annotated heatmap of the correlation between differential metabolites and DEGs, pairs of correlations that satisfy statistical significance are marked with an “*”, with a greater number of asterisks indicating a more significant correlation.

DEGs and differential metabolites were jointly analyzed (Figure 12a). Key enriched pathways included metabolic, neuroactive ligand–receptor interactions, and protein digestion. The correlation analysis showed interactions between genes and metabolites, both positive and negative (Figure 12b). Significant metabolites were mainly carboxylic acids, amino acids, and lipids (Supplementary Material S2 Table S3). Most genes related to environmental information processing were highly enriched (Supplementary Material S2, Table S4).

### 3.5. Validation of the Screened mRNAs by RT-qPCR

**Figure 13:**
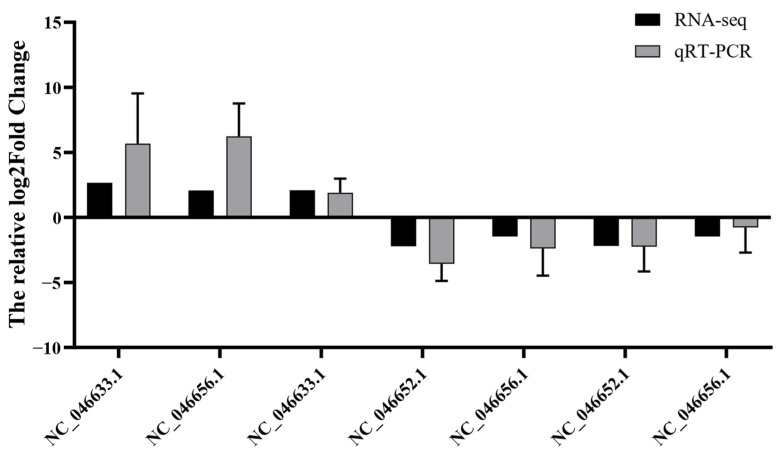
Transcriptome target gene validation results.

To verify the accuracy of RNA-seq in this study, some of the DEGs in the sequencing results were randomly selected to detect their actual expression by relative fluorescence quantification using the *β*-actin gene as an internal reference gene (Figure 13). As shown, the relative fluorescence quantification results of all DEGs showed the same trend as the sequencing results in the transcriptome. Therefore, the RNA-seq results of the transcriptome in this study are accurate and reliable.

## 4. Discussion

### 4.1. Effect of Different Ratios of SPP on Intestinal and Serum Antioxidant Capacity of MA

In aquaculture, fish are often exposed to a variety of stress conditions due to farming methods and environmental factors, and the generation of reactive oxygen species (ROS) resulting from the effects of extreme stresses leads to DNA, protein, and lipid damage [45]. Antioxidant enzyme activity is closely related to the health of fish, and when the dynamic balance between ROS production and antioxidant defense is out of balance, the antioxidant enzyme activity becomes an important indicator of responses to oxidative stress [46]. In modern, high-density farming models, animals consistently experience more severe stress than their wild peers [47]. The use of artificial feeds reduces the cost of feeding aquatic animals and facilitates a more precise provision of optimal nutrients to meet the growth requirements of the animals, but unsuitable feed ingredients or formulation compositions often lead to adverse reactions such as oxidative stress after consumption by animals [48,49]. Zhang et al. showed that a high percentage of soybean meal resulted in intestinal inflammation in fish, which negatively affected intestinal physiological indicators and growth performance [14]. Despite the large amount of data supporting SPP as an aquafeed ingredient full of potential for the future, there are still studies showing that there is an upper limit to the use of SPP in different species; otherwise, it can negatively affect fish productivity and alter product quality [17,50]. Aquaculture is an industry that covers many species, and different species are constrained by their own physiological structure and characteristics to utilize SPP at different levels, and the optimal level of SPP in feed formulations for different species also varies. The study by Akter et al. confirmed that the optimal percentage of SPP in the recipe of *Ompok pabda* is 15% [51]; the study by Rosas et al. confirmed that in the presence of flaxseed oil addition, SPP replacement of 50% of fishmeal in the recipe of Mugil liza feed is feasible [52]; and SPP can replace up to 72.03% of fishmeal in the recipe of *Pelteobagrus fulvidraco* feed according to the results of the experiment of Liu et al. [53]. The experimental results of this study show that among the physiological indicators of the intestine, the intestinal stress of MA increased with the rise of SPP addition (SOD decreased and MDA increased), which suggests that there is an optimal ratio of SPP addition between F2 and F3.

From the results of this part of the experiment, the excessive addition of SPP resulted in intestinal stress in MA, but the stress did not affect the serum indexes of MA, which suggests that there is a pathway between intestinal and serum to alleviate the stress. In serum physiological indices, SOD increased with the rise of SP substitution, and F3 produced a significant difference with F0, indicating that a 15% substitution level is more appropriate in feed formulation for MA. This part of the experiment proved that the optimal percentage of fishmeal replacement by SPP is between 10 and 15%, without affecting the health of MA.

### 4.2. Transcriptomics Reveals the Effects of Different Ratios of SPP on the Intestine of MA

Transcriptomics is an effective method for identifying differential gene expression in animal organisms under stress [54]. Transcriptome-based analyses can explain the potential mechanisms of physiological effects on fish after feed formulation changes [55]. The intestine is the main organ for digestion and absorption in fish, and studies on the effects of SPP replacement of fishmeal on the MA intestine have not been published; therefore, we used RNA sequencing to generate transcriptome information and analyze the effects of feed formulation changes on MA intestinal gene expression [14]. In the results of this experiment, the enriched DEGs showed two types in both GO and KEGG analyses, i.e., a low substitution group thought to be related to major cell growth and metabolism, represented by F0 vs. F1 and F0 vs. F2, and a high substitution group related to inflammation, represented by F0 vs. F3 and F0 vs. F4.

Peptidases-related GO terms are among the more frequently mentioned GO terms in GO analyses, and peptidases catalyze the degradation of peptide bonds in different biological processes and are a large class of hydrolases present in all organisms [56]. Peptidases are involved in the degradation of dysfunctional proteins in lysosomes, cytoplasmic lysates, plasma membranes, or the extracellular space and may also have regulatory roles in the control of biological processes critical to cellular homeostasis. Several of their functions have been implicated in several pathological processes, in addition to their involvement in normal protein renewal [57]. Only peptidases contain digestive metabolism-related trypsin and chymotrypsin, but KEGG analyses, enriched for many complement-related pathways in the DEGs of these F0 vs. F3 and F0 vs. F4 groups, and thus the peptidase-related GO term of this fraction may be associated with inflammation in the intestine.

KEGG analysis showed that pathways related to digestive metabolism were significantly enriched in F0 vs. F1 and F0 vs. F2, suggesting that the addition of SPP modulated the digestive and absorptive capacity of MA, whereas the enrichment of the gastric acid secretion and bile secretion pathways implied that altered secretion of digestive juices underpinned this enhanced capacity. Abdel-Latif et al. showed that SPP increased the activity of intestinal digestive enzymes in Liza ramada, and the culture results also confirmed that the growth index of Liza ramada increased after SPP addition [58]. Studies on *Huso huso* also showed that the addition of SPP significantly increased protease and lipase, as compared to control group activities [59]. This suggests that fish can adapt to the addition of SPP to their recipes by mobilizing the secretion levels of digestive enzymes. KEGG analysis showed that the F0 vs. F3 and F0 vs. F4-enriched pathways were concentrated in the complement and coagulation cascades. The activation of the complement system in fish subjected to stress leads to the assembly of complement complexes, which induces complement-mediated cytolysis [60]. SPP is an immunostimulant for boosting fish immunity levels [61,62]. Immunostimulants stimulate natural killer cells, complement, lysozyme activity, and antibody responses in fish and shellfish primarily by promoting phagocytosis, thereby enhancing the killing of pathogens and resistance to infectious diseases [63]. The addition of SPP to the feed formulation significantly increased lysozyme, respiratory burst activities, immunoglobulin, and complement levels in *Plectropomus leopardus*, which exhibited higher survival rates after attack by *V. harveyi* [64]. This is consistent with the significant upregulation of the complement and coagulation cascade pathways in this study. This part of the experiment demonstrated that MA can adapt to feed formulation after SPP replaces a low percentage of fishmeal by elevating the secretion level of digestive enzymes and that a high percentage of SPP replaces fishmeal, mobilizing the complement level in the MA intestine.

Combined with the results of the physiological indicators of the intestinal tract, this suggests that the replacement of a lower percentage (10–15%) of fishmeal by SPP is the ideal formula for both growth and immunity.

### 4.3. Metabolomics Reveals the Effects of Different Ratios of SPP on the Intestine in MA

In previous experiments, the replacement of fishmeal by different ingredients often resulted in corresponding changes in the animal organism, which brought about changes in metabolic products, and the changes in the products often implied that the animals were adapted to the new feed formulation [65,66]. This change is closely related to the growth and muscle quality of the animal. For instance, largemouth bass (*Micropterus salmoides*) showed adverse effects on growth and muscle quality following the substitution of soybean meal for 60% of fishmeal. There was also a notable enrichment of differential metabolites in the pathways of biosynthesis of amino acids, protein digestion and absorption, D-arginine and D-ornithine metabolism, histidine metabolism, alanine, aspartate, and glutamate metabolism [67]. Chen et al. found that the metabolism of glycine, serine, and threonine, as well as glutathione metabolism, influenced the efficacy of fermented soybean meal as a replacement for fishmeal in largemouth bass (*Micropterus salmoides*) culture [65]. SPP and fishmeal differ considerably in their amino acid and fatty acid compositions, and the difference in composition means that MA needs to adapt to this aspect of the change and adapt to the new nutritional source at a physiological level, which also leads to differences in metabolites.

Nicotinate and nicotinamide metabolism was one of the significantly altered pathways observed. Nicotinate and nicotinamide are NAD+ precursors that are closely related to vitamin B3, and NAD+ is involved in many fundamental cellular processes as a cofactor in a variety of metabolic redox reactions, as well as a specialized co-substrate for NAD+-depleting enzymes, which is important for cellular homeostasis [68]. For example, NAD+ supplementation restored mitochondrial and ciliary dysfunction in ARMC9-deficient cells and zebrafish [69]. Inhibition of NAD+ biosynthesis in zebrafish (*Danio rerio*) leads to mitochondrial function in oocytes, thereby impairing oocyte maturation and ovarian fertility [69]. There are three synthetic pathways in vivo, i.e., NAD+ can be synthesized via three major biosynthetic pathways: (1) the de novo pathway or kynurenine pathway from tryptophan; (2) the Preiss–Handler pathway from niacin; and (3) the salvage pathway from nicotinamide [70]. Among the metabolites examined in the metabolome, nicotinamide was detected as a rise in synthesis in all experimental groups, implying that the addition of SPP promoted nicotinamide synthesis in MA. Dong et al. showed that the administration of NAD+ precursors modulated liver injury and metabolic dysfunction in the doughnut bream [71]. Differences in synthesis pathways can be attributed to differences in amino acid composition and vitamins between SPP and fishmeal [72]. The increase in nicotinamide synthesis suggests that SPP supplementation may support cellular energy balance and homeostasis in MA.

Glycerophospholipid is a structural component of cell membranes, and glycerophospholipid metabolism is a major pathway involved in systemic immunity and low-grade inflammatory states, suggesting that phospholipids are potential mediators of inflammation [73,74]. Microplastic fibers upregulate glycerophospholipid metabolism in zebrafish (*D. rerio*) exposed to microplastics, aggravating oxidative damage and inflammation while downregulating fatty acid metabolism linked to nutritional deficiencies [75]. In the metabolic pathway, we observed an increase in the synthesis of lecithin and ceruloplasmin, representing an enhanced resistance of MA to inflammation. This may be attributed to the strong ability of MA to metabolize glycerolipids and biosynthesize unsaturated fatty acids, which are also present in mandarin fish (*Siniperca chuatsi*) adapted to artificial diets [48]. Among the metabolic pathways, we found a decrease in the experimental group while citrulline, which is a precursor of arginine and is considered a marker of intestinal injury [76].

The results of this part of the trial confirmed that SPP addition mainly affects nicotinic acid and nicotinamide metabolism and glycerophospholipid metabolism in the intestinal tract of MA and does not change with the rate of addition. Intestinal citrulline content was reduced by inflammation as affected by excessive (>10%) SPP additions, but feeding SPP increased the glycerophospholipid content of the MA intestinal tract, accompanied by changes in niacin and nicotinamide metabolism, which mitigated the effects of inflammation on the intestines. This result is also consistent with physiological indicators of the intestine and the results of the transcriptome GO analysis.

### 4.4. Combined Transcriptomics and Metabolomics Analysis Reveals the Effects of Different Ratios of SPP on the Intestine of MA

Transcriptomics and metabolomics reflect the level of adaptation to altered feed formulations in terms of changes in gene expression levels and metabolites following changes in fish feed formulations [77]. Fatty acids and amino acids affect gene expression in fish and, thus, their own metabolism [78,79]. Alterations in signaling pathway changes affect metabolic activity in the reverse direction, so amino acid and fatty acid metabolism in vivo will be altered as a result of changes in feed formulation [67,80]. The replacement of fishmeal by Spirulina changed the amino acid and fatty acid composition of the feed and therefore affected the intestinal metabolic function of MA, which led to enrichment of the metabolome and transcriptome for pathways and metabolites related to protein and lipid digestive metabolism, and linkage analyses enriched pathways related to amino acid and fatty acid metabolism, suggesting that MA could be adapted to feed formulations following the replacement of fishmeal by SPP.

## 5. Conclusions

In this study, we investigated the effects of SPP replacement of fishmeal on MA intestinal metabolism through physiological indicators, transcriptomics, and metabolomics. The results showed that MA could adapt to the feed formulation after SPP replacement of fishmeal, and the low percentage (10–15%) of replacement levels could significantly promote the fatty acid and amino acid metabolism levels of MA intestine, while the high percentage (>15%) would lead to inflammation of the intestine. The intestinal citrulline content was decreased in the high-replacement group as a result of inflammation, but hydrochloric acid and nicotinamide metabolism and glycerophospholipid metabolism linked the inflammatory response in the intestines. The present study showed that a low percentage of SPP replacement in fishmeal has a positive effect on MA health, and the replacement percentage should be between 10 and 15% in terms of intestinal health.

## Figures and Tables

**Table 1:** Primers sequences for qRT-PCR.

Primer	Forward	Sequences (5′ to 3′)
*β*-actin	F	TGAGCGTGAGATTGTGCGT
	R	AAGAGGGCTGGAAAAGGGA
NC_046633.1	F	TCACTCGTGCTGTTGACTTAC
	R	TAGAAGAAGGAGAGGAGGAAGG
NC_046656.1	F	CCTGGAGTGTGTAAATGGAGTAG
	R	GGACTGTGTGGGAACTTTGA
NC_046636.1	F	CGTTGTGCTGTGGGAGATAA
	R	GTGGATGGAAGGAGCAGAAA
NC_046633.1	F	TGCTGCTGTGTCTGTCTATG
	R	GTTACCTACTCGGGCATCTATTT
NC_046652.1	F	ACTAGGCTGTCAAGTGCTAATG
	R	GCATCGCTGGAAGGATAACT
NC_046656.1	F	CCTAACCTCAACCCACCAATTA
	R	GCAGAGAGGAAAGGAGAGAATG
NC_046652.1	F	TGGTGATCGGTCGTCTTTATTC
	R	TGGGCTCTTTGTGGTCTTTAC
NC_046656.1	F	CTCCACTCTCCATTCGCTTATC
	R	CAAGGCTATCTCCACAACTACC

F: (sense primer); R: (antisense primer).

**Table 2:** Statistical data of intestinal transcriptome sequencing in MA.

Sample	Raw Data (G)	Clean Data (G)	Q20 (%)	Q30 (%)	Total Reads	Total Mapped (%)
Z0C1	6.27	6.15	97.71	95.74	44,534,846	80.28
Z0C2	5.66	5.54	97.39	95.23	40,183,868	82.54
Z0C3	6.32	6.17	97.72	95.75	44,853,084	79.93
Z1C1	6.4	6.29	97.76	95.84	45,431,520	77.74
Z1C2	6.25	6.12	97.73	95.78	44,361,932	77.74
Z1C3	6.02	5.91	97.43	95.3	42,725,926	77.07
Z2C1	5.48	5.37	97.8	95.91	38,839,870	78.89
Z2C2	5.79	5.62	97.53	95.49	41,011,036	80.93
Z2C3	5.22	5.12	97.71	95.72	36,995,762	78.32
Z3C1	5.12	4.98	96.73	94.11	36,307,444	79.85
Z3C2	5.38	5.26	97.91	95.98	38,208,560	78.76
Z3C3	5.39	5.3	97.99	96.14	38,301,174	82.47
Z4C1	5.47	5.36	96.88	94.37	38,794,138	79.61
Z4C2	5.55	5.45	98.06	96.27	39,386,622	79.37
Z4C3	5.81	5.69	97.06	94.75	41,189,022	78.64
Z5C1	5.57	5.46	97.66	95.64	39,503,432	79.36
Z5C2	6.67	6.54	97.68	95.67	47,359,346	79.38
Z5C3	5.64	5.53	97.44	95.33	40,076,522	78.84

**Table 3:** Statistical data of intestinal metabolome sequencing statistics in MA.

Type	All	Known	Unknown
POS	6981	1500	5481
NEG	5770	1317	4453

## Data Availability

The authors declare that the data supporting the findings of this study are available within the article and its Supplementary Information Files.

## Supplementary Materials

The following supporting information can be downloaded at https://www.mdpi.com/article/10.3390/antiox13070851/s1, Table S1 Experimental feed formulation and nutrient composition; Table S2 Growth performance and feed utilization of MA fed different fish meal replacement diets for 10 weeks; Table S3 Metabolites of Co-analysis; Table S4 Genes of Co-analysis.
