# Supplementary material for: Multi-Omics Reveals the Effects of Spirulina platensis Powder Replacement of Fish Meal on Intestinal Metabolism and Stress in Zig-Zag Eel (Mastacembelus armatus)
Source: Antioxidants (Basel). 2024 Jul 15;13(7):851. doi: 10.3390/antiox13070851 (PMC11273650; doi:10.3390/antiox13070851)
Supplement: Supplementary file 1 [file antioxidants-13-00851-s001.zip › antioxidants-3099608-supplementary.pdf]

Table S1 Experimental feed formulation and nutrient composition

Raw material %	F0	F1	F2	F3	F4
Fishmeal	67	63.65	60.3	56.95	53.6
Fermented Soybean Meal	7	7	7	7	7
SPP	0	3.2	6.41	9.62	12.82
$Ca(H_2PO_4)_2$	1	1	1	1	1
Multi-Vitamin [1]	0.1	0.1	0.1	0.1	0.1
Multi-Mineral [2]	0.8	0.8	0.8	0.8	0.8
Choline Chloride	0.2	0.2	0.2	0.2	0.2
Vitamin C	0.1	0.1	0.1	0.1	0.1
Fish Oil	0.42	0.69	0.97	1.24	1.52
Microcrystalline Cellulose	1.38	1.26	1.12	0.99	0.86
Pregelatinized Starch	22	22	22	22	22
Base Ingredients (dry weight %)					
Dry matter	93.17	93.34	93.53	93.71	93.89
Crude protein	50.13	50.14	50.52	50.68	49.43
Crude fat	5.51	5.66	5.38	5.84	5.89
Crude Ash	10.93	11.94	11.6	11.26	10.93
Total energy (MJ*kg-1)	19.33	19.18	19.28	19.37	19.46

[1] Vitamin contains: Vitamin A (3000000 IU /kg); Vitamin D (1500000 IU /kg); Vitamin E (15 g/kg); Vitamin K (10 g/kg); Vitamin B1 (10 g/kg); Vitamin B2 (15 g/kg); Vitamin B6 (15 g/kg); Vitamin B12 (60 mg/kg); Inositol (70 g/kg); Niacinamide (40 g/kg); Calcium Pantothenate (40 g/kg); Biotin (100 mg/kg); Folic acid (1.8 g/kg).
[2] Minerals contains: Iron (3000 g/kg); Manganese (750 mg/kg); Iodine (120 mg/kg); Copper (3 g/kg); Zinc (7 g/kg); Selenium (35 mg/kg); Cobalt (100 mg/kg)

Table S2 Growth performance and feed utilization of MA fed different fish meal replacement diets for 10 weeks. [1]

Diet [2]	F0	F1	F2	F3	F4
Initial weight (g)	1.04±0.08 [a]	1.04±0.01 [a]	1.01±0.02 [a]	1.05±0.02 [a]	1.01±0.03 [a]
Feed intake/kg	2.33±0.23 [a]	2.34±0.2 [a]	2.24±0.13 [a]	1.95±0.24 [ab]	1.67±0.08 [b]
WGR (%)	7.88±1.74 [b] [3]	10.81±1.55 [ab]	12.16±1.01 [a]	10.5±1.59 [ab]	9.31±0.69 [ab]
SGR (%)	3.23±0.28 [b]	3.67±0.2 [ab]	3.84±0.11 [ab]	3.63±0.2 [ab]	3.48±0.1 [ab]
FCR	2.69±0.48 [a]	2.1±0.19 [ab]	1.82±0.01 [b]	1.78±0.07 [b]	1.78±0.09 [b]
SR (%)	83.33±26.01 [a]	91.67±11.96 [a]	90±5.4 [a]	91.67±7.73 [a]	88.33±3.12 [a]
VSI (%)	6.48±0.35 [a]	6.91±0.06 [a]	7.17±0.05 [a]	6.44±0.23 [a]	6.59±0.72 [a]
HSI (%)	1.79±0.09 [a]	1.99±0.04 [a]	1.96±0.27 [a]	1.85±0.14 [a]	1.64±0.28 [a]
CF (%)	0.29±0.01 [a]	0.3±0.01 [a]	0.29±0.01 [a]	0.32±0.03 [a]	0.29±0.01 [a]
CP (%)	93.52±0.35 [a]	93.09±0.06 [a]	92.83±0.05 [a]	93.56±0.23 [a]	93.41±0.72 [a]

[1] Values are mean ± SD; n = 9, a significance level of $P < 0.05$ was used for all statistical tests.

[2] For diet details, please refer to methods Section 2.1 Fish and Feed.

[3] Different superscript letters represent significant differences, $p < 0.05$.

Table S3 Metabolites of Co- analysis

Compound_ID	MS2_name	F0	F1	F2	F3	F4	RT	m/z	SuperClass	Class	SubClass	CAS	HMDB	C_ID	Pathway	KEGG_B_class	KEGG_A_class
M243T212_2_POS	Lumichrome	45963827	2.05E+08	2.01E+08	1.31E+08	1.89E+08	3.536	243.0952	Organoheterocyclic compounds	Pteridines and derivatives	Alloxazines and isoalloxazines	-	HMDB0254199	C01727	ko00740//Riboflavin metabolism	Metabolism of cofactors and vitamins	Metabolism
M228T50_NEG	Resazurin	1188978	58751719	64228080	60291092	67186345	0.832	228.0338	Organoheterocyclic compounds	Benzoxazines	Phenoxazines	-	HMDB0257158	-	-	-	-
M125T53_NEG	Thymine	3.35E+08	24210863	12325965	21548561	21415826	0.892	125.0358	Organoheterocyclic compounds	Diazines	Pyrimidines and pyrimidine derivatives	65-71-4	HMDB0000262	C00178	ko01100//Metabolic pathways;ko00240//Pyrimidine metabolism	Global and overview maps;Nucleotide metabolism	Metabolism
M229T305_NEG	Arabinose 5-phosphate	465	552	587	487	617	5.0	229	Organic oxy	Organooxygen	Carbohydrates and carbohydrate	13137-52-5	HMDB0011734	C01112	ko01100//Metabolic pathways	Global and overvie	Metabolism

		799.3	89880	75739	57446	60427	88	.0121	gen compounds	compounds	conjugates					w maps	
M162T243_2_POS	L-carnitine	1.06E+09	1.9E+09	2.02E+09	1.82E+09	1.57E+09	4.057	162.1123	Organic nitrogen compounds	Organonitrogen compounds	Quaternary ammonium salts	541-15-1	HMDB0000062	C00318	ko04976//Bile secretion;ko05415//Diabetic cardiomyopathy;ko04714//Thermogenesis	Cardiovascular disease;Digestive system;Environmental adaptation	Human Diseases;Organismal Systems
M218T312_POS	Ala-Lys	915454489	16272127	13163854	53717940	27910468	5.198	218.1499	Organic acids and derivatives	Carboxylic acids and derivatives	Amino acids, peptides, and analogues	6366-77-4	HMDB0028692	-	-	-	-
M291T336_POS	Argininosuccinic acid	9735597.9	1.71E+08	1.5E+08	1.16E+08	1.47E+08	5.595	291.1299	Organic acids and derivatives	Carboxylic acids and derivatives	Amino acids, peptides, and analogues	2387-71-5	HMDB0000052	C03406	ko01100//Metabolic pathways;ko01110//Biosynthesis of secondary metabolites;ko0123	Amino acid metabolism;Global and overview maps	Metabolism

															0//Biosynthesis of amino acids;ko00250//Alanine, aspartate and glutamate metabolism;ko00220//Arginine biosynthesis			
M160T177_POS	Betonicine	17458136	2.74E+08	3.14E+08	2.14E+08	2.63E+08	2.947	160.0964	Organic acids and derivatives	Carboxylic acids and derivatives	Amino acids, peptides, and analogues	515-25-3	HMDB0029412	-	-	-	-	
M293T323_POS	Ethylenediaminetetraacetic acid	9783918	80974683	81095638	81327344	77362253	5.381	293.098	Organic acids and derivatives	Carboxylic acids and derivatives	Tetracarboxylic acids and derivatives	-	HMDB0303556	-	-	-	-	
M369T	Perindopri	1	3	3	3	3	8	3	Org	Carbo	Amino acids,	107133-	HMDB001492	-	-	-	-	

486_POS	l	48076.5	7195375	6716603	3124692	5849623	.098	69.2496	anic acids and derivatives	xylic acids and derivatives	peptides, and analogues	36-8	8				
M229T178_POS	Pro-leu	2.56E+08	69145145	66471777	69726605	84701340	2.965	229.1546	Organic acids and derivatives	Carboxylic acids and derivatives	Amino acids, peptides, and analogues	-	HMDB0253028	-	-	-	-
M213T299_POS	Pro-pro	54362410	9318765	8164792	7765820	11003968	4.978	213.1234	Organic acids and derivatives	Carboxylic acids and derivatives	Amino acids, peptides, and analogues	20488-28-2	HMDB0011180	-	-	-	-
M75T184_NEG	Glycolate	46070588	9605656	8913328	12119686	9145767	3.074	75.00886	Organic acids and derivatives	Carboxylic acids and derivatives	Amino acids, peptides, and analogues	-	HMDB0304424	C00160	ko01100//Metabolic pathways;ko01110//Biosynthesis of secondary metabolites;ko0112	Carbohydrate metabolism;Global and overview maps	Metabolism

															0//Microbial metabolism in diverse environments;ko01200//Carbon metabolism;ko00630//Glyoxylate and dicarboxylate metabolism		
M187T158_NEG	Glycyl-L-leucine	52649059	1155788	1678713	1752232	6010474	2.636	187.109	Organic acids and derivatives	Carboxylic acids and derivatives	Amino acids, peptides, and analogues	-	HMDB0028929	-	-	-	-
M140T327_NEG	O-Phosphoethanolamine	938880.	1.12E+0	1.04E+0	9757631	1.01E+0	5.445	140.011	Organic acids and derivati	Organic phosphoric acids and derivat	Phosphate esters	1071-23-4	HMDB0000224	C00346	ko01100//Metabolic pathways;ko00564//Glycerophospholipid	Global and overview maps;Glycan biosynth	Environmental Information Processing;Metabolism

		7	8	8	4	8		9	ves	ives					metabolism;ko04071//Sphingolipid signaling pathway;ko00600//Sphingolipid metabolism;ko00563//Glycosylphosphatidylinositol (GPI)-anchor biosynthesis	esis and metabolism;Lipid metabolism;Signal transduction	
M264T274_1_POS	Gemcitabine	899640.2	35074120	44235974	27317025	26234541	4.573	264.0972	Nucleosides, nucleotides, and analogues	Pyrimidine nucleosides	Pyrimidine 2'-deoxyribonucleosides	95058-81-4	HMDB0014584	-	-	-	-
M358T36_2_POS	Behenic acid	742	217	185	272	204	0.6	358	Lipids and	Fatty Acyls	Fatty acids and conjugates	-	HMDB0304269	C08281	ko01040//Biosynthesis of	Lipid metabolism	Metabolism

							06		lipid-like molecules						unsaturated fatty acids		
M492T212_POS	Cevadine	29287970956.8	7488139808170	4756531705711	0922223901508	6272935197345	3.541	.3678492.2929	Lipids and lipid-like molecules	Steroids and steroid derivatives	Steroidal alkaloids	-	HMDB0259783	-	-	-	-
M749T106_POS	Phosphatidylethanolamine	43897006	4692697	8236764	7714164	5941786	1.769	748.5826	Lipids and lipid-like molecules	Glycerophospholipids	Glycerophosphoethanolamines	-	HMDB0060501	C00350	ko01100//Metabolic pathways;ko01110//Biosynthesis of secondary metabolites;ko00564//Glycerophospholipid metabolism;ko04723//Retrograde endocannabinoid signaling;	Global and overview maps;Glycan biosynthesis and metabolism;Infectious disease: bacterial;Infectious disease: viral;Lipid metaboli	Cellular Processes;Human Diseases;Metabolism;Organismal Systems

															ko05167//Kaposi sarcoma-associated herpesvirus infection;ko00563//Glycosylphosphatidylinositol (GPI)-anchor biosynthesis;ko04140//Autophagy - animal;ko04136//Autophagy - other;ko05130//Pathogenic Escherichia coli infection	sm;Nervous system;Transport and catabolism	
M317T36_NEG	14(15)-epoxy-5z,8z,11z,17z-eicosatetra	12060	1.03E	1.36E	76062	1.23E	0.597	317.2	Lipids and lipid-like	Fatty Acyls	Fatty acids and conjugates	-	HMDB0010205	-	-	-	-

	enoic acid	713	+08	+08	292	+08		125	molecules								
M335T73_NEG	8,15-dihete	58330288	2290530	3654717	5055615	5371379	1.224	335.2209	Lipids and lipid-like molecules	Fatty Acyls	Eicosanoids	77667-08-4	HMDB0010219	-	-	-	-
M187T208_2_NEG	Azelaic acid	1.07E+08	12079196	12114172	19013438	11901519	3.468	187.0726	Lipids and lipid-like molecules	Fatty Acyls	Fatty acids and conjugates	123-99-9	HMDB0000784	-	-	-	-
M129T213_NEG	Citraconic acid	46183129	7177259	6370198	8875889	9513411	3.549	129.0195	Lipids and lipid-like molecules	Fatty Acyls	Fatty acids and conjugates	498-24-8	HMDB0000749	C02226	ko01100//Metabolic pathways;ko01210//2-Oxocarboxylic acid metabolism;ko00290//Valine, leucine and isoleucine	Amino acid metabolism;Carbohydrate metabolism;Global and overview maps	Metabolism

														biosynthesis;ko00660//C5-Branched dibasic acid metabolism		
M362T34_NEG	N-palmitoyltaurine	13620818	1.54E+08	1.43E+08	58515146	1.24E+08	0.569	362.23771	Lipids and lipid-like molecules	Fatty Acyls	Fatty amides	83982-06-3	HMDB0240594	-	-	-	-
M313T48_NEG	Progesterone	78886166	7208984	8872214	12991529	11188879	0.806	313.23888	Lipids and lipid-like molecules	Steroids and steroid derivatives	Pregnane steroids	57-83-0	HMDB0001830	C00410	ko01100//Metabolic pathways;ko00140//Steroid hormone biosynthesis;ko04925//Aldosterone synthesis and secretion;ko05200//Pathways in	Cancer: overview;Cancer: specific types;Cell growth and death;Endocrine and metabolic disease;Endocri	Cellular Processes;Drug Development;Human Diseases;Metabolism;Organismal Systems

cancer;ko04913//Ovarian steroidogenesis;ko04917//Prolactin signaling pathway;ko04927//Cortisol synthesis and secretion;ko04934//Cushing syndrome;ko05207//Chemical carcinogenesis - receptor activation;ko05215//Prostate cancer;ko04114//Oocyte meiosis;ko04914//Progesterone-	ne system; Global and overview maps;Lipid metabolism;Target-based classification: Nuclear receptors

															mediated oocyte maturation;ko07225//Glucocorticoid and mineralocorticoid receptor agonists/antagonists;ko07226//Progesterone, androgen and estrogen receptor agonists/antagonists;ko05224//Breast cancer		
M288T243_POS	Cyproheptadine	78032848	4322304	2646358	8509892	6474835	4.054	288.1921	Benzenoids	Benzene and substituted derivatives	Phenylmethylamines	961-71-7	HMDB0240227	-	-	-	-

M156T188_POS	Triflumuron	17681465	1.18E+08	1.19E+08	1.02E+08	1.16E+08	3.135	156.0422	Benzenoids	Benzene and substituted derivatives	N-phenylureas	-	HMDB0259200	-	-	-	-
M321T25_NEG	Dynasore	21661563	2.4E+08	2.28E+08	74241862	1.97E+08	0.419	321.0804	Benzenoids	Naphthalenes	Naphthalene carboxylic acids and derivatives	-	HMDB0251652	-	-	-	-
M430T30_2_POS	(+)-.alpha.-tocopherol	3.3E+08	29647253	16917408	31843197	35402944	0.493	430.3779	--	--	--	--	--	C02477	ko01100//Metabolic pathways;ko01110//Biosynthesis of secondary metabolites;ko01060//Biosynthesis of plant secondary metabolites;ko04977//Vitami	Cell growth and death;Chemical structure transformation maps;Digestive system;Global and overview maps;M	Cellular Processes;Metabolism;Organismal Systems

															n digestion and absorption;ko04216//Ferroptosis;ko00130//Ubiquinone and other terpenoid-quinone biosynthesis	etabolism of cofactors and vitamins	
M429T30_2_POS	(2.beta.,3.alpha.,5.alpha.,16.beta.,17.beta.)-17-acetoxy-3-hydroxy-2-(4-morpholinyl)-16-(1-pyrrolidinyl)androstane	6.77E+08	72628753	49592854	65562666	74749392	0.4996	429.3725	--	--	--	--	--	-	-	-	-
M663T29_1_POS	5-(1,2,4a,5-tetramethyl-7-oxo-3,4,8,8a-	4.25E	1.46E	1.48E	1.7E+	1.65E	0.479	663.4	--	--	--	--	--	-	-	-	-

	tetrahydro-2h-naphthalen-1-yl)-3-methylpentanoic acid	+08	+08	+08	08	+08		534										
M501T202_POS	6-[(6,8-dihydroxy-7-methoxy-3-methyl-1-oxo-3,4-dihydroisochromen-4-yl)oxy]-4,8-dihydroxy-7-methoxy-3-methyl-3,4-dihydroisochromen-1-one	22422245	80141118	79611965	73722632	76815262	3.369	501.0657	--	--	--	--	--	-	-	-	-
M311T206_2_POS	6-ethoxy-3-(4-methoxyphenyl)-4-methylcoumarin	49422248	49389258	55512385	39759478	49308981	3.43	311.1384	--	--	--	--	--	-	-	-	-

M536T28_POS	Cephalochromin	1.82E+08	71769824	77064621	1.06E+08	89318469	0.474	536.165	--	--	--	--	--	-	-	-	-
M489T311_2_POS	Cytidine 5'-diphosphocholine (CDP-choline)	1951448	1.23E+08	1.18E+08	1.06E+08	1.12E+08	5.18	489.1147	--	--	--	--	--	C00307	ko01100//Metabolic pathways;ko00564//Glycerophospholipid metabolism;ko05231//Choline metabolism in cancer	Cancer: overview;Global and overview maps;Lipid metabolism	Human Diseases;Metabolism
M273T189_POS	Eleutherin	2598806	36681025	36406353	31990173	35303685	3.144	273.1211	--	--	--	--	--	-	-	-	-
M377T238_PO	Griseofulvic acid	48	33	34	32	32	3.	37	--	--	--	--	--	-	-	-	-

S		4851.3	899526	281342	677915	615870	96	7.0137									
M298T491_POS	N-3-oxododecanoyl-l-homoserine lactone	9497716	50621702	56260162	48818582	53629623	8.187	298.1874	--	--	--	--	--	-	-	-	-

Table S4 Genes of Co- analysis

Gene_ID	Symbol	F0	F1	F2	F3	F4	Description	KEGG_A_class	KEGG_B_class	Pathway	K_ID	GO Component	GO Function	GO Process	TF_family
ncbi_113124908	ak1	20.763333	6.436	5.657	13.41533	8.829	XP_026153862.1 adenylate kinase isoenzyme 1 [Mastacembelus armatus]	Metabolism;Metabolism;Metabolism	Global and overview maps;Nucleotide metabolism;Metabolism of cofactors and vitamins	ko01100//Metabolic pathways;ko00230//Purine metabolism;ko00730//Thiamine metabolism	K00939;K00939;K00939	GO:0001520//outer dense fiber;GO:0005575//cellular_component;GO:0005622//intracellular anatomical structure;GO:0005737//cytoplasm;GO:0005829//cytosol;GO:0005856//cytoskeleton;GO:0005886//plasma membrane;GO:0005929//cilium;GO:0009338//exodeoxyribonuclease V complex;GO:0009898//cytoplasmic side of plasma	GO:0000166//nucleotide binding;GO:0003674//molecular_function;GO:0003824//catalytic activity;GO:0004017//adenylate kinase activity;GO:0004127//cytidylate kinase activity;GO:0004140//dephospho-CoA kinase activity;GO:0004386//helicase activity;GO:0004550//nucleoside diphosphate kinase activity;GO:0004	GO:0000012//single strand break repair;GO:0006139//nucleobase-containing compound metabolic process;GO:0006163//purine nucleotide metabolic process;GO:0006164//purine nucleotide biosynthetic process;GO:0006165//nucleoside diphosphate phosphorylation;GO:0006172//ADP biosynthetic process;GO:0006	--

membrane;GO:0016020//membrane;GO:0030016//myofibril;GO:0030017//sarcomere;GO:0031514//motile cilium;GO:0036126//sperm flagellum;GO:0042995//cell projection;GO:0043005//neuron projection;GO:0043190//ATP-binding cassette (ABC) transporter complex;GO:0043226//organelle;GO:0043228//non-membrane-bounded organelle;GO:0043229//intracellular organelle;GO:0043232//intracellular non-membrane-bounded organelle;GO:00	798//thymidylate kinase activity;GO:0005488//binding;GO:0005524//ATP binding;GO:0008854//exodeoxyribonuclease V activity;GO:0015413//ABC-type nickel transporter activity;GO:0016301//kinase activity;GO:0016740//transferase activity;GO:0016772//transferase activity, transferring phosphorus-containing groups;GO:0016776//phosphotransferase activity, phosphate group as acceptor;GO:0017076//purine nucleotide binding;GO:0019205//nucleobase-containing	281//DNA repair;GO:0006310//DNA recombination;GO:0006725//cellular aromatic compound metabolic process;GO:0006753//nucleoside phosphate metabolic process;GO:0006793//phosphorus metabolic process;GO:0006796//phosphate-containing compound metabolic process;GO:0006807//nitrogen compound metabolic process;GO:0007049//cell cycle;GO:0008150//biological_process;GO:0008152//metabolic process;GO:0009058//biosynthetic

43292//contractile fiber;GO:0048471//perinuclear region of cytoplasm;GO:0071944//cell periphery;GO:0097729//9+2 motile cilium;GO:0099080//supramolecular complex;GO:0099081//supramolecular polymer;GO:0099512//supramolecular fiber;GO:0099513//polymeric cytoskeletal fiber;GO:0120025//plasma membrane bounded cell projection	compound kinase activity;GO:0030554//adenyl nucleotide binding;GO:0032553//ribonucleotide binding;GO:0032555//purine ribonucleotide binding;GO:0032559//adenyl ribonucleotide binding;GO:0035639//purine ribonucleoside triphosphate binding;GO:0036094//small molecule binding;GO:0043167//ion binding;GO:0043168//anion binding;GO:0046403//polynucleotide 3'-phosphatase activity;GO:0046404//polydeoxyribonucleotide 5'-hydroxyl-kinase	process;GO:0009116//nucleoside metabolic process;GO:0009117//nucleotide metabolic process;GO:0009119//ribonucleoside metabolic process;GO:0009123//nucleoside monophosphate metabolic process;GO:0009124//nucleoside monophosphate biosynthetic process;GO:0009126//purine nucleoside monophosphate metabolic process;GO:0009127//purine nucleoside monophosphate biosynthetic process;GO:0009132//nucleoside diphosphate metabolic process;GO:0009

activity;GO:0050145//nucleoside monophosphate kinase activity;GO:0097159//organic cyclic compound binding;GO:0097367//carbohydrate derivative binding;GO:1901265//nucleoside phosphate binding;GO:1901363//heterocyclic compound binding	133//nucleoside diphosphate biosynthetic process;GO:0009135//purine nucleoside diphosphate metabolic process;GO:0009136//purine nucleoside diphosphate biosynthetic process;GO:0009141//nucleoside triphosphate metabolic process;GO:0009142//nucleoside triphosphate biosynthetic process;GO:0009144//purine nucleoside triphosphate metabolic process;GO:0009150//purine ribonucleotide metabolic process;GO:0009151//purine

deoxyribonucleot
ide metabolic
process;GO:0009
152//purine
ribonucleotide
biosynthetic
process;GO:0009
156//ribonucleosi
de
monophosphate
biosynthetic
process;GO:0009
161//ribonucleosi
de
monophosphate
metabolic
process;GO:0009
163//nucleoside
biosynthetic
process;GO:0009
165//nucleotide
biosynthetic
process;GO:0009
167//purine
ribonucleoside
monophosphate
metabolic
process;GO:0009
168//purine
ribonucleoside
monophosphate
biosynthetic

process;GO:0009
179//purine
ribonucleoside
diphosphate
metabolic
process;GO:0009
180//purine
ribonucleoside
diphosphate
biosynthetic
process;GO:0009
185//ribonucleosi
de diphosphate
metabolic
process;GO:0009
188//ribonucleosi
de diphosphate
biosynthetic
process;GO:0009
199//ribonucleosi
de triphosphate
metabolic
process;GO:0009
205//purine
ribonucleoside
triphosphate
metabolic
process;GO:0009
259//ribonucleoti
de metabolic
process;GO:0009
260//ribonucleoti

de biosynthetic process;GO:0009987//cellular process;GO:0010827//regulation of glucose transmembrane transport;GO:0010828//positive regulation of glucose transmembrane transport;GO:0015675//nickel cation transport;GO:0015937//coenzyme A biosynthetic process;GO:0015949//nucleobase-containing small molecule interconversion;GO:0016310//phosphorylation;GO:0018130//heterocycle biosynthetic process;GO:0019438//aromatic compound biosynthetic

process;GO:0019
637//organophos
phate metabolic
process;GO:0019
693//ribose
phosphate
metabolic
process;GO:0022
402//cell cycle
process;GO:0032
879//regulation
of
localization;GO:
0034404//nucleo
base-containing
small molecule
biosynthetic
process;GO:0034
641//cellular
nitrogen
compound
metabolic
process;GO:0034
654//nucleobase-
containing
compound
biosynthetic
process;GO:0034
762//regulation
of
transmembrane
transport;GO:003

4764//positive
regulation of
transmembrane
transport;GO:004
2278//purine
nucleoside
metabolic
process;GO:0042
451//purine
nucleoside
biosynthetic
process;GO:0042
455//ribonucleosi
de biosynthetic
process;GO:0044
237//cellular
metabolic
process;GO:0044
238//primary
metabolic
process;GO:0044
249//cellular
biosynthetic
process;GO:0044
271//cellular
nitrogen
compound
biosynthetic
process;GO:0044
281//small
molecule
metabolic

process;GO:0044
283//small
molecule
biosynthetic
process;GO:0045
786//negative
regulation of cell
cycle;GO:00460
31//ADP
metabolic
process;GO:0046
033//AMP
metabolic
process;GO:0046
034//ATP
metabolic
process;GO:0046
102//inosine
metabolic
process;GO:0046
103//inosine
biosynthetic
process;GO:0046
128//purine
ribonucleoside
metabolic
process;GO:0046
129//purine
ribonucleoside
biosynthetic
process;GO:0046
390//ribose

phosphate
biosynthetic
process;GO:0046
483//heterocycle
metabolic
process;GO:0046
939//nucleotide
phosphorylation;
GO:0046940//nu
cleoside
monophosphate
phosphorylation;
GO:0048518//po
sitive regulation
of biological
process;GO:0048
519//negative
regulation of
biological
process;GO:0048
523//negative
regulation of
cellular
process;GO:0050
789//regulation
of biological
process;GO:0050
794//regulation
of cellular
process;GO:0051
049//regulation
of

transport;GO:005
1050//positive
regulation of
transport;GO:005
1726//regulation
of cell
cycle;GO:00550
86//nucleobase-
containing small
molecule
metabolic
process;GO:0065
007//biological
regulation;GO:00
71704//organic
substance
metabolic
process;GO:0072
521//purine-
containing
compound
metabolic
process;GO:0072
522//purine-
containing
compound
biosynthetic
process;GO:0090
407//organophos
phate
biosynthetic
process;GO:1901

135//carbohydrat
e derivative
metabolic
process;GO:1901
137//carbohydrat
e derivative
biosynthetic
process;GO:1901
293//nucleoside
phosphate
biosynthetic
process;GO:1901
360//organic
cyclic compound
metabolic
process;GO:1901
362//organic
cyclic compound
biosynthetic
process;GO:1901
564//organonitro
gen compound
metabolic
process;GO:1901
566//organonitro
gen compound
biosynthetic
process;GO:1901
576//organic
substance
biosynthetic
process;GO:1901

													657//glycosyl compound metabolic process;GO:1901659//glycosyl compound biosynthetic process	
ncbi_113128715	Ern1	28.30067	12.07467	11.99567	12.722	12.63967	XP_026160011.1 serine/threonine-protein kinase/endoribonuclease IRE1-like [Mastacembelus arma	Human Diseases;Human Diseases;Human Diseases;Human Diseases;Human Diseases;Human Diseases;Genetic Information Processing;Human Diseases;Cellular Processes;Cellular Processes	Neurodegenerative disease;Neurodegenerative disease;Neurodegenerative disease;Neurodegenerative disease;Neurodegenerative disease;Cardiovascular disease;Endocrine and metabolic disease;Folding, sorting and degradation;Neurodegenerative disease;Transport and catabolism;Cell growth and death	ko05022//Pathways of neurodegeneration - multiple diseases;ko05010//Alzheimer disease;ko05014//Amyotrophic lateral sclerosis;ko05016//Huntington disease;ko05012//Parkinson disease;ko05417//Lipid and atherosclerosis;ko04932//Non-alcoholic fatty liver disease;ko04141//Protein processing in endoplasmic reticulum;ko050	K08852;K08852;K08852;K08852;K08852;K08852;K08852;K08852;K08852	GO:0005575//cellular_component;GO:0005622//intracellular anatomical structure;GO:0005737//cytoplasm;GO:0005783//endoplasmic reticulum;GO:0005789//endoplasmic reticulum membrane;GO:0009279//cell outer membrane;GO:0012505//endomembrane system;GO:0016020//membrane;GO:0016021//integral component of membrane;GO:0	GO:0000166//nucleotide binding;GO:0000287//magnesium ion binding;GO:0003674//molecular_function;GO:0003824//catalytic activity;GO:0004518//nuclease activity;GO:0004519//endonuclease activity;GO:0004540//ribonuclease activity;GO:0004672//protein kinase activity;GO:0004674//protein serine/threonine kinase	GO:0000165//MAPK cascade;GO:0001932//regulation of protein phosphorylation;GO:0001934//positive regulation of protein phosphorylation;GO:0006139//nucleobase-containing compound metabolic process;GO:0006323//DNA packaging;GO:0006351//transcription, DNA-templated;GO:0006355//regulation of transcription,	--

tus]	17//Spinocerebellar ataxia;ko04140//Autophagy - animal;ko04210//Apoptosis	031224//intrinsic component of membrane;GO:0031984//organelle subcompartment;GO:0032991//protein-containing complex;GO:0042175//nuclear outer membrane-endoplasmic reticulum membrane network;GO:0043226//organelle;GO:0043227//membrane-bounded organelle;GO:0043229//intracellular organelle;GO:0043231//intracellular membrane-bounded organelle;GO:0098796//membrane protein complex;GO:0098827//endoplasmic reticulum subcompartment;	activity;GO:0005488//binding;GO:0005515//protein binding;GO:0005524//ATP binding;GO:0016301//kinase activity;GO:0016491//oxidoreductase activity;GO:0016740//transferase activity;GO:0016772//transferase activity, transferring phosphorus-containing groups;GO:0016773//phosphotransferase activity, alcohol group as acceptor;GO:0016787//hydrolase activity;GO:0016788//hydrolase activity, acting on ester bonds;GO:0017076//purine nucleotide	DNA-templated;GO:0006396//RNA processing;GO:0006397//mRNA processing;GO:0006401//RNA catabolic process;GO:0006464//cellular protein modification process;GO:0006468//protein phosphorylation;GO:0006725//cellular aromatic compound metabolic process;GO:0006793//phosphorus metabolic process;GO:0006796//phosphate-containing compound metabolic process;GO:0006807//nitrogen compound metabolic process;GO:0006

GO:1990604//IRE1-TRAF2-ASK1 complex	binding;GO:0030554//adenyl nucleotide binding;GO:0032553//ribonucleotide binding;GO:0032555//purine ribonucleotide binding;GO:0032559//adenyl ribonucleotide binding;GO:0035639//purine ribonucleoside triphosphate binding;GO:0036094//small molecule binding;GO:0043167//ion binding;GO:0043168//anion binding;GO:0043169//cation binding;GO:0046872//metal ion binding;GO:0097159//organic cyclic compound binding;GO:0097367//carbohydrat	915//apoptotic process;GO:0006921//cellular component disassembly involved in execution phase of apoptosis;GO:0006950//response to stress;GO:0006986//response to unfolded protein;GO:0006996//organelle organization;GO:0007049//cell cycle;GO:0007154//cell communication; GO:0007165//signal transduction;GO:0007254//JNK cascade;GO:0008150//biological_process;GO:0008152//metabolic process;GO:0008219//cell death;GO:00090

e derivative binding;GO:0140096//catalytic activity, acting on a protein;GO:0140098//catalytic activity, acting on RNA;GO:1901265//nucleoside phosphate binding;GO:1901363//heterocyclic compound binding	56//catabolic process;GO:0009057//macromolecule catabolic process;GO:0009058//biosynthetic process;GO:0009059//macromolecule biosynthetic process;GO:0009889//regulation of biosynthetic process;GO:0009890//negative regulation of biosynthetic process;GO:0009892//negative regulation of metabolic process;GO:0009893//positive regulation of metabolic process;GO:0009966//regulation of signal transduction;GO:0009967//positive regulation of signal transduction;GO:

0009987//cellular
process;GO:0010
033//response to
organic
substance;GO:00
10467//gene
expression;GO:0
010468//regulati
on of gene
expression;GO:0
010556//regulati
on of
macromolecule
biosynthetic
process;GO:0010
558//negative
regulation of
macromolecule
biosynthetic
process;GO:0010
562//positive
regulation of
phosphorus
metabolic
process;GO:0010
604//positive
regulation of
macromolecule
metabolic
process;GO:0010
605//negative
regulation of

macromolecule metabolic process;GO:0010629//negative regulation of gene expression;GO:0010646//regulation of cell communication;GO:0010647//positive regulation of cell communication;GO:0012501//programmed cell death;GO:0016043//cellular component organization;GO:0016070//RNA metabolic process;GO:0016071//mRNA metabolic process;GO:0016072//rRNA metabolic process;GO:0016075//rRNA catabolic process;GO:0016

310//phosphoryla
tion;GO:0018130
//heterocycle
biosynthetic
process;GO:0019
219//regulation
of nucleobase-
containing
compound
metabolic
process;GO:0019
220//regulation
of phosphate
metabolic
process;GO:0019
222//regulation
of metabolic
process;GO:0019
438//aromatic
compound
biosynthetic
process;GO:0019
439//aromatic
compound
catabolic
process;GO:0019
538//protein
metabolic
process;GO:0022
402//cell cycle
process;GO:0022
411//cellular

component disassembly;GO:0023051//regulation of signaling;GO:0023052//signaling;GO:0023056//positive regulation of signaling;GO:0030261//chromosome condensation;GO:0030262//apoptotic nuclear changes;GO:0030263//apoptotic chromosome condensation;GO:0031098//stress-activated protein kinase signaling cascade;GO:0031323//regulation of cellular metabolic process;GO:0031324//negative regulation of cellular metabolic process;GO:0031

325//positive
regulation of
cellular
metabolic
process;GO:0031
326//regulation
of cellular
biosynthetic
process;GO:0031
327//negative
regulation of
cellular
biosynthetic
process;GO:0031
399//regulation
of protein
modification
process;GO:0031
401//positive
regulation of
protein
modification
process;GO:0032
147//activation
of protein kinase
activity;GO:0032
268//regulation
of cellular
protein metabolic
process;GO:0032
270//positive
regulation of

cellular protein
metabolic
process;GO:0032
774//RNA
biosynthetic
process;GO:0032
872//regulation
of stress-
activated MAPK
cascade;GO:003
2874//positive
regulation of
stress-activated
MAPK
cascade;GO:003
3554//cellular
response to
stress;GO:00336
74//positive
regulation of
kinase
activity;GO:0034
641//cellular
nitrogen
compound
metabolic
process;GO:0034
645//cellular
macromolecule
biosynthetic
process;GO:0034
654//nucleobase-

containing
compound
biosynthetic
process;GO:0034
655//nucleobase-
containing
compound
catabolic
process;GO:0034
660//ncRNA
metabolic
process;GO:0034
661//ncRNA
catabolic
process;GO:0034
976//response to
endoplasmic
reticulum
stress;GO:00355
56//intracellular
signal
transduction;GO:
0035966//respon
se to
topologically
incorrect
protein;GO:0036
211//protein
modification
process;GO:0042
221//response to
chemical;GO:00

42325//regulation of phosphorylation;GO:0042327//positive regulation of phosphorylation;GO:0043085//positive regulation of catalytic activity;GO:0043170//macromolecule metabolic process;GO:0043405//regulation of MAP kinase activity;GO:0043406//positive regulation of MAP kinase activity;GO:0043408//regulation of MAPK cascade;GO:0043410//positive regulation of MAPK cascade;GO:0043412//macromolecule modification;GO:0043506//regula

tion of JUN
kinase
activity;GO:0043
507//positive
regulation of
JUN kinase
activity;GO:0043
549//regulation
of kinase
activity;GO:0044
093//positive
regulation of
molecular
function;GO:004
4237//cellular
metabolic
process;GO:0044
238//primary
metabolic
process;GO:0044
248//cellular
catabolic
process;GO:0044
249//cellular
biosynthetic
process;GO:0044
260//cellular
macromolecule
metabolic
process;GO:0044
265//cellular
macromolecule

catabolic
process;GO:0044
267//cellular
protein metabolic
process;GO:0044
270//cellular
nitrogen
compound
catabolic
process;GO:0044
271//cellular
nitrogen
compound
biosynthetic
process;GO:0045
786//negative
regulation of cell
cycle;GO:00458
59//regulation of
protein kinase
activity;GO:0045
860//positive
regulation of
protein kinase
activity;GO:0045
892//negative
regulation of
transcription,
DNA-
templated;GO:00
45934//negative
regulation of

nucleobase-
containing
compound
metabolic
process;GO:0045
937//positive
regulation of
phosphate
metabolic
process;GO:0046
328//regulation
of JNK
cascade;GO:004
6330//positive
regulation of
JNK
cascade;GO:004
6483//heterocycl
e metabolic
process;GO:0046
700//heterocycle
catabolic
process;GO:0048
518//positive
regulation of
biological
process;GO:0048
519//negative
regulation of
biological
process;GO:0048
522//positive

regulation of
cellular
process;GO:0048
523//negative
regulation of
cellular
process;GO:0048
583//regulation
of response to
stimulus;GO:004
8584//positive
regulation of
response to
stimulus;GO:005
0789//regulation
of biological
process;GO:0050
790//regulation
of catalytic
activity;GO:0050
794//regulation
of cellular
process;GO:0050
896//response to
stimulus;GO:005
1171//regulation
of nitrogen
compound
metabolic
process;GO:0051
172//negative
regulation of

nitrogen
compound
metabolic
process;GO:0051
173//positive
regulation of
nitrogen
compound
metabolic
process;GO:0051
174//regulation
of phosphorus
metabolic
process;GO:0051
246//regulation
of protein
metabolic
process;GO:0051
247//positive
regulation of
protein metabolic
process;GO:0051
252//regulation
of RNA
metabolic
process;GO:0051
253//negative
regulation of
RNA metabolic
process;GO:0051
276//chromosom
e

organization;GO:
0051338//regulat
ion of transferase
activity;GO:0051
347//positive
regulation of
transferase
activity;GO:0051
403//stress-
activated MAPK
cascade;GO:005
1716//cellular
response to
stimulus;GO:005
1726//regulation
of cell
cycle;GO:00602
55//regulation of
macromolecule
metabolic
process;GO:0065
007//biological
regulation;GO:00
65009//regulatio
n of molecular
function;GO:007
0059//intrinsic
apoptotic
signaling
pathway in
response to
endoplasmic

reticulum
stress;GO:00703
02//regulation of
stress-activated
protein kinase
signaling
cascade;GO:007
0304//positive
regulation of
stress-activated
protein kinase
signaling
cascade;GO:007
1103//DNA
conformation
change;GO:0071
704//organic
substance
metabolic
process;GO:0071
840//cellular
component
organization or
biogenesis;GO:0
071900//regulati
on of protein
serine/threonine
kinase
activity;GO:0071
902//positive
regulation of
protein

serine/threonine
kinase
activity;GO:0080
090//regulation
of primary
metabolic
process;GO:0080
134//regulation
of response to
stress;GO:00801
35//regulation of
cellular response
to
stress;GO:00903
04//nucleic acid
metabolic
process;GO:0090
305//nucleic acid
phosphodiester
bond
hydrolysis;GO:0
090501//RNA
phosphodiester
bond
hydrolysis;GO:0
097190//apoptoti
c signaling
pathway;GO:009
7193//intrinsic
apoptotic
signaling
pathway;GO:009

7194//execution
phase of
apoptosis;GO:00
97659//nucleic
acid-templated
transcription;GO:
1901360//organic
cyclic compound
metabolic
process;GO:1901
361//organic
cyclic compound
catabolic
process;GO:1901
362//organic
cyclic compound
biosynthetic
process;GO:1901
564//organonitro
gen compound
metabolic
process;GO:1901
575//organic
substance
catabolic
process;GO:1901
576//organic
substance
biosynthetic
process;GO:1902
531//regulation
of intracellular

signal
transduction;GO:
1902533//positiv
e regulation of
intracellular
signal
transduction;GO:
1902679//negativ
e regulation of
RNA
biosynthetic
process;GO:1903
506//regulation
of nucleic acid-
templated
transcription;GO:
1903507//negativ
e regulation of
nucleic acid-
templated
transcription;GO:
2000112//regulat
ion of cellular
macromolecule
biosynthetic
process;GO:2000
113//negative
regulation of
cellular
macromolecule
biosynthetic
process;GO:2001

ncbi_113134827	WNT9A	2.255	0.134	0.294667	1.767	0.575	XP_026170143.1 protein Wnt-9a [Mastacembelus armatus]	Human Diseases;Human Diseases;Human Diseases;Human Diseases;Human Diseases;Environmental Information Processing;Human Diseases;Human Diseases;Human Diseases;Environmental Information Processing;Environmental Information Processing;Cellular	Cancer: overview;Neurodegenerative disease;Neurodegenerative disease;Infectious disease: viral;Cancer: overview;Cancer: specific types;Signal transduction;Cancer: specific types;Cancer: specific types;Endocrine and metabolic disease;Signal transduction;Signal transduction;Cellular community - eukaryotes;Endocrine system;Cancer: specific types	ko05200//Pathways in cancer;ko05022//Pathways of neurodegeneration - multiple diseases;ko05010//Alzheimer disease;ko05165//Human papillomavirus infection;ko05205//Proteoglycans in cancer;ko05226//Gastric cancer;ko04310//Wnt signaling pathway;ko05225//Hepatocellular carcinoma;ko05224//Breast cancer;ko04934//Cushing syndrome;ko04150//mTOR signaling pathway;ko0439	K01064;K01064;K01064;K01064;K01064;K01064;K01064;K01064;K01064;K01064;K01064;K01064	GO:0005575//cellular_component;GO:0005576//extracellular region;GO:0005615//extracellular space	GO:0001664//G protein-coupled receptor binding;GO:0003674//molecular_function;GO:0005102//signaling receptor binding;GO:0005109//frizzled binding;GO:0005488//binding;GO:0005515//protein binding;GO:0030545//signaling receptor regulator activity;GO:0048018//receptor ligand activity;GO:0098772//molecular function regulator	141//regulation of RNA biosynthetic process GO:0000075//cell cycle checkpoint signaling;GO:0000278//mitotic cell cycle;GO:0001101//response to acid chemical;GO:0001501//skeletal system development;GO:0001654//eye development;GO:0007049//cell cycle;GO:0007093//mitotic cell cycle checkpoint signaling;GO:0007154//cell communication;GO:0007165//signal transduction;GO:0007166//cell surface receptor signaling	--

Processes;Organismal Systems;Human Diseases	0//Hippo signaling pathway;ko04550//Signaling pathways regulating pluripotency of stem cells;ko04916//Melanogenesis;ko05217//Basal cell carcinoma	pathway;GO:0007267//cell-cell signaling;GO:0007275//multicellular organism development;GO:0007346//regulation of mitotic cell cycle;GO:0007399//nervous system development;GO:0007423//sensory organ development;GO:0008150//biological_process;GO:0008285//negative regulation of cell population proliferation;GO:0008589//regulation of smoothened signaling pathway;GO:0009653//anatomical structure morphogenesis;GO:0009790//embryo

development;GO:0009792//embryo development ending in birth or egg hatching;GO:0009887//animal organ morphogenesis;GO:0009892//negative regulation of metabolic process;GO:0009966//regulation of signal transduction;GO:0009967//positive regulation of signal transduction;GO:0009987//cellular process;GO:0010033//response to organic substance;GO:0010466//negative regulation of peptidase activity;GO:0010469//regulation of signaling receptor

activity;GO:0010
605//negative
regulation of
macromolecule
metabolic
process;GO:0010
646//regulation
of cell
communication;
GO:0010647//po
sitive regulation
of cell
communication;
GO:0010941//reg
ulation of cell
death;GO:00109
51//negative
regulation of
endopeptidase
activity;GO:0016
055//Wnt
signaling
pathway;GO:001
9222//regulation
of metabolic
process;GO:0022
008//neurogenesi
s;GO:0022402//c
ell cycle
process;GO:0023
051//regulation
of

signaling;GO:0023052//signaling;GO:0023056//positive regulation of signaling;GO:0030154//cell differentiation;GO:0030162//regulation of proteolysis;GO:0030182//neuron differentiation;GO:0030326//embryonic limb morphogenesis;GO:0031323//regulation of cellular metabolic process;GO:0031324//negative regulation of cellular metabolic process;GO:0032268//regulation of cellular protein metabolic process;GO:0032269//negative regulation of cellular protein

metabolic
process;GO:0032
330//regulation
of chondrocyte
differentiation;G
O:0032331//nega
tive regulation of
chondrocyte
differentiation;G
O:0032501//mult
icellular
organismal
process;GO:0032
502//developmen
tal
process;GO:0032
526//response to
retinoic
acid;GO:003399
3//response to
lipid;GO:003510
7//appendage
morphogenesis;G
O:0035108//limb
morphogenesis;G
O:0035113//emb
ryonic
appendage
morphogenesis;G
O:0035115//emb
ryonic forelimb
morphogenesis;G

O:0035136//forel
imb
morphogenesis;G
O:0040019//posit
ive regulation of
embryonic
development;GO
:0042127//regula
tion of cell
population
proliferation;GO:
0042221//respon
se to
chemical;GO:00
42981//regulatio
n of apoptotic
process;GO:0043
009//chordate
embryonic
development;GO
:0043010//camer
a-type eye
development;GO
:0043066//negati
ve regulation of
apoptotic
process;GO:0043
067//regulation
of programmed
cell
death;GO:00430
69//negative

regulation of
programmed cell
death;GO:00430
86//negative
regulation of
catalytic
activity;GO:0043
154//negative
regulation of
cysteine-type
endopeptidase
activity involved
in apoptotic
process;GO:0043
281//regulation
of cysteine-type
endopeptidase
activity involved
in apoptotic
process;GO:0044
092//negative
regulation of
molecular
function;GO:004
5165//cell fate
commitment;GO:
0045595//regulat
ion of cell
differentiation;G
O:0045596//nega
tive regulation of
cell

differentiation;GO:0045597//positive regulation of cell differentiation;GO:0045786//negative regulation of cell cycle;GO:0045861//negative regulation of proteolysis;GO:0045880//positive regulation of smoothened signaling pathway;GO:0045930//negative regulation of mitotic cell cycle;GO:0045995//regulation of embryonic development;GO:0048513//animal organ development;GO:0048518//positive regulation of biological process;GO:0048519//negative

regulation of
biological
process;GO:0048
522//positive
regulation of
cellular
process;GO:0048
523//negative
regulation of
cellular
process;GO:0048
562//embryonic
organ
morphogenesis;G
O:0048568//emb
ryonic organ
development;GO
:0048583//regula
tion of response
to
stimulus;GO:004
8584//positive
regulation of
response to
stimulus;GO:004
8592//eye
morphogenesis;G
O:0048593//cam
era-type eye
morphogenesis;G
O:0048598//emb
ryonic

morphogenesis;GO:0048699//generation of neurons;GO:0048704//embryonic skeletal system morphogenesis;GO:0048705//skeletal system morphogenesis;GO:0048706//embryonic skeletal system development;GO:0048731//system development;GO:0048736//appendage development;GO:0048856//anatomical structure development;GO:0048869//cellular developmental process;GO:0050789//regulation of biological process;GO:0050790//regulation of catalytic activity;GO:0050

793//regulation
of developmental
process;GO:0050
794//regulation
of cellular
process;GO:0050
896//response to
stimulus;GO:005
1093//negative
regulation of
developmental
process;GO:0051
094//positive
regulation of
developmental
process;GO:0051
171//regulation
of nitrogen
compound
metabolic
process;GO:0051
172//negative
regulation of
nitrogen
compound
metabolic
process;GO:0051
239//regulation
of multicellular
organismal
process;GO:0051
240//positive

regulation of
multicellular
organismal
process;GO:0051
241//negative
regulation of
multicellular
organismal
process;GO:0051
246//regulation
of protein
metabolic
process;GO:0051
248//negative
regulation of
protein metabolic
process;GO:0051
336//regulation
of hydrolase
activity;GO:0051
346//negative
regulation of
hydrolase
activity;GO:0051
716//cellular
response to
stimulus;GO:005
1726//regulation
of cell
cycle;GO:00525
47//regulation of
peptidase

activity;GO:0052
548//regulation
of endopeptidase
activity;GO:0060
070//canonical
Wnt signaling
pathway;GO:006
0173//limb
development;GO
:0060255//regula
tion of
macromolecule
metabolic
process;GO:0060
548//negative
regulation of cell
death;GO:00610
35//regulation of
cartilage
development;GO
:0061037//negati
ve regulation of
cartilage
development;GO
:0061072//iris
morphogenesis;G
O:0061303//corn
ea development
in camera-type
eye;GO:0065007
//biological
regulation;GO:00

65009//regulation of molecular function;GO:0070887//cellular response to chemical stimulus;GO:0071229//cellular response to acid chemical;GO:0071300//cellular response to retinoic acid;GO:0071310//cellular response to organic substance;GO:0071396//cellular response to lipid;GO:0072498//embryonic skeletal joint development;GO:0080090//regulation of primary metabolic process;GO:0090596//sensory organ morphogenesis;GO:0198738//cell-

cell signaling by wnt;GO:1901700//response to oxygen-containing compound;GO:1901701//cellular response to oxygen-containing compound;GO:1902762//regulation of embryonic skeletal joint development;GO:1902764//positive regulation of embryonic skeletal joint development;GO:1903047//mitotic cell cycle process;GO:1905114//cell surface receptor signaling pathway involved in cell-cell signaling;GO:2000026//regulation of multicellular

													organismal development;GO:2000116//regulation of cysteine-type endopeptidase activity;GO:2000117//negative regulation of cysteine-type endopeptidase activity	
ncbi_113132656	Flnc	31.70967	11.60167	8.396	15.86367	12.54567	XP_026166722.1 filamin-C-like isoform X1 [Mastacembelus armatus]	Environmental Information Processing;Human Diseases;Human Diseases;Cellular Processes	Signal transduction;Infectious disease: bacterial;Cancer: overview;Cellular community - eukaryotes	ko04010//MAPK signaling pathway;ko05132//Salmonella infection;ko05205//Proteoglycans in cancer;ko04510//Focal adhesion	K04437;K04437;K04437;K04437	-	GO:0005515//protein binding;GO:0008658//penicillin binding	-	- -
ncb	NMB	19.	1.9	1.7	2.0	2.9	XP_02614743	Environmental Information Processing	Signaling molecules and interaction	ko04080//Neuroactive ligand-receptor	K05223	GO:0005575//cellular_component;GO:0005576//ext	GO:0001664//G protein-coupled receptor	GO:0006810//transport;GO:0006811//ion	- -

i_113121310	10167	77667	09	97333	60333	7.1 neuromedin-B [Mastacembelus armatus]	interaction	racellular region;GO:0042995//cell projection;GO:0043005//neuron projection;GO:0120025//plasma membrane bounded cell projection	binding;GO:0003674//molecular_function;GO:0005102//signaling receptor binding;GO:0005179//hormone activity;GO:0005488//binding;GO:0005515//protein binding;GO:0030545//signaling receptor regulator activity;GO:0031705//bombesin receptor binding;GO:0031710//neuromedin B receptor binding;GO:0048018//receptor ligand activity;GO:0071855//neuropeptide receptor binding;GO:0098772//molecular function regulator	transport;GO:0006820//anion transport;GO:0006869//lipid transport;GO:0006873//cellular ion homeostasis;GO:0006874//cellular calcium ion homeostasis;GO:0006875//cellular metal ion homeostasis;GO:0007154//cell communication;GO:0007165//signal transduction;GO:0007186//G protein-coupled receptor signaling pathway;GO:0007204//positive regulation of cytosolic calcium ion concentration;GO:0007218//neuropeptide signaling

pathway;GO:000
7267//cell-cell
signaling;GO:00
08150//biological
_process;GO:000
8284//positive
regulation of cell
population
proliferation;GO:
0009966//regulat
ion of signal
transduction;GO:
0009987//cellular
process;GO:0010
469//regulation
of signaling
receptor
activity;GO:0010
646//regulation
of cell
communication;
GO:0010647//po
sitive regulation
of cell
communication;
GO:0010648//ne
gative regulation
of cell
communication;
GO:0010817//reg
ulation of
hormone

levels;GO:00108
76//lipid
localization;GO:
0015711//organic
anion
transport;GO:001
5718//monocarbo
xylic acid
transport;GO:001
5849//organic
acid
transport;GO:001
5908//fatty acid
transport;GO:001
5909//long-chain
fatty acid
transport;GO:001
9725//cellular
homeostasis;GO:
0023051//regulat
ion of
signaling;GO:00
23052//signaling;
GO:0023056//po
sitive regulation
of
signaling;GO:00
23057//negative
regulation of
signaling;GO:00
30003//cellular
cation

homeostasis;GO:
0032309//icosan
oid
secretion;GO:00
32879//regulatio
n of
localization;GO:
0033036//macro
molecule
localization;GO:
0033500//carboh
ydrate
homeostasis;GO:
0042127//regulat
ion of cell
population
proliferation;GO:
0042592//homeo
static
process;GO:0042
593//glucose
homeostasis;GO:
0046717//acid
secretion;GO:00
46883//regulatio
n of hormone
secretion;GO:00
46887//positive
regulation of
hormone
secretion;GO:00
46888//negative

regulation of
hormone
secretion;GO:00
46903//secretion;
GO:0046942//car
boxylic acid
transport;GO:004
8518//positive
regulation of
biological
process;GO:0048
519//negative
regulation of
biological
process;GO:0048
522//positive
regulation of
cellular
process;GO:0048
523//negative
regulation of
cellular
process;GO:0048
583//regulation
of response to
stimulus;GO:004
8878//chemical
homeostasis;GO:
0050482//arachid
onic acid
secretion;GO:00
50789//regulatio

n of biological process;GO:0050794//regulation of cellular process;GO:0050801//ion homeostasis;GO:0050896//response to stimulus;GO:0051046//regulation of secretion;GO:0051047//positive regulation of secretion;GO:0051048//negative regulation of secretion;GO:0051049//regulation of transport;GO:0051050//positive regulation of transport;GO:0051051//negative regulation of transport;GO:0051179//localization;GO:0051234//establishment of localization;GO:

0051480//regulat
ion of cytosolic
calcium ion
concentration;G
O:0051716//cellu
lar response to
stimulus;GO:005
5065//metal ion
homeostasis;GO:
0055074//calciu
m ion
homeostasis;GO:
0055080//cation
homeostasis;GO:
0055082//cellular
chemical
homeostasis;GO:
0065007//biologi
cal
regulation;GO:00
65008//regulatio
n of biological
quality;GO:0065
009//regulation
of molecular
function;GO:007
1702//organic
substance
transport;GO:007
1715//icosanoid
transport;GO:007
2503//cellular

														divalent inorganic cation homeostasis;GO:0072507//divalent inorganic cation homeostasis;GO:0098771//inorganic ion homeostasis;GO:1901571//fatty acid derivative transport;GO:1903530//regulation of secretion by cell;GO:1903531//negative regulation of secretion by cell;GO:1903532//positive regulation of secretion by cell;GO:1903963//arachidonate transport	
ncbi_11	LUM	0.733366	0.090333	0.0337	0.062333	0	XP_026163211.1 lumican-like	Environmental Information Processing	Signal transduction	ko04350//TGF-beta signaling pathway	K08121	-	GO:0005515//protein binding	-	- -

3130643		7	3		3		[Mastacembelus armatus]								
ncbi_113126122	LMNA	8.251	1.154333	0.813	1.214	1.25	XP_026155575.1 lamin-A-like isoform X1 [Mastacembelus armatus]	Cellular Processes;Human Diseases;Human Diseases;Human Diseases	Cell growth and death;Cardiovascular disease;Cardiovascular disease;Cardiovascular disease	ko04210//Apoptosis;ko05414//Dilated cardiomyopathy;ko05410//Hypertrophic cardiomyopathy;ko05412//Arrhythmogenic right ventricular cardiomyopathy	K12641;K12641;K12641;K12641	GO:0005575//cellular_component;GO:0005622//intracellular anatomical structure;GO:0005634//nucleus;GO:0005635//nuclear envelope;GO:0005638//lamin filament;GO:0005652//nuclear lamina;GO:0005654//nucleoplasm;GO:0005737//cytoplasm;GO:0005829//cytosol;GO:0005856//cytoskeleton;GO:0005882//intermediate filament;GO:0012505//endomembrane	GO:0003674//molecular_function;GO:0005198//structural molecule activity;GO:0005488//binding;GO:0005515//protein binding;GO:0005524//ATP binding;GO:0008157//protein phosphatase 1 binding;GO:0019899//enzyme binding;GO:0019902//phosphatase binding;GO:0019903//protein phosphatase binding;GO:0042802//identical protein binding	GO:0000003//reproduction;GO:0000226//microtubule cytoskeleton organization;GO:0000278//mitotic cell cycle;GO:0001666//response to hypoxia;GO:0006606//protein import into nucleus;GO:0006810//transport;GO:0006886//intracellular protein transport;GO:0006913//nucleocytoplasmic transport;GO:0006950//response to stress;GO:0006986//response to unfolded	--

system;GO:0016
020//membrane;
GO:0016363//nu
clear
matrix;GO:0016
604//nuclear
body;GO:001660
7//nuclear
speck;GO:00310
90//organelle
membrane;GO:0
031965//nuclear
membrane;GO:0
031967//organell
e
envelope;GO:00
31974//membran
e-enclosed
lumen;GO:00319
75//envelope;GO
:0031981//nuclea
r
lumen;GO:00343
99//nuclear
periphery;GO:00
43226//organelle;
GO:0043227//me
mbrane-bounded
organelle;GO:00
43228//non-
membrane-
bounded

protein;GO:0006
996//organelle
organization;GO:
0006997//nucleu
s
organization;GO:
0006998//nuclear
envelope
organization;GO:
0007010//cytosk
eleton
organization;GO:
0007017//microt
ubule-based
process;GO:0007
049//cell
cycle;GO:00070
59//chromosome
segregation;GO:
0007084//mitotic
nuclear
membrane
reassembly;GO:0
007154//cell
communication;
GO:0007163//est
ablishment or
maintenance of
cell
polarity;GO:000
7165//signal
transduction;GO:

organelle;GO:0043229//intracellular organelle;GO:0043231//intracellular membrane-bounded organelle;GO:0043232//intracellular non-membrane-bounded organelle;GO:0043233//organelle lumen;GO:0045111//intermediate filament cytoskeleton;GO:0048471//perinuclear region of cytoplasm;GO:0070013//intracellular organelle lumen;GO:0099080//supramolecular complex;GO:0099081//supramolecular polymer;GO:0099512//supramolecular

0007275//multicellular organism development;GO:0007276//gamete generation;GO:0007283//spermatogenesis;GO:0007507//heart development;GO:0007517//muscle organ development;GO:0007568//aging;GO:0007569//cell aging;GO:0008104//protein localization;GO:0008150//biological_process;GO:0008285//negative regulation of cell population proliferation;GO:0009605//response to external stimulus;GO:0009612//response to mechanical stimulus;GO:0009628//response

fiber;GO:0099513//polymeric cytoskeletal fiber	to abiotic stimulus;GO:0009888//tissue development;GO:0009893//positive regulation of metabolic process;GO:0009966//regulation of signal transduction;GO:0009968//negative regulation of signal transduction;GO:0009987//cellular process;GO:0010033//response to organic substance;GO:0010256//endomembrane system organization;GO:0010464//regulation of mesenchymal cell proliferation;GO:0010468//regulation of gene expression;GO:0010604//positive

regulation of
macromolecule
metabolic
process;GO:0010
611//regulation
of cardiac muscle
hypertrophy;GO:
0010612//regulat
ion of cardiac
muscle
adaptation;GO:0
010614//negative
regulation of
cardiac muscle
hypertrophy;GO:
0010616//negativ
e regulation of
cardiac muscle
adaptation;GO:0
010628//positive
regulation of
gene
expression;GO:0
010639//negative
regulation of
organelle
organization;GO:
0010646//regulat
ion of cell
communication;
GO:0010648//ne
gative regulation

of cell
communication;
GO:0010821//reg
ulation of
mitochondrion
organization;GO:
0010823//negativ
e regulation of
mitochondrion
organization;GO:
0010941//regulat
ion of cell
death;GO:00147
06//striated
muscle tissue
development;GO
:0014741//negati
ve regulation of
muscle
hypertrophy;GO:
0014743//regulat
ion of muscle
hypertrophy;GO:
0014745//negativ
e regulation of
muscle
adaptation;GO:0
015031//protein
transport;GO:001
5833//peptide
transport;GO:001
6043//cellular

component organization;GO:0017038//protein import;GO:0019219//regulation of nucleobase-containing compound metabolic process;GO:0019222//regulation of metabolic process;GO:0019953//sexual reproduction;GO:0022402//cell cycle process;GO:0022414//reproductive process;GO:0023051//regulation of signaling;GO:0023052//signaling;GO:0023057//negative regulation of signaling;GO:0030010//establishment of cell polarity;GO:003

0154//cell
differentiation;G
O:0030261//chro
mosome
condensation;GO
:0030278//regula
tion of
ossification;GO:
0030334//regulat
ion of cell
migration;GO:00
30951//establish
ment or
maintenance of
microtubule
cytoskeleton
polarity;GO:003
0952//establishm
ent or
maintenance of
cytoskeleton
polarity;GO:003
0968//endoplasm
ic reticulum
unfolded protein
response;GO:003
1323//regulation
of cellular
metabolic
process;GO:0031
468//nuclear
membrane

reassembly;GO:0032204//regulation of telomere maintenance;GO:0032501//multicellular organismal process;GO:0032502//developmental process;GO:0032504//multicellular organism reproduction;GO:0032879//regulation of localization;GO:0032880//regulation of protein localization;GO:0033036//macromolecule localization;GO:0033043//regulation of organelle organization;GO:0033044//regulation of chromosome organization;GO:0033365//protein localization to organelle;GO:00

33554//cellular response to stress;GO:0034504//protein localization to nucleus;GO:0034613//cellular protein localization;GO:0034620//cellular response to unfolded protein;GO:0034976//response to endoplasmic reticulum stress;GO:0035051//cardiocyte differentiation;GO:0035966//response to topologically incorrect protein;GO:0035967//cellular response to topologically incorrect protein;GO:0036293//response to decreased oxygen

levels;GO:0036294//cellular response to decreased oxygen levels;GO:0036498//IRE1-mediated unfolded protein response;GO:0040012//regulation of locomotion;GO:0042127//regulation of cell population proliferation;GO:0042221//response to chemical;GO:0042692//muscle cell differentiation;GO:0042886//amide transport;GO:0042981//regulation of apoptotic process;GO:0043066//negative regulation of apoptotic

process;GO:0043067//regulation of programmed cell death;GO:0043069//negative regulation of programmed cell death;GO:0043502//regulation of muscle adaptation;GO:0044057//regulation of system process;GO:0044703//multi-organism reproductive process;GO:0045184//establishment of protein localization;GO:0045595//regulation of cell differentiation;GO:0045597//positive regulation of cell differentiation;GO:0045667//regulation of osteoblast

differentiation;GO:0045669//positive regulation of osteoblast differentiation;GO:0045778//positive regulation of ossification;GO:0046907//intracellular transport;GO:0048232//male gamete generation;GO:0048468//cell development;GO:0048513//animal organ development;GO:0048518//positive regulation of biological process;GO:0048519//negative regulation of biological process;GO:0048522//positive regulation of cellular process;GO:0048523//negative

regulation of
cellular
process;GO:0048
583//regulation
of response to
stimulus;GO:004
8585//negative
regulation of
response to
stimulus;GO:004
8609//multicellul
ar organismal
reproductive
process;GO:0048
731//system
development;GO
:0048738//cardia
c muscle tissue
development;GO
:0048856//anato
mical structure
development;GO
:0048869//cellula
r developmental
process;GO:0050
789//regulation
of biological
process;GO:0050
793//regulation
of developmental
process;GO:0050
794//regulation

of cellular
process;GO:0050
896//response to
stimulus;GO:005
1052//regulation
of DNA
metabolic
process;GO:0051
093//negative
regulation of
developmental
process;GO:0051
094//positive
regulation of
developmental
process;GO:0051
128//regulation
of cellular
component
organization;GO:
0051129//negativ
e regulation of
cellular
component
organization;GO:
0051146//striated
muscle cell
differentiation;G
O:0051169//nucl
ear
transport;GO:005
1170//import into

nucleus;GO:0051
171//regulation
of nitrogen
compound
metabolic
process;GO:0051
179//localization;
GO:0051234//est
ablishment of
localization;GO:
0051239//regulat
ion of
multicellular
organismal
process;GO:0051
240//positive
regulation of
multicellular
organismal
process;GO:0051
241//negative
regulation of
multicellular
organismal
process;GO:0051
270//regulation
of cellular
component
movement;GO:0
051641//cellular
localization;GO:
0051649//establis

hment of
localization in
cell;GO:0051704
//multi-organism
process;GO:0051
716//cellular
response to
stimulus;GO:005
5001//muscle
cell
development;GO
:0055002//striate
d muscle cell
development;GO
:0055006//cardia
c cell
development;GO
:0055007//cardia
c muscle cell
differentiation;G
O:0055012//vent
ricular cardiac
muscle cell
differentiation;G
O:0055013//cardi
ac muscle cell
development;GO
:0055015//ventri
cular cardiac
muscle cell
development;GO
:0060255//regula

tion of
macromolecule
metabolic
process;GO:0060
341//regulation
of cellular
localization;GO:
0060537//muscle
tissue
development;GO
:0060548//negati
ve regulation of
cell
death;GO:00610
24//membrane
organization;GO:
0061061//muscle
structure
development;GO
:0065007//biolog
ical
regulation;GO:00
65008//regulatio
n of biological
quality;GO:0070
482//response to
oxygen
levels;GO:00707
27//cellular
macromolecule
localization;GO:
0070887//cellular

response to
chemical
stimulus;GO:007
1310//cellular
response to
organic
substance;GO:00
71453//cellular
response to
oxygen
levels;GO:00714
56//cellular
response to
hypoxia;GO:007
1702//organic
substance
transport;GO:007
1705//nitrogen
compound
transport;GO:007
1840//cellular
component
organization or
biogenesis;GO:0
072201//negative
regulation of
mesenchymal
cell
proliferation;GO:
0072359//circulat
ory system
development;GO

:0072594//establi
shment of protein
localization to
organelle;GO:00
80090//regulatio
n of primary
metabolic
process;GO:0080
134//regulation
of response to
stress;GO:00901
99//regulation of
release of
cytochrome c
from
mitochondria;GO
:0090201//negati
ve regulation of
release of
cytochrome c
from
mitochondria;GO
:0090257//regula
tion of muscle
system
process;GO:0090
342//regulation
of cell
aging;GO:00903
43//positive
regulation of cell
aging;GO:00903

98//cellular
senescence;GO:1
900180//regulati
on of protein
localization to
nucleus;GO:1903
047//mitotic cell
cycle
process;GO:1903
242//regulation
of cardiac muscle
hypertrophy in
response to
stress;GO:19032
43//negative
regulation of
cardiac muscle
hypertrophy in
response to
stress;GO:19038
27//regulation of
cellular protein
localization;GO:
1904177//regulat
ion of adipose
tissue
development;GO
:1904178//negati
ve regulation of
adipose tissue
development;GO
:2000026//regula

														tion of multicellular organismal development;GO:2000145//regulation of cell motility;GO:2001233//regulation of apoptotic signaling pathway;GO:2001234//negative regulation of apoptotic signaling pathway;GO:2001236//regulation of extrinsic apoptotic signaling pathway;GO:2001237//negative regulation of extrinsic apoptotic signaling pathway	
ncbi_	ANO1	2.975	0.782	0.858	1.456	1.079	XP_026147449.1 anoc	-	-	-	-	GO:0016021//integral component of membrane	GO:0032051//clathrin light chain binding;GO:0032977//membrane insertase	GO:0007212//dopamine receptor signaling pathway;GO:0048268//clathrin	- -

113121320			667		667	667	tamin-1-like isoform X1 [Mastacembelus armatus]						activity;GO:0046983//protein dimerization activity	coat assembly	
ncbi_113122354	Fxyd3	86.483	28.40433	33.11933	67.34133	39.55433	XP_026149432.1 FXYD domain-containing ion transport regulator 3-like [Mastacembel	-	-	-	-	GO:0005575//cellular_component;GO:0005622//intracellular anatomical structure;GO:0005737//cytoplasm;GO:0005783//endoplasmic reticulum;GO:0005789//endoplasmic reticulum membrane;GO:0005886//plasma membrane;GO:0005887//integral component of plasma membrane;GO:0012505//endome	GO:0003674//molecular_function;GO:0005215//transporter activity;GO:0005216//ion channel activity;GO:0005253//anion channel activity;GO:0005254//chloride channel activity;GO:0005488//binding;GO:0005515//protein binding;GO:0008509//anion transmembrane transporter	GO:0002028//regulation of sodium ion transport;GO:0006810//transport;GO:0006811//ion transport;GO:0006812//cation transport;GO:0006813//potassium ion transport;GO:0006814//sodium ion transport;GO:0006820//anion transport;GO:0006821//chloride transport;GO:0008150//biological_process;GO:0010	- -

us<br>arma<br>tus]	mbrane<br>system;GO:0016<br>020//membrane;<br>GO:0016021//int<br>egral component<br>of<br>membrane;GO:0<br>031224//intrinsic<br>component of<br>membrane;GO:0<br>031226//intrinsic<br>component of<br>plasma<br>membrane;GO:0<br>031984//organell<br>e<br>subcompartment;<br>GO:0042175//nu<br>clear outer<br>membrane-<br>endoplasmic<br>reticulum<br>membrane<br>network;GO:004<br>3226//organelle;<br>GO:0043227//me<br>mbrane-bounded<br>organelle;GO:00<br>43229//intracellu<br>lar<br>organelle;GO:00<br>43231//intracellu	activity;GO:0015<br>075//ion<br>transmembrane<br>transporter<br>activity;GO:0015<br>103//inorganic<br>anion<br>transmembrane<br>transporter<br>activity;GO:0015<br>108//chloride<br>transmembrane<br>transporter<br>activity;GO:0015<br>267//channel<br>activity;GO:0015<br>318//inorganic<br>molecular entity<br>transmembrane<br>transporter<br>activity;GO:0016<br>247//channel<br>regulator<br>activity;GO:0017<br>080//sodium<br>channel regulator<br>activity;GO:0019<br>899//enzyme<br>binding;GO:0022<br>803//passive<br>transmembrane<br>transporter	959//regulation<br>of metal ion<br>transport;GO:001<br>5698//inorganic<br>anion<br>transport;GO:002<br>2898//regulation<br>of<br>transmembrane<br>transporter<br>activity;GO:0030<br>001//metal ion<br>transport;GO:003<br>2409//regulation<br>of transporter<br>activity;GO:0032<br>412//regulation<br>of ion<br>transmembrane<br>transporter<br>activity;GO:0032<br>879//regulation<br>of<br>localization;GO:<br>0034220//ion<br>transmembrane<br>transport;GO:003<br>4762//regulation<br>of<br>transmembrane<br>transport;GO:003<br>4765//regulation

			lar membrane-bounded organelle;GO:0071944//cell periphery;GO:0098827//endoplasmic reticulum subcompartment	activity;GO:0022857//transmembrane transporter activity;GO:0051117//ATPase binding;GO:0098772//molecular function regulator;GO:0099106//ion channel regulator activity	of ion transmembrane transport;GO:0043269//regulation of ion transport;GO:0050789//regulation of biological process;GO:0050790//regulation of catalytic activity;GO:0051049//regulation of transport;GO:0051179//localization;GO:0051234//establishment of localization;GO:0055085//transmembrane transport;GO:0065007//biological regulation;GO:0065009//regulation of molecular function;GO:0098656//anion transmembrane transport;GO:0098660//inorganic ion

														transmembrane transport;GO:0098661//inorganic anion transmembrane transport;GO:1902305//regulation of sodium ion transmembrane transport;GO:1902476//chloride transmembrane transport;GO:1904062//regulation of cation transmembrane transport;GO:2000649//regulation of sodium ion transmembrane transporter activity	
ncbi_113312	MAP3K19	5.309	1.008333	1.468	1.433	1.192	XP_026150283.1 mitogen-activated protein	-	-	-	-	GO:0005575//cellular_component;GO:0005622//intracellular anatomical structure;GO:0005737//cytoplasm	GO:0000166//nucleotide binding;GO:0003674//molecular_function;GO:0003824//catalytic activity;GO:0004672//protein kinase activity;GO:0004	GO:0001932//regulation of protein phosphorylation;GO:0001934//positive regulation of protein phosphorylation;GO:0006464//cellular protein modification	- -

2868	kinase kinase kinase 19 isoform X1 [Mastacembelus armatus]	674//protein serine/threonine kinase activity;GO:0005488//binding;GO:0005515//protein binding;GO:0005524//ATP binding;GO:0016301//kinase activity;GO:0016740//transferase activity;GO:0016772//transferase activity, transferring phosphorus-containing groups;GO:0016773//phosphotransferase activity, alcohol group as acceptor;GO:0017076//purine nucleotide binding;GO:0030554//adenyl nucleotide binding;GO:0032553//ribonucleotide	process;GO:0006468//protein phosphorylation;GO:0006793//phosphorus metabolic process;GO:0006796//phosphate-containing compound metabolic process;GO:0006807//nitrogen compound metabolic process;GO:0006950//response to stress;GO:0007154//cell communication;GO:0007165//signal transduction;GO:0007346//regulation of mitotic cell cycle;GO:0008150//biological_process;GO:0008152//metabolic process;GO:0009893//positive

binding;GO:0032<br>555//purine<br>ribonucleotide<br>binding;GO:0032<br>559//adenyl<br>ribonucleotide<br>binding;GO:0035<br>639//purine<br>ribonucleoside<br>triphosphate<br>binding;GO:0036<br>094//small<br>molecule<br>binding;GO:0043<br>167//ion<br>binding;GO:0043<br>168//anion<br>binding;GO:0097<br>159//organic<br>cyclic compound<br>binding;GO:0097<br>367//carbohydrat<br>e derivative<br>binding;GO:0140<br>096//catalytic<br>activity, acting<br>on a<br>protein;GO:1901<br>265//nucleoside<br>phosphate<br>binding;GO:1901<br>363//heterocyclic	regulation of<br>metabolic<br>process;GO:0009<br>987//cellular<br>process;GO:0010<br>562//positive<br>regulation of<br>phosphorus<br>metabolic<br>process;GO:0010<br>604//positive<br>regulation of<br>macromolecule<br>metabolic<br>process;GO:0010<br>941//regulation<br>of cell<br>death;GO:00163<br>10//phosphorylati<br>on;GO:0019220//<br>regulation of<br>phosphate<br>metabolic<br>process;GO:0019<br>222//regulation<br>of metabolic<br>process;GO:0019<br>538//protein<br>metabolic<br>process;GO:0023<br>052//signaling;G<br>O:0031098//stres

compound binding	s-activated protein kinase signaling cascade;GO:0031323//regulation of cellular metabolic process;GO:0031325//positive regulation of cellular metabolic process;GO:0031399//regulation of protein modification process;GO:0031401//positive regulation of protein modification process;GO:0032147//activation of protein kinase activity;GO:0032268//regulation of cellular protein metabolic process;GO:0032270//positive regulation of cellular protein

metabolic
process;GO:0033
554//cellular
response to
stress;GO:00336
74//positive
regulation of
kinase
activity;GO:0035
556//intracellular
signal
transduction;GO:
0036211//protein
modification
process;GO:0042
325//regulation
of
phosphorylation;
GO:0042327//po
sitive regulation
of
phosphorylation;
GO:0042981//reg
ulation of
apoptotic
process;GO:0043
067//regulation
of programmed
cell
death;GO:00430
85//positive
regulation of

catalytic
activity;GO:0043
170//macromolec
ule metabolic
process;GO:0043
412//macromolec
ule
modification;GO
:0043549//regula
tion of kinase
activity;GO:0044
093//positive
regulation of
molecular
function;GO:004
4237//cellular
metabolic
process;GO:0044
238//primary
metabolic
process;GO:0044
260//cellular
macromolecule
metabolic
process;GO:0044
267//cellular
protein metabolic
process;GO:0045
859//regulation
of protein kinase
activity;GO:0045
860//positive

regulation of
protein kinase
activity;GO:0045
937//positive
regulation of
phosphate
metabolic
process;GO:0048
518//positive
regulation of
biological
process;GO:0048
522//positive
regulation of
cellular
process;GO:0050
789//regulation
of biological
process;GO:0050
790//regulation
of catalytic
activity;GO:0050
794//regulation
of cellular
process;GO:0050
896//response to
stimulus;GO:005
1171//regulation
of nitrogen
compound
metabolic
process;GO:0051

173//positive
regulation of
nitrogen
compound
metabolic
process;GO:0051
174//regulation
of phosphorus
metabolic
process;GO:0051
246//regulation
of protein
metabolic
process;GO:0051
247//positive
regulation of
protein metabolic
process;GO:0051
338//regulation
of transferase
activity;GO:0051
347//positive
regulation of
transferase
activity;GO:0051
716//cellular
response to
stimulus;GO:005
1726//regulation
of cell
cycle;GO:00602
55//regulation of

														macromolecule metabolic process;GO:0065007//biological regulation;GO:0065009//regulation of molecular function;GO:0071704//organic substance metabolic process;GO:0080090//regulation of primary metabolic process;GO:1901564//organonitrogen compound metabolic process	
ncbi_1131238	GJA5	2.769	0.774	0.502333	0.860333	0.634	XP_0261520 39.1 gap junction alpha-5 protein [Mas	-	-	-	-	-	-	-	- -

94							tacembelus armatus]							
ncbi_113124620	ZNF532	0.777333	0.26	0.334333	0.432667	0.334667	XP_026153429.1 zinc finger protein 532 isoform X1 [Mastacembelus armatus]	-	-	-	-	GO:0005575//cellular_component;GO:0005622//intracellular anatomical structure;GO:0005634//nucleus;GO:0043226//organelle;GO:0043227//membrane-bounded organelle;GO:0043229//intracellular organelle;GO:0043231//intracellular membrane-bounded organelle	GO:0000981//DNA-binding transcription factor activity, RNA polymerase II-specific;GO:0003674//molecular_function;GO:0003676//nucleic acid binding;GO:0003677//DNA binding;GO:0003700//DNA-binding transcription factor activity;GO:0005488//binding;GO:0043167//ion binding;GO:0043169//cation binding;GO:0046872//metal ion binding;GO:0097159//organic	GO:0006139//nucleobase-containing compound metabolic process;GO:0006351//transcription, DNA-templated;GO:0006355//regulation of transcription, DNA-templated;GO:0006357//regulation of transcription by RNA polymerase II;GO:0006725//cellular aromatic compound metabolic process;GO:0006807//nitrogen compound metabolic process;GO:0008	zf-C2H2

cyclic compound binding;GO:0140110//transcription regulator activity;GO:1901363//heterocyclic compound binding	150//biological_process;GO:0008152//metabolic process;GO:0009058//biosynthetic process;GO:0009059//macromolecule biosynthetic process;GO:0009889//regulation of biosynthetic process;GO:0009987//cellular process;GO:0010467//gene expression;GO:0010468//regulation of gene expression;GO:0010556//regulation of macromolecule biosynthetic process;GO:0016070//RNA metabolic process;GO:0018130//heterocycle biosynthetic process;GO:0019219//regulation of nucleobase-

containing
compound
metabolic
process;GO:0019
222//regulation
of metabolic
process;GO:0019
438//aromatic
compound
biosynthetic
process;GO:0031
323//regulation
of cellular
metabolic
process;GO:0031
326//regulation
of cellular
biosynthetic
process;GO:0032
774//RNA
biosynthetic
process;GO:0034
641//cellular
nitrogen
compound
metabolic
process;GO:0034
645//cellular
macromolecule
biosynthetic
process;GO:0034
654//nucleobase-

containing
compound
biosynthetic
process;GO:0043
170//macromolec
ule metabolic
process;GO:0044
237//cellular
metabolic
process;GO:0044
238//primary
metabolic
process;GO:0044
249//cellular
biosynthetic
process;GO:0044
260//cellular
macromolecule
metabolic
process;GO:0044
271//cellular
nitrogen
compound
biosynthetic
process;GO:0046
483//heterocycle
metabolic
process;GO:0050
789//regulation
of biological
process;GO:0050
794//regulation

of cellular
process;GO:0051
171//regulation
of nitrogen
compound
metabolic
process;GO:0051
252//regulation
of RNA
metabolic
process;GO:0060
255//regulation
of
macromolecule
metabolic
process;GO:0065
007//biological
regulation;GO:00
71704//organic
substance
metabolic
process;GO:0080
090//regulation
of primary
metabolic
process;GO:0090
304//nucleic acid
metabolic
process;GO:0097
659//nucleic
acid-templated
transcription;GO:

														1901360//organic cyclic compound metabolic process;GO:1901362//organic cyclic compound biosynthetic process;GO:1901576//organic substance biosynthetic process;GO:1903506//regulation of nucleic acid-templated transcription;GO:2000112//regulation of cellular macromolecule biosynthetic process;GO:2001141//regulation of RNA biosynthetic process	
ncbi_11	MYO7A	14.01933	0.64133	1.63933	4.83066	2.78933	XP_02615427 2.1 LOWQU	-	-	-	-	GO:0000323//lytic vacuole;GO:0001750//photoreceptor outer segment;GO:0001917//photorecep	GO:0000146//microfilament motor activity;GO:0000166//nucleotide binding;GO:0003674//molecular_f	GO:0000902//cell morphogenesis;GO:0000904//cell morphogenesis involved in differentiation;G	- -

3125164	3	3	3	7	3	ALITY PROTEIN: unconventional myosin-VIIa [Mastacembelus armatus]		tor inner segment;GO:0002139//stereocilia coupling link;GO:0002140//stereocilia tip link;GO:0005575//cellular_component;GO:0005622//intracellular anatomical structure;GO:0005737//cytoplasm;GO:0005764//lysosome;GO:0005765//lysosomal membrane;GO:0005773//vacuole;GO:0005774//vacuolar membrane;GO:0005829//cytosol;GO:0005856//cytoskeleton;GO:0005886//plasma membrane;GO:0005902//microvillus;GO:0005929//cilium;GO:0005938//cell cortex;GO:0009898//cytoplasmic	unction;GO:0003774//cytoskeletal motor activity;GO:0003777//microtubule motor activity;GO:0003779//actin binding;GO:0003824//catalytic activity;GO:0005215//transporter activity;GO:0005488//binding;GO:0005515//protein binding;GO:0005516//calmodulin binding;GO:0005524//ATP binding;GO:0008017//microtubule binding;GO:0008092//cytoskeletal protein binding;GO:0015631//tubulin binding;GO:0016462//pyrophosphatase activity;GO:0016787//hydrolase	O:0001654//eye development;GO:0001754//eye photoreceptor cell differentiation;GO:0001845//phagolysosome assembly;GO:0002064//epithelial cell development;GO:0002065//columnar/cuboidal epithelial cell differentiation;GO:0002066//columnar/cuboidal epithelial cell development;GO:0002093//auditory receptor cell morphogenesis;GO:0003008//system process;GO:0006810//transport;GO:0006886//intracellular protein transport;GO:0006897//endocytosis;GO:0006909//p

side of plasma membrane;GO:0015629//actin cytoskeleton;GO:0016020//membrane;GO:0016324//apical plasma membrane;GO:0016459//myosin complex;GO:0016461//unconventional myosin complex;GO:0031090//organelle membrane;GO:0031237//intrinsic component of periplasmic side of plasma membrane;GO:0031410//cytoplasmic vesicle;GO:0031477//myosin VII complex;GO:0031982//vesicle;GO:0032391//photoreceptor connecting cilium;GO:0032420//stereocilium;GO:0032421//ste	activity;GO:0016817//hydrolase activity, acting on acid anhydrides;GO:0016818//hydrolase activity, acting on acid anhydrides, in phosphorus-containing anhydrides;GO:0016887//ATP hydrolysis activity;GO:0017076//purine nucleotide binding;GO:0017111//nucleoside-triphosphatase activity;GO:0019904//protein domain specific binding;GO:0030507//spectrin binding;GO:0030554//adenyl nucleotide binding;GO:0032553//ribonucleotide binding;GO:0032	hagocytosis;GO:0006928//movement of cell or subcellular component;GO:0006996//organelle organization;GO:0007017//microtubule-based process;GO:0007018//microtubule-based movement;GO:0007033//vacuole organization;GO:0007040//lysosome organization;GO:0007275//multicellular organism development;GO:0007399//nervous system development;GO:0007423//sensory organ development;GO:0007600//sensory perception;GO:0007601//visual

reocilium bundle;GO:0032991//protein-containing complex;GO:0035869//ciliary transition zone;GO:0042470//melanosome;GO:0042995//cell projection;GO:0043005//neuron projection;GO:0043226//organelle;GO:0043227//membrane-bounded organelle;GO:0043228//non-membrane-bounded organelle;GO:0043229//intracellular organelle;GO:0043231//intracellular membrane-bounded organelle;GO:0043232//intracellular non-membrane-	555//purine ribonucleotide binding;GO:0032559//adenyl ribonucleotide binding;GO:0035639//purine ribonucleoside triphosphate binding;GO:0036094//small molecule binding;GO:0042802//identical protein binding;GO:0042803//protein homodimerization activity;GO:0043167//ion binding;GO:0043168//anion binding;GO:0043531//ADP binding;GO:0044877//protein-containing complex binding;GO:0046983//protein dimerization	perception;GO:0007605//sensory perception of sound;GO:0008104//protein localization;GO:0008150//biological_process;GO:0008544//epidermis development;GO:0009653//anatomical structure morphogenesis;GO:0009790//embryo development;GO:0009791//post-embryonic development;GO:0009886//post-embryonic animal morphogenesis;GO:0009887//animal organ morphogenesis;GO:0009888//tissue development;GO:0009913//epidermal cell

bounded organelle;GO:0045177//apical part of cell;GO:0045202//synapse;GO:0048770//pigment granule;GO:0055052//ATP-binding cassette (ABC) transporter complex, substrate-binding subunit-containing;GO:0071944//cell periphery;GO:0097708//intracellular vesicle;GO:0097730//non-motile cilium;GO:0097731//9+0 non-motile cilium;GO:0097733//photoreceptor cell cilium;GO:0098588//bounding membrane of organelle;GO:00	activity;GO:0047485//protein N-terminus binding;GO:0048502//ABC-type thiamine transporter activity;GO:0051015//actin filament binding;GO:0097159//organic cyclic compound binding;GO:0097367//carbohydrate derivative binding;GO:1901265//nucleoside phosphate binding;GO:1901363//heterocyclic compound binding	differentiation;GO:0009987//cellular process;GO:0015031//protein transport;GO:0015833//peptide transport;GO:0015888//thiamine transport;GO:0016043//cellular component organization;GO:0016050//vesicle organization;GO:0016192//vesicle-mediated transport;GO:0022008//neurogenesis;GO:0022607//cellular component assembly;GO:0030029//actin filament-based process;GO:0030030//cell projection organization;GO:0030048//actin filament-based movement;GO:0

98590//plasma membrane region;GO:0098852//lytic vacuole membrane;GO:0098858//actin-based cell projection;GO:0098862//cluster of actin-based cell projections;GO:0099568//cytoplasmic region;GO:0120025//plasma membrane bounded cell projection;GO:1990427//stereocilia tip-link density;GO:1990435//upper tip-link density	030154//cell differentiation;GO:0030182//neuron differentiation;GO:0030855//epithelial cell differentiation;GO:0030974//thiamine pyrophosphate transmembrane transport;GO:0031175//neuron projection development;GO:0032501//multicellular organismal process;GO:0032502//developmental process;GO:0032989//cellular component morphogenesis;GO:0033036//macromolecule localization;GO:0033059//cellular pigmentation;GO:0034613//cellula

r protein localization;GO:0035315//hair cell differentiation;GO:0042461//photoreceptor cell development;GO:0042462//eye photoreceptor cell development;GO:0042471//ear morphogenesis;GO:0042472//inner ear morphogenesis;GO:0042490//mechanoreceptor differentiation;GO:0042491//inner ear auditory receptor cell differentiation;GO:0042886//amide transport;GO:0043473//pigmentation;GO:0043583//ear development;GO:0044085//cellula

r component
biogenesis;GO:0
045184//establish
ment of protein
localization;GO:
0046530//photor
eceptor cell
differentiation;G
O:0046907//intra
cellular
transport;GO:004
8468//cell
development;GO
:0048513//animal
organ
development;GO
:0048562//embry
onic organ
morphogenesis;G
O:0048563//post-
embryonic
animal organ
morphogenesis;G
O:0048568//emb
ryonic organ
development;GO
:0048569//post-
embryonic
animal organ
development;GO
:0048592//eye
morphogenesis;G

O:0048598//emb
ryonic
morphogenesis;G
O:0048666//neur
on
development;GO
:0048667//cell
morphogenesis
involved in
neuron
differentiation;G
O:0048699//gene
ration of
neurons;GO:004
8731//system
development;GO
:0048839//inner
ear
development;GO
:0048856//anato
mical structure
development;GO
:0048869//cellula
r developmental
process;GO:0050
877//nervous
system
process;GO:0050
885//neuromuscu
lar process
controlling
balance;GO:0050

905//neuromuscu
lar
process;GO:0050
953//sensory
perception of
light
stimulus;GO:005
0954//sensory
perception of
mechanical
stimulus;GO:005
0957//equilibrioc
eption;GO:00511
79//localization;
GO:0051234//est
ablishment of
localization;GO:
0051640//organel
le
localization;GO:
0051641//cellular
localization;GO:
0051648//vesicle
localization;GO:
0051649//establis
hment of
localization in
cell;GO:0051650
//establishment
of vesicle
localization;GO:
0051656//establis

hment of
organelle
localization;GO:
0051875//pigmen
t granule
localization;GO:
0051904//pigmen
t granule
transport;GO:005
1905//establishm
ent of pigment
granule
localization;GO:
0060088//auditor
y receptor cell
stereocilium
organization;GO:
0060113//inner
ear receptor cell
differentiation;G
O:0060117//audit
ory receptor cell
development;GO
:0060119//inner
ear receptor cell
development;GO
:0060122//inner
ear receptor cell
stereocilium
organization;GO:
0060429//epitheli
um

development;GO
:0060563//neuroe
pithelial cell
differentiation;G
O:0070727//cellu
lar
macromolecule
localization;GO:
0070925//organel
le
assembly;GO:00
71702//organic
substance
transport;GO:007
1705//nitrogen
compound
transport;GO:007
1840//cellular
component
organization or
biogenesis;GO:0
080171//lytic
vacuole
organization;GO:
0090382//phagos
ome
maturation;GO:0
090596//sensory
organ
morphogenesis;G
O:0098657//imp
ort into

														cell;GO:0120036 //plasma membrane bounded cell projection organization	
ncbi_113126286	FCGBP	775.2133	0.564	32.255	99.78167	63.87467	XP_026156038.1 IgG Fc-binding protein-like, partial [Mastacembelus armatus]	-	-	-	-	-	-	-	- -
ncbi_11	FCGBP	1152.67	0.82766	47.107	135.342	85.2266	XP_026156413.1 IgG Fc-bindi	-	-	-	-	-	-	-	- -

3126552		3	7		3	7	ng protein-like [Mastacembelus armatus]								
ncbi_113128205	ADGRL3	1.991333	0.262	0.288333	0.474	0.377667	XP_026159130.1 adhesion G protein-coupled receptor L3-like isoform X6 [Mastacembelus armatus]	-	-	-	-	GO:0016020//membrane;GO:0016021//integral component of membrane	GO:0004930//G protein-coupled receptor activity;GO:0030246//carbohydrate binding	GO:0007186//G protein-coupled receptor signaling pathway	--

							armatus]								
ncbi_113128539	hand2	15.771667	4.325333	4.464667	5.743333	4.745	XP_026159737.1 heart- and neural crest derivatives-expressed protein 2 [Mastacembelus armatus]	-	-	-	-	GO:0000228//nuclear chromosome;GO:0000785//chromatin;GO:0005575//cellular_component;GO:0005622//intracellular anatomical structure;GO:0005634//nucleus;GO:0005654//nucleoplasm;GO:0005667//transcription regulator complex;GO:0005694//chromosome;GO:0031974//membrane-enclosed lumen;GO:0031981//nuclear lumen;GO:0032991//protein-containing complex;GO:0043226//organelle;GO:0043227//membrane-bounded organelle;GO:00	GO:0000976//transcription cis-regulatory region binding;GO:0000977//RNA polymerase II transcription regulatory region sequence-specific DNA binding;GO:0000978//RNA polymerase II cis-regulatory region sequence-specific DNA binding;GO:0000981//DNA-binding transcription factor activity, RNA polymerase II-specific;GO:0000987//cis-regulatory region sequence-specific DNA binding;GO:0001067//transcriptio	GO:0000902//cell morphogenesis;GO:0000904//cell morphogenesis involved in differentiation;GO:0001101//response to acid chemical;GO:0001501//skeletal system development;GO:0001525//angiogenesis;GO:0001568//blood vessel development;GO:0001667//ameboidal-type cell migration;GO:0001701//in utero embryonic development;GO:0001738//morphogenesis of a polarized epithelium;GO:0001755//neural crest cell migration;GO:00	bHLH

43228//non-membrane-bounded organelle;GO:0043229//intracellular organelle;GO:0043231//intracellular membrane-bounded organelle;GO:0043232//intracellular non-membrane-bounded organelle;GO:0043233//organelle lumen;GO:0070013//intracellular organelle lumen	n regulatory region nucleic acid binding;GO:0001228//DNA-binding transcription activator activity, RNA polymerase II-specific;GO:0003674//molecular_function;GO:0003676//nucleic acid binding;GO:0003677//DNA binding;GO:0003680//minor groove of adenine-thymine-rich DNA binding;GO:0003690//double-stranded DNA binding;GO:0003700//DNA-binding transcription factor activity;GO:0003712//transcriptio	01838//embryonic epithelial tube formation;GO:0001932//regulation of protein phosphorylation;GO:0001934//positive regulation of protein phosphorylation;GO:0001944//vasculature development;GO:0001947//heart looping;GO:0001967//suckling behavior;GO:0002009//morphogenesis of an epithelium;GO:0002164//larval development;GO:0002376//immune system process;GO:0002520//immune system development;GO:0003007//heart morphogenesis;GO:0003008//system

n coregulator activity;GO:0003713//transcription coactivator activity;GO:0005488//binding;GO:0005515//protein binding;GO:0008134//transcription factor binding;GO:0042802//identical protein binding;GO:0042803//protein homodimerization activity;GO:0043565//sequence-specific DNA binding;GO:0046982//protein heterodimerization activity;GO:0046983//protein dimerization activity;GO:0070888//E-box binding;GO:0097159//organic	process;GO:0003013//circulatory system process;GO:0003015//heart process;GO:0003142//cardiogenic plate morphogenesis;GO:0003143//embryonic heart tube morphogenesis;GO:0003144//embryonic heart tube formation;GO:0003151//outflow tract morphogenesis;GO:0003156//regulation of animal organ formation;GO:0003205//cardiac chamber development;GO:0003206//cardiac chamber morphogenesis;GO:0003207//cardiac chamber formation;GO:0003208//cardiac

cyclic compound binding;GO:0140110//transcription regulator activity;GO:1901363//heterocyclic compound binding;GO:1990837//sequence-specific double-stranded DNA binding	ventricle morphogenesis;GO:0003211//cardiac ventricle formation;GO:0003215//cardiac right ventricle morphogenesis;GO:0003219//cardiac right ventricle formation;GO:0003231//cardiac ventricle development;GO:0003253//cardiac neural crest cell migration involved in outflow tract morphogenesis;GO:0003264//regulation of cardioblast proliferation;GO:0003266//regulation of secondary heart field cardioblast proliferation;GO:0003278//apoptotic process involved in heart

morphogenesis;GO:0003342//proepicardium development;GO:0003343//septum transversum development;GO:0003357//noradrenergic neuron differentiation;GO:0006139//nucleobase-containing compound metabolic process;GO:0006351//transcription, DNA-templated;GO:0006355//regulation of transcription, DNA-templated;GO:0006357//regulation of transcription by RNA polymerase II;GO:0006366//transcription by RNA polymerase II;GO:0006584//

catecholamine
metabolic
process;GO:0006
725//cellular
aromatic
compound
metabolic
process;GO:0006
807//nitrogen
compound
metabolic
process;GO:0006
915//apoptotic
process;GO:0006
928//movement
of cell or
subcellular
component;GO:0
007275//multicel
lular organism
development;GO
:0007368//deter
mination of
left/right
symmetry;GO:00
07389//pattern
specification
process;GO:0007
399//nervous
system
development;GO
:0007422//periph

eral nervous
system
development;GO
:0007423//sensor
y organ
development;GO
:0007507//heart
development;GO
:0007508//larval
heart
development;GO
:0007512//adult
heart
development;GO
:0007610//behavi
or;GO:0007631//
feeding
behavior;GO:000
8015//blood
circulation;GO:0
008150//biologic
al_process;GO:0
008152//metaboli
c
process;GO:0008
219//cell
death;GO:00082
83//cell
population
proliferation;GO:
0009058//biosynt
hetic

process;GO:0009
059//macromolec
ule biosynthetic
process;GO:0009
653//anatomical
structure
morphogenesis;G
O:0009712//cate
chol-containing
compound
metabolic
process;GO:0009
713//catechol-
containing
compound
biosynthetic
process;GO:0009
790//embryo
development;GO
:0009791//post-
embryonic
development;GO
:0009792//embry
o development
ending in birth or
egg
hatching;GO:000
9799//specificati
on of
symmetry;GO:00
09855//determina
tion of bilateral

symmetry;GO:0009887//animal organ morphogenesis;GO:0009888//tissue development;GO:0009889//regulation of biosynthetic process;GO:0009891//positive regulation of biosynthetic process;GO:0009892//negative regulation of metabolic process;GO:0009893//positive regulation of metabolic process;GO:0009966//regulation of signal transduction;GO:0009967//positive regulation of signal transduction;GO:0009987//cellular process;GO:0010

002//cardioblast differentiation;GO:0010033//response to organic substance;GO:0010463//mesenchymal cell proliferation;GO:0010467//gene expression;GO:0010468//regulation of gene expression;GO:0010556//regulation of macromolecule biosynthetic process;GO:0010557//positive regulation of macromolecule biosynthetic process;GO:0010562//positive regulation of phosphorus metabolic process;GO:0010604//positive regulation of macromolecule metabolic

process;GO:0010605//negative regulation of macromolecule metabolic process;GO:0010611//regulation of cardiac muscle hypertrophy;GO:0010613//positive regulation of cardiac muscle hypertrophy;GO:0010628//positive regulation of gene expression;GO:0010629//negative regulation of gene expression;GO:0010646//regulation of cell communication;GO:0010647//positive regulation of cell communication;GO:0010656//negative regulation of muscle cell apoptotic

process;GO:0010
660//regulation
of muscle cell
apoptotic
process;GO:0010
662//regulation
of striated
muscle cell
apoptotic
process;GO:0010
664//negative
regulation of
striated muscle
cell apoptotic
process;GO:0010
665//regulation
of cardiac muscle
cell apoptotic
process;GO:0010
667//negative
regulation of
cardiac muscle
cell apoptotic
process;GO:0010
941//regulation
of cell
death;GO:00125
01//programmed
cell
death;GO:00140
31//mesenchymal
cell

development;GO
:0014032//neural
crest cell
development;GO
:0014033//neural
crest cell
differentiation;G
O:0014070//resp
onse to organic
cyclic
compound;GO:0
014706//striated
muscle tissue
development;GO
:0014742//positiv
e regulation of
muscle
hypertrophy;GO:
0014743//regulat
ion of muscle
hypertrophy;GO:
0016043//cellular
component
organization;GO:
0016070//RNA
metabolic
process;GO:0016
331//morphogene
sis of embryonic
epithelium;GO:0
016358//dendrite
development;GO

:0016477//cell
migration;GO:00
18130//heterocyc
le biosynthetic
process;GO:0018
958//phenol-
containing
compound
metabolic
process;GO:0019
219//regulation
of nucleobase-
containing
compound
metabolic
process;GO:0019
220//regulation
of phosphate
metabolic
process;GO:0019
222//regulation
of metabolic
process;GO:0019
438//aromatic
compound
biosynthetic
process;GO:0022
008//neurogenesi
s;GO:0022603//r
egulation of
anatomical
structure

morphogenesis;GO:0023051//regulation of signaling;GO:0023056//positive regulation of signaling;GO:0030030//cell projection organization;GO:0030097//hemopoiesis;GO:0030154//cell differentiation;GO:0030182//neuron differentiation;GO:0030278//regulation of ossification;GO:0030279//negative regulation of ossification;GO:0030326//embryonic limb morphogenesis;GO:0030855//epithelial cell differentiation;GO:0030859//polarized epithelial cell

differentiation;GO:0030878//thyroid gland development;GO:0031175//neuron projection development;GO:0031323//regulation of cellular metabolic process;GO:0031325//positive regulation of cellular metabolic process;GO:0031326//regulation of cellular biosynthetic process;GO:0031328//positive regulation of cellular biosynthetic process;GO:0031399//regulation of protein modification process;GO:0031401//positive regulation of protein

modification
process;GO:0032
268//regulation
of cellular
protein metabolic
process;GO:0032
270//positive
regulation of
cellular protein
metabolic
process;GO:0032
501//multicellula
r organismal
process;GO:0032
502//developmen
tal
process;GO:0032
526//response to
retinoic
acid;GO:003277
4//RNA
biosynthetic
process;GO:0032
784//regulation
of DNA-
templated
transcription,
elongation;GO:0
032786//positive
regulation of
DNA-templated
transcription,

elongation;GO:0
032872//regulati
on of stress-
activated MAPK
cascade;GO:003
2874//positive
regulation of
stress-activated
MAPK
cascade;GO:003
2968//positive
regulation of
transcription
elongation from
RNA polymerase
II
promoter;GO:00
32989//cellular
component
morphogenesis;G
O:0032990//cell
part
morphogenesis;G
O:0033333//fin
development;GO
:0033334//fin
morphogenesis;G
O:0033339//pect
oral fin
development;GO
:0033993//respon
se to

lipid;GO:0034103//regulation of tissue remodeling;GO:0034243//regulation of transcription elongation from RNA polymerase II promoter;GO:0034641//cellular nitrogen compound metabolic process;GO:0034645//cellular macromolecule biosynthetic process;GO:0034654//nucleobase-containing compound biosynthetic process;GO:0035050//embryonic heart tube development;GO:0035051//cardiocyte differentiation;GO:0035107//appe

ndage
morphogenesis;G
O:0035108//limb
morphogenesis;G
O:0035113//emb
ryonic
appendage
morphogenesis;G
O:0035118//emb
ryonic pectoral
fin
morphogenesis;G
O:0035138//pect
oral fin
morphogenesis;G
O:0035148//tube
formation;GO:00
35239//tube
morphogenesis;G
O:0035270//endo
crine system
development;GO
:0035295//tube
development;GO
:0040011//locom
otion;GO:004212
7//regulation of
cell population
proliferation;GO:
0042221//respon
se to
chemical;GO:00

42325//regulation of phosphorylation;GO:0042327//positive regulation of phosphorylation;GO:0042415//norepinephrine metabolic process;GO:0042421//norepinephrine biosynthetic process;GO:0042423//catecholamine biosynthetic process;GO:0042475//odontogenesis of dentin-containing tooth;GO:0042476//odontogenesis;GO:0042692//muscle cell differentiation;GO:0042733//embryonic digit morphogenesis;GO:0042981//regulation of apoptotic process;GO:0043

009//chordate
embryonic
development;GO
:0043066//negati
ve regulation of
apoptotic
process;GO:0043
067//regulation
of programmed
cell
death;GO:00430
69//negative
regulation of
programmed cell
death;GO:00431
70//macromolecu
le metabolic
process;GO:0043
388//positive
regulation of
DNA
binding;GO:0043
392//negative
regulation of
DNA
binding;GO:0043
408//regulation
of MAPK
cascade;GO:004
3410//positive
regulation of
MAPK

cascade;GO:004
3433//negative
regulation of
DNA-binding
transcription
factor
activity;GO:0043
502//regulation
of muscle
adaptation;GO:0
043586//tongue
development;GO
:0044057//regula
tion of system
process;GO:0044
092//negative
regulation of
molecular
function;GO:004
4093//positive
regulation of
molecular
function;GO:004
4237//cellular
metabolic
process;GO:0044
238//primary
metabolic
process;GO:0044
249//cellular
biosynthetic
process;GO:0044

260//cellular macromolecule metabolic process;GO:0044271//cellular nitrogen compound biosynthetic process;GO:0045595//regulation of cell differentiation;GO:0045596//negative regulation of cell differentiation;GO:0045667//regulation of osteoblast differentiation;GO:0045668//negative regulation of osteoblast differentiation;GO:0045893//positive regulation of transcription, DNA-templated;GO:0045935//positive regulation of nucleobase-

containing
compound
metabolic
process;GO:0045
937//positive
regulation of
phosphate
metabolic
process;GO:0045
944//positive
regulation of
transcription by
RNA polymerase
II;GO:0046189//
phenol-
containing
compound
biosynthetic
process;GO:0046
483//heterocycle
metabolic
process;GO:0048
468//cell
development;GO
:0048483//autono
mic nervous
system
development;GO
:0048485//sympa
thetic nervous
system
development;GO

:0048513//animal
organ
development;GO
:0048514//blood
vessel
morphogenesis;G
O:0048518//posit
ive regulation of
biological
process;GO:0048
519//negative
regulation of
biological
process;GO:0048
522//positive
regulation of
cellular
process;GO:0048
523//negative
regulation of
cellular
process;GO:0048
534//hematopoiet
ic or lymphoid
organ
development;GO
:0048538//thymu
s
development;GO
:0048562//embry
onic organ
morphogenesis;G

O:0048565//dige
stive tract
development;GO
:0048568//embry
onic organ
development;GO
:0048569//post-
embryonic
animal organ
development;GO
:0048583//regula
tion of response
to
stimulus;GO:004
8584//positive
regulation of
response to
stimulus;GO:004
8598//embryonic
morphogenesis;G
O:0048646//anat
omical structure
formation
involved in
morphogenesis;G
O:0048666//neur
on
development;GO
:0048667//cell
morphogenesis
involved in
neuron

differentiation;G
O:0048699//gene
ration of
neurons;GO:004
8701//embryonic
cranial skeleton
morphogenesis;G
O:0048703//emb
ryonic
viscerocranium
morphogenesis;G
O:0048704//emb
ryonic skeletal
system
morphogenesis;G
O:0048705//skel
etal system
morphogenesis;G
O:0048706//emb
ryonic skeletal
system
development;GO
:0048729//tissue
morphogenesis;G
O:0048731//syste
m
development;GO
:0048732//gland
development;GO
:0048736//appen
dage
development;GO

:0048738//cardia
c muscle tissue
development;GO
:0048762//mesen
chymal cell
differentiation;G
O:0048812//neur
on projection
morphogenesis;G
O:0048813//dend
rite
morphogenesis;G
O:0048844//arter
y
morphogenesis;G
O:0048856//anat
omical structure
development;GO
:0048858//cell
projection
morphogenesis;G
O:0048863//stem
cell
differentiation;G
O:0048864//stem
cell
development;GO
:0048869//cellula
r developmental
process;GO:0048
870//cell
motility;GO:004

8934//peripheral nervous system neuron differentiation;GO:0048935//peripheral nervous system neuron development;GO:0050789//regulation of biological process;GO:0050793//regulation of developmental process;GO:0050794//regulation of cellular process;GO:0050896//response to stimulus;GO:0051090//regulation of DNA-binding transcription factor activity;GO:0051093//negative regulation of developmental process;GO:0051094//positive regulation of developmental process;GO:0051

098//regulation
of
binding;GO:0051
099//positive
regulation of
binding;GO:0051
100//negative
regulation of
binding;GO:0051
101//regulation
of DNA
binding;GO:0051
146//striated
muscle cell
differentiation;G
O:0051171//regu
lation of nitrogen
compound
metabolic
process;GO:0051
173//positive
regulation of
nitrogen
compound
metabolic
process;GO:0051
174//regulation
of phosphorus
metabolic
process;GO:0051
179//localization;
GO:0051216//car

tilage
development;GO
:0051239//regula
tion of
multicellular
organismal
process;GO:0051
240//positive
regulation of
multicellular
organismal
process;GO:0051
241//negative
regulation of
multicellular
organismal
process;GO:0051
246//regulation
of protein
metabolic
process;GO:0051
247//positive
regulation of
protein metabolic
process;GO:0051
252//regulation
of RNA
metabolic
process;GO:0051
254//positive
regulation of
RNA metabolic

process;GO:0051
674//localization
of
cell;GO:0051704
//multi-organism
process;GO:0051
716//cellular
response to
stimulus;GO:005
5007//cardiac
muscle cell
differentiation;G
O:0055123//dige
stive system
development;GO
:0060021//roof
of mouth
development;GO
:0060039//perica
rdium
development;GO
:0060047//heart
contraction;GO:0
060173//limb
development;GO
:0060255//regula
tion of
macromolecule
metabolic
process;GO:0060
429//epithelium
development;GO

:0060485//mesen
chyme
development;GO
:0060536//cartila
ge
morphogenesis;G
O:0060537//mus
cle tissue
development;GO
:0060548//negati
ve regulation of
cell
death;GO:00605
61//apoptotic
process involved
in
morphogenesis;G
O:0060562//epith
elial tube
morphogenesis;G
O:0060840//arter
y
development;GO
:0060973//cell
migration
involved in heart
development;GO
:0060976//corona
ry vasculature
development;GO
:0060977//corona
ry vasculature

morphogenesis;GO:0060982//coronary artery morphogenesis;GO:0061032//visceral serous pericardium development;GO:0061061//muscle structure development;GO:0061307//cardiac neural crest cell differentiation involved in heart development;GO:0061308//cardiac neural crest cell development involved in heart development;GO:0061309//cardiac neural crest cell development involved in outflow tract morphogenesis;GO:0061323//cell proliferation involved in heart morphogenesis;GO:0061325//cell

proliferation involved in outflow tract morphogenesis;GO:0061371//determination of heart left/right asymmetry;GO:0061448//connective tissue development;GO:0065007//biological regulation;GO:0065009//regulation of molecular function;GO:0070302//regulation of stress-activated protein kinase signaling cascade;GO:0070304//positive regulation of stress-activated protein kinase signaling cascade;GO:0070372//regulation of ERK1 and ERK2 cascade;GO:007

0374//positive regulation of ERK1 and ERK2 cascade;GO:0070887//cellular response to chemical stimulus;GO:0071229//cellular response to acid chemical;GO:0071300//cellular response to retinoic acid;GO:0071310//cellular response to organic substance;GO:0071396//cellular response to lipid;GO:0071407//cellular response to organic cyclic compound;GO:0071704//organic substance metabolic process;GO:0071840//cellular component

organization or
biogenesis;GO:0
071907//determi
nation of
digestive tract
left/right
asymmetry;GO:0
071908//determi
nation of
intestine
left/right
asymmetry;GO:0
072175//epithelia
l tube
formation;GO:00
72359//circulator
y system
development;GO
:0080090//regula
tion of primary
metabolic
process;GO:0080
134//regulation
of response to
stress;GO:00801
35//regulation of
cellular response
to
stress;GO:00902
57//regulation of
muscle system
process;GO:0090

304//nucleic acid
metabolic
process;GO:0097
659//nucleic
acid-templated
transcription;GO:
0120036//plasma
membrane
bounded cell
projection
organization;GO:
0120039//plasma
membrane
bounded cell
projection
morphogenesis;G
O:1900744//regu
lation of
p38MAPK
cascade;GO:190
0745//positive
regulation of
p38MAPK
cascade;GO:190
1360//organic
cyclic compound
metabolic
process;GO:1901
362//organic
cyclic compound
biosynthetic
process;GO:1901

564//organonitro
gen compound
metabolic
process;GO:1901
566//organonitro
gen compound
biosynthetic
process;GO:1901
576//organic
substance
biosynthetic
process;GO:1901
615//organic
hydroxy
compound
metabolic
process;GO:1901
617//organic
hydroxy
compound
biosynthetic
process;GO:1901
700//response to
oxygen-
containing
compound;GO:1
901701//cellular
response to
oxygen-
containing
compound;GO:1
902531//regulati

on of
intracellular
signal
transduction;GO:
1902533//positiv
e regulation of
intracellular
signal
transduction;GO:
1902680//positiv
e regulation of
RNA
biosynthetic
process;GO:1902
742//apoptotic
process involved
in
development;GO
:1903506//regula
tion of nucleic
acid-templated
transcription;GO:
1903508//positiv
e regulation of
nucleic acid-
templated
transcription;GO:
1903929//primar
y palate
development;GO
:1904888//cranial
skeletal system

development;GO
:2000026//regula
tion of
multicellular
organismal
development;GO
:2000027//regula
tion of animal
organ
morphogenesis;G
O:2000112//regu
lation of cellular
macromolecule
biosynthetic
process;GO:2000
136//regulation
of cell
proliferation
involved in heart
morphogenesis;G
O:2000677//regu
lation of
transcription
regulatory region
DNA
binding;GO:2000
679//positive
regulation of
transcription
regulatory region
DNA
binding;GO:2000

763//positive regulation of transcription from RNA polymerase II promoter involved in norepinephrine biosynthetic process;GO:2000764//positive regulation of semaphorin-plexin signaling pathway involved in outflow tract morphogenesis;GO:2000826//regulation of heart morphogenesis;GO:2001141//regulation of RNA biosynthetic process;GO:2001260//regulation of semaphorin-plexin signaling pathway;GO:2001262//positive regulation of semaphorin-

ncbi_113132018	NKX6-2	15.19867	1.236	0.533667	0.718667	1.146	XP_026165598.1 homeobox protein Nkx-6.2 [Mastacembelus armatus]	-	-	-	-	GO:0005575//cellular_component;GO:0005622//intracellular anatomical structure;GO:0005634//nucleus;GO:0043226//organelle;GO:0043227//membrane-bounded organelle;GO:0043229//intracellular organelle;GO:0043231//intracellular membrane-bounded organelle	GO:0000976//transcription cis-regulatory region binding;GO:0000977//RNA polymerase II transcription regulatory region sequence-specific DNA binding;GO:0000978//RNA polymerase II cis-regulatory region sequence-specific DNA binding;GO:0000981//DNA-binding transcription factor activity, RNA polymerase II-specific;GO:0000987//cis-regulatory region sequence-specific DNA binding;GO:0001067//transcriptio	plexin signaling pathway GO:0000122//negative regulation of transcription by RNA polymerase II;GO:0001708//cell fate specification;GO:0003002//regionalization;GO:0003008//system process;GO:0006355//regulation of transcription, DNA-templated;GO:0006357//regulation of transcription by RNA polymerase II;GO:0007272//ensheathment of neurons;GO:0007275//multicellular organism development;GO:0007389//pattern specification process;GO:0007399//nervous	Homeobox

n regulatory region nucleic acid binding;GO:0001227//DNA-binding transcription repressor activity, RNA polymerase II-specific;GO:0003674//molecular_function;GO:0003676//nucleic acid binding;GO:0003677//DNA binding;GO:0003690//double-stranded DNA binding;GO:0003700//DNA-binding transcription factor activity;GO:0004674//protein serine/threonine kinase activity;GO:0005488//binding;GO:0043565//seque	system development;GO:0007417//central nervous system development;GO:0008150//biological_process;GO:0008366//axon ensheathment;GO:0009889//regulation of biosynthetic process;GO:0009890//negative regulation of biosynthetic process;GO:0009892//negative regulation of metabolic process;GO:0009953//dorsal/ventral pattern formation;GO:0009987//cellular process;GO:0010001//glial cell differentiation;GO:0010453//regulation of cell fate commitment;GO:0010454//negativ

nce-specific DNA binding;GO:0097159//organic cyclic compound binding;GO:0140110//transcription regulator activity;GO:1901363//heterocyclic compound binding;GO:1990837//sequence-specific double-stranded DNA binding	e regulation of cell fate commitment;GO:0010455//positive regulation of cell fate commitment;GO:0010468//regulation of gene expression;GO:0010556//regulation of macromolecule biosynthetic process;GO:0010558//negative regulation of macromolecule biosynthetic process;GO:0010605//negative regulation of macromolecule metabolic process;GO:0010629//negative regulation of gene expression;GO:0010720//positive regulation of cell development;GO

:0010721//negative regulation of cell development;GO:0014003//oligodendrocyte development;GO:0014013//regulation of gliogenesis;GO:0014014//negative regulation of gliogenesis;GO:0014015//positive regulation of gliogenesis;GO:0019219//regulation of nucleobase-containing compound metabolic process;GO:0019222//regulation of metabolic process;GO:0021510//spinal cord development;GO:0021511//spinal cord patterning;GO:0021513//spinal

cord
dorsal/ventral
patterning;GO:0021514//ventral
spinal cord
interneuron
differentiation;GO:0021515//cell
differentiation in
spinal
cord;GO:0021517//ventral spinal
cord
development;GO:0021520//spinal
cord motor
neuron cell fate
specification;GO:0021521//ventral spinal cord
interneuron
specification;GO:0021522//spinal
cord motor
neuron
differentiation;GO:0021782//glial
cell
development;GO:0021953//central
nervous system
neuron

differentiation;G
O:0022008//neur
ogenesis;GO:002
2010//central
nervous system
myelination;GO:
0030154//cell
differentiation;G
O:0030182//neur
on
differentiation;G
O:0031016//panc
reas
development;GO
:0031018//endocr
ine pancreas
development;GO
:0031323//regula
tion of cellular
metabolic
process;GO:0031
324//negative
regulation of
cellular
metabolic
process;GO:0031
326//regulation
of cellular
biosynthetic
process;GO:0031
327//negative
regulation of

cellular
biosynthetic
process;GO:0031
641//regulation
of
myelination;GO:
0032291//axon
ensheathment in
central nervous
system;GO:0032
501//multicellula
r organismal
process;GO:0032
502//developmen
tal
process;GO:0035
270//endocrine
system
development;GO
:0042063//glioge
nesis;GO:004255
2//myelination;G
O:0045165//cell
fate
commitment;GO:
0045595//regulat
ion of cell
differentiation;G
O:0045596//nega
tive regulation of
cell
differentiation;G

O:0045597//positive regulation of cell differentiation;GO:0045685//regulation of glial cell differentiation;GO:0045686//negative regulation of glial cell differentiation;GO:0045687//positive regulation of glial cell differentiation;GO:0045892//negative regulation of transcription, DNA-templated;GO:0045934//negative regulation of nucleobase-containing compound metabolic process;GO:0048468//cell development;GO:0048513//animal organ

development;GO:0048518//positive regulation of biological process;GO:0048519//negative regulation of biological process;GO:0048522//positive regulation of cellular process;GO:0048523//negative regulation of cellular process;GO:0048663//neuron fate commitment;GO:0048665//neuron fate specification;GO:0048699//generation of neurons;GO:0048709//oligodendrocyte differentiation;GO:0048731//system development;GO:0048856//anato

mical structure
development;GO
:0048869//cellula
r developmental
process;GO:0050
767//regulation
of
neurogenesis;GO
:0050768//negati
ve regulation of
neurogenesis;GO
:0050769//positiv
e regulation of
neurogenesis;GO
:0050789//regula
tion of biological
process;GO:0050
793//regulation
of developmental
process;GO:0050
794//regulation
of cellular
process;GO:0050
877//nervous
system
process;GO:0050
885//neuromuscu
lar process
controlling
balance;GO:0050
905//neuromuscu
lar

process;GO:0051
093//negative
regulation of
developmental
process;GO:0051
094//positive
regulation of
developmental
process;GO:0051
171//regulation
of nitrogen
compound
metabolic
process;GO:0051
172//negative
regulation of
nitrogen
compound
metabolic
process;GO:0051
239//regulation
of multicellular
organismal
process;GO:0051
240//positive
regulation of
multicellular
organismal
process;GO:0051
241//negative
regulation of
multicellular

organismal
process;GO:0051
252//regulation
of RNA
metabolic
process;GO:0051
253//negative
regulation of
RNA metabolic
process;GO:0051
960//regulation
of nervous
system
development;GO
:0051961//negati
ve regulation of
nervous system
development;GO
:0051962//positiv
e regulation of
nervous system
development;GO
:0060255//regula
tion of
macromolecule
metabolic
process;GO:0060
284//regulation
of cell
development;GO
:0060573//cell
fate specification

involved in pattern specification;GO:0060579//ventral spinal cord interneuron fate commitment;GO:0060581//cell fate commitment involved in pattern specification;GO:0065007//biological regulation;GO:0080090//regulation of primary metabolic process;GO:1902679//negative regulation of RNA biosynthetic process;GO:1903506//regulation of nucleic acid-templated transcription;GO:1903507//negative regulation of nucleic acid-templated

														transcription;GO:2000026//regulation of multicellular organismal development;GO:2000112//regulation of cellular macromolecule biosynthetic process;GO:2000113//negative regulation of cellular macromolecule biosynthetic process;GO:2001141//regulation of RNA biosynthetic process	
ncbi_1131132	Six2	4.20667	0.08	0.106333	0.160667	0.368333	XP_02616602 4.1 homeobox protein SIX2	-	-	-	-	GO:0005575//cellular_component;GO:0005622//intracellular anatomical structure;GO:0005634//nucleus;GO:0043226//organelle;GO:0043227//membrane-bounded	GO:0000976//transcription cis-regulatory region binding;GO:0000977//RNA polymerase II transcription regulatory region sequence-specific DNA binding;GO:0000	GO:0001501//skeletal system development;GO:0001655//urogenital system development;GO:0001656//metanephros development;GO:0001704//formation of primary	Homeobox

2<br>5<br>8	[Mas<br>tace<br>mbel<br>us<br>arma<br>tus]		organelle;GO:00<br>43229//intracellu<br>lar<br>organelle;GO:00<br>43231//intracellu<br>lar membrane-<br>bounded<br>organelle	978//RNA<br>polymerase II<br>cis-regulatory<br>region sequence-<br>specific DNA<br>binding;GO:0000<br>981//DNA-<br>binding<br>transcription<br>factor activity,<br>RNA polymerase<br>II-<br>specific;GO:000<br>0987//cis-<br>regulatory region<br>sequence-<br>specific DNA<br>binding;GO:0001<br>067//transcriptio<br>n regulatory<br>region nucleic<br>acid<br>binding;GO:0001<br>228//DNA-<br>binding<br>transcription<br>activator activity,<br>RNA polymerase<br>II-<br>specific;GO:000<br>3674//molecular_<br>function;GO:000	germ<br>layer;GO:000170<br>7//mesoderm<br>formation;GO:00<br>01708//cell fate<br>specification;GO<br>:0001710//mesod<br>ermal cell fate<br>commitment;GO:<br>0001822//kidney<br>development;GO<br>:0002009//morph<br>ogenesis of an<br>epithelium;GO:0<br>002062//chondro<br>cyte<br>differentiation;G<br>O:0003002//regi<br>onalization;GO:0<br>003337//mesench<br>ymal to epithelial<br>transition<br>involved in<br>metanephros<br>morphogenesis;G<br>O:0003338//meta<br>nephros<br>morphogenesis;G<br>O:0006139//nucl<br>eobase-<br>containing<br>compound

3676//nucleic acid binding;GO:0003677//DNA binding;GO:0003690//double-stranded DNA binding;GO:0003700//DNA-binding transcription factor activity;GO:0005488//binding;GO:0005515//protein binding;GO:0008134//transcription factor binding;GO:0043565//sequence-specific DNA binding;GO:0044877//protein-containing complex binding;GO:0097159//organic cyclic compound binding;GO:0140110//transcription regulator	metabolic process;GO:0006351//transcription, DNA-templated;GO:0006355//regulation of transcription, DNA-templated;GO:0006357//regulation of transcription by RNA polymerase II;GO:0006366//transcription by RNA polymerase II;GO:0006606//protein import into nucleus;GO:0006725//cellular aromatic compound metabolic process;GO:0006807//nitrogen compound metabolic process;GO:0006810//transport;GO:0006886//intra

activity;GO:1901363//heterocyclic compound binding;GO:1990837//sequence-specific double-stranded DNA binding	cellular protein transport;GO:0006913//nucleocytoplasmic transport;GO:0006928//movement of cell or subcellular component;GO:0007275//multicellular organism development;GO:0007369//gastrulation;GO:0007389//pattern specification process;GO:0007423//sensory organ development;GO:0007498//mesoderm development;GO:0007501//mesodermal cell fate specification;GO:0008104//protein localization;GO:0008150//biological_process;GO:0008152//metabo

lic
process;GO:0008
283//cell
population
proliferation;GO:
0008284//positiv
e regulation of
cell population
proliferation;GO:
0009058//biosynt
hetic
process;GO:0009
059//macromolec
ule biosynthetic
process;GO:0009
653//anatomical
structure
morphogenesis;G
O:0009790//emb
ryo
development;GO
:0009792//embry
o development
ending in birth or
egg
hatching;GO:000
9798//axis
specification;GO
:0009887//animal
organ
morphogenesis;G
O:0009888//tissu

e
development;GO
:0009889//regula
tion of
biosynthetic
process;GO:0009
891//positive
regulation of
biosynthetic
process;GO:0009
893//positive
regulation of
metabolic
process;GO:0009
948//anterior/pos
terior axis
specification;GO
:0009952//anteri
or/posterior
pattern
specification;GO
:0009987//cellula
r
process;GO:0010
463//mesenchym
al cell
proliferation;GO:
0010467//gene
expression;GO:0
010468//regulati
on of gene
expression;GO:0

010556//regulati
on of
macromolecule
biosynthetic
process;GO:0010
557//positive
regulation of
macromolecule
biosynthetic
process;GO:0010
604//positive
regulation of
macromolecule
metabolic
process;GO:0010
628//positive
regulation of
gene
expression;GO:0
015031//protein
transport;GO:001
5833//peptide
transport;GO:001
6070//RNA
metabolic
process;GO:0016
477//cell
migration;GO:00
17038//protein
import;GO:0018
130//heterocycle
biosynthetic

process;GO:0019219//regulation of nucleobase-containing compound metabolic process;GO:0019222//regulation of metabolic process;GO:0019438//aromatic compound biosynthetic process;GO:0019827//stem cell population maintenance;GO:0022603//regulation of anatomical structure morphogenesis;GO:0030154//cell differentiation;GO:0030278//regulation of ossification;GO:0030855//epithelial cell differentiation;GO:0031323//regulation of cellular metabolic

process;GO:0031325//positive regulation of cellular metabolic process;GO:0031326//regulation of cellular biosynthetic process;GO:0031328//positive regulation of cellular biosynthetic process;GO:0032330//regulation of chondrocyte differentiation;GO:0032501//multicellular organismal process;GO:0032502//developmental process;GO:0032774//RNA biosynthetic process;GO:0033036//macromolecule localization;GO:0033365//protein

localization to
organelle;GO:00
34504//protein
localization to
nucleus;GO:0034
613//cellular
protein
localization;GO:
0034641//cellular
nitrogen
compound
metabolic
process;GO:0034
645//cellular
macromolecule
biosynthetic
process;GO:0034
654//nucleobase-
containing
compound
biosynthetic
process;GO:0035
019//somatic
stem cell
population
maintenance;GO:
0035239//tube
morphogenesis;G
O:0035295//tube
development;GO
:0035850//epithel
ial cell

differentiation
involved in
kidney
development;GO
:0039666//virion
attachment to
host cell
pilus;GO:004001
1//locomotion;G
O:0042127//regu
lation of cell
population
proliferation;GO:
0042471//ear
morphogenesis;G
O:0042474//mid
dle ear
morphogenesis;G
O:0042886//amid
e
transport;GO:004
3009//chordate
embryonic
development;GO
:0043170//macro
molecule
metabolic
process;GO:0043
583//ear
development;GO
:0044237//cellula
r metabolic

process;GO:0044
238//primary
metabolic
process;GO:0044
249//cellular
biosynthetic
process;GO:0044
260//cellular
macromolecule
metabolic
process;GO:0044
271//cellular
nitrogen
compound
biosynthetic
process;GO:0045
165//cell fate
commitment;GO:
0045184//establis
hment of protein
localization;GO:
0045595//regulat
ion of cell
differentiation;G
O:0045596//nega
tive regulation of
cell
differentiation;G
O:0045893//posit
ive regulation of
transcription,
DNA-

templated;GO:0045935//positive regulation of nucleobase-containing compound metabolic process;GO:0045944//positive regulation of transcription by RNA polymerase II;GO:0046483//heterocycle metabolic process;GO:0046907//intracellular transport;GO:0048332//mesoderm morphogenesis;GO:0048333//mesodermal cell differentiation;GO:0048513//animal organ development;GO:0048518//positive regulation of biological process;GO:0048519//negative regulation of

biological
process;GO:0048
522//positive
regulation of
cellular
process;GO:0048
523//negative
regulation of
cellular
process;GO:0048
546//digestive
tract
morphogenesis;G
O:0048557//emb
ryonic digestive
tract
morphogenesis;G
O:0048562//emb
ryonic organ
morphogenesis;G
O:0048565//dige
stive tract
development;GO
:0048566//embry
onic digestive
tract
development;GO
:0048568//embry
onic organ
development;GO
:0048598//embry
onic

morphogenesis;GO:0048646//anatomical structure formation involved in morphogenesis;GO:0048701//embryonic cranial skeleton morphogenesis;GO:0048704//embryonic skeletal system morphogenesis;GO:0048705//skeletal system morphogenesis;GO:0048706//embryonic skeletal system development;GO:0048729//tissue morphogenesis;GO:0048731//system development;GO:0048762//mesenchymal cell differentiation;GO:0048856//anatomical structure development;GO

:0048869//cellula
r developmental
process;GO:0048
870//cell
motility;GO:005
0789//regulation
of biological
process;GO:0050
793//regulation
of developmental
process;GO:0050
794//regulation
of cellular
process;GO:0051
093//negative
regulation of
developmental
process;GO:0051
169//nuclear
transport;GO:005
1170//import into
nucleus;GO:0051
171//regulation
of nitrogen
compound
metabolic
process;GO:0051
173//positive
regulation of
nitrogen
compound
metabolic

process;GO:0051
179//localization;
GO:0051216//car
tilage
development;GO
:0051234//establi
shment of
localization;GO:
0051239//regulat
ion of
multicellular
organismal
process;GO:0051
252//regulation
of RNA
metabolic
process;GO:0051
254//positive
regulation of
RNA metabolic
process;GO:0051
641//cellular
localization;GO:
0051649//establis
hment of
localization in
cell;GO:0051674
//localization of
cell;GO:0055123
//digestive
system
development;GO

:0060231//mesenchymal to epithelial transition;GO:0060255//regulation of macromolecule metabolic process;GO:0060429//epithelium development;GO:0060485//mesenchyme development;GO:0060688//regulation of morphogenesis of a branching structure;GO:0060795//cell fate commitment involved in formation of primary germ layer;GO:0060993//kidney morphogenesis;GO:0061005//cell differentiation involved in kidney development;GO

:0061035//regula
tion of cartilage
development;GO
:0061217//regula
tion of
mesonephros
development;GO
:0061448//conne
ctive tissue
development;GO
:0065007//biolog
ical
regulation;GO:00
70727//cellular
macromolecule
localization;GO:
0071702//organic
substance
transport;GO:007
1704//organic
substance
metabolic
process;GO:0071
705//nitrogen
compound
transport;GO:007
2001//renal
system
development;GO
:0072006//nephr
on
development;GO

:0072009//nephr
on epithelium
development;GO
:0072028//nephr
on
morphogenesis;G
O:0072038//mes
enchymal stem
cell maintenance
involved in
nephron
morphogenesis;G
O:0072073//kidn
ey epithelium
development;GO
:0072074//kidney
mesenchyme
development;GO
:0072077//renal
vesicle
morphogenesis;G
O:0072087//renal
vesicle
development;GO
:0072088//nephr
on epithelium
morphogenesis;G
O:0072089//stem
cell
proliferation;GO:
0072137//conden
sed

mesenchymal
cell
proliferation;GO:
0072161//mesenc
hymal cell
differentiation
involved in
kidney
development;GO
:0072202//cell
differentiation
involved in
metanephros
development;GO
:0072210//metan
ephric nephron
development;GO
:0072273//metan
ephric nephron
morphogenesis;G
O:0072283//meta
nephric renal
vesicle
morphogenesis;G
O:0072594//esta
blishment of
protein
localization to
organelle;GO:00
80090//regulatio
n of primary
metabolic

process;GO:0090
183//regulation
of kidney
development;GO
:0090189//regula
tion of branching
involved in
ureteric bud
morphogenesis;G
O:0090304//nucl
eic acid
metabolic
process;GO:0090
596//sensory
organ
morphogenesis;G
O:0097168//mes
enchymal stem
cell
proliferation;GO:
0097659//nucleic
acid-templated
transcription;GO:
0098727//mainte
nance of cell
number;GO:1901
360//organic
cyclic compound
metabolic
process;GO:1901
362//organic
cyclic compound

biosynthetic
process;GO:1901
576//organic
substance
biosynthetic
process;GO:1902
680//positive
regulation of
RNA
biosynthetic
process;GO:1902
732//positive
regulation of
chondrocyte
proliferation;GO:
1903506//regulat
ion of nucleic
acid-templated
transcription;GO:
1903508//positiv
e regulation of
nucleic acid-
templated
transcription;GO:
1904888//cranial
skeletal system
development;GO
:1905330//regula
tion of
morphogenesis
of an
epithelium;GO:2

														000026//regulation of multicellular organismal development;GO:2000027//regulation of animal organ morphogenesis;GO:2000112//regulation of cellular macromolecule biosynthetic process;GO:2001012//mesenchymal cell differentiation involved in renal system development;GO:2001141//regulation of RNA biosynthetic process	
ncbi_113	Slc35a3	7.74	1.321667	1.554333	1.981667	1.799333	XP_026169135.1 UDP-N-acetylglu	-	-	-	-	GO:0000139//Golgi membrane;GO:0016020//membrane;GO:0016021//integral component of membrane	GO:0015165//pyrimidine nucleotide-sugar transmembrane transporter activity	GO:0090481//pyrimidine nucleotide-sugar transmembrane transport	- -

134113							cosamine transporter-like [Mastacembelus armatus]								
ncbi_113135199	TMPRSS9	52.66367	1.116667	1.586333	4.221	4.871333	XP_026170760.1 transmembrane protease serine 9-like [Mastacembelus armatus]	-	-	-	-	-	GO:0004252//serine-type endopeptidase activity	GO:0006508//proteolysis	- -
n	T	8	3	3	4	4	XP_	-	-	-	-	-	GO:0003677//D	GO:0006355//reg	T

cbi_113135803	EF	.527333	.643667	.719667	.275	.094	026171880.1 thyrotroph embryonic factor-like isoform X1 [Mastacembelus armatus]						NA binding;GO:0003700//DNA-binding transcription factor activity	ulation of transcription, DNA-templated	F_bZIP
ncbi_113131137	SIPA1L1	2.121667	1.118	0.849	1.344	1.396	XP_026174063.1 signal-induced proliferation-	-	-	-	-	GO:0005575//cellular_component;GO:0005622//intracellular anatomical structure;GO:0005737//cytoplasm;GO:0005856//cytoskeleton;GO:0005886//plasma membrane;GO:0	GO:0003674//molecular_function;GO:0003779//actin binding;GO:0005096//GTPase activator activity;GO:0005102//signaling receptor binding;GO:0005	GO:0006996//organelle organization;GO:0007010//cytoskeleton organization;GO:0007154//cell communication;GO:0007165//signal transduction;GO:	--

0 1 2	associated 1-like protein 1 [Mastacembelus armatus]	014069//postsynaptic density;GO:0016020//membrane;GO:0030054//cell junction;GO:0030425//dendrite;GO:0032279//asymmetric synapse;GO:0032991//protein-containing complex;GO:0036477//somatodendritic compartment;GO:0042995//cell projection;GO:0043005//neuron projection;GO:0043025//neuronal cell body;GO:0043197//dendritic spine;GO:0043226//organelle;GO:0043228//non-membrane-bounded organelle;GO:0043229//intracellu	488//binding;GO:0005515//protein binding;GO:0008047//enzyme activator activity;GO:0008092//cytoskeletal protein binding;GO:0019899//enzyme binding;GO:0019900//kinase binding;GO:0019901//protein kinase binding;GO:0030234//enzyme regulator activity;GO:0030695//GTPase regulator activity;GO:0031625//ubiquitin protein ligase binding;GO:0044389//ubiquitin-like protein ligase binding;GO:0044877//protein-containing	0007166//cell surface receptor signaling pathway;GO:0007167//enzyme linked receptor protein signaling pathway;GO:0007169//transmembrane receptor protein tyrosine kinase signaling pathway;GO:0007275//multicellular organism development;GO:0007399//nervous system development;GO:0008150//biological_process;GO:0009966//regulation of signal transduction;GO:0009987//cellular process;GO:0010646//regulation of cell communication;GO:0010769//regulation of cell morphogenesis

lar organelle;GO:0043232//intracellular non-membrane-bounded organelle;GO:0044297//cell body;GO:0044309//neuron spine;GO:0045202//synapse;GO:0045211//postsynaptic membrane;GO:0071944//cell periphery;GO:0097060//synaptic membrane;GO:0097447//dendritic tree;GO:0098590//plasma membrane region;GO:0098794//postsynapse;GO:0098984//neuron to neuron synapse;GO:0099572//postsynaptic specialization;G	complex binding;GO:0046875//ephrin receptor binding;GO:0051015//actin filament binding;GO:0060589//nucleoside-triphosphatase regulator activity;GO:0098772//molecular function regulator	involved in differentiation;GO:0010975//regulation of neuron projection development;GO:0016043//cellular component organization;GO:0022008//neurogenesis;GO:0022603//regulation of anatomical structure morphogenesis;GO:0022604//regulation of cell morphogenesis;GO:0023051//regulation of signaling;GO:0023052//signaling;GO:0030029//actin filament-based process;GO:0030036//actin cytoskeleton organization;GO:0030154//cell differentiation;GO:0031344//regulation of cell

O:0120025//plasma membrane bounded cell projection	projection organization;GO:0031532//actin cytoskeleton reorganization;GO:0032501//multicellular organismal process;GO:0032502//developmental process;GO:0043085//positive regulation of catalytic activity;GO:0043087//regulation of GTPase activity;GO:0043093//FtsZ-dependent cytokinesis;GO:0043547//positive regulation of GTPase activity;GO:0044093//positive regulation of molecular function;GO:0045595//regulation of cell

differentiation;G
O:0045664//regu
lation of neuron
differentiation;G
O:0048013//ephr
in receptor
signaling
pathway;GO:004
8167//regulation
of synaptic
plasticity;GO:00
48583//regulatio
n of response to
stimulus;GO:004
8699//generation
of
neurons;GO:004
8731//system
development;GO
:0048814//regula
tion of dendrite
morphogenesis;G
O:0048856//anat
omical structure
development;GO
:0048869//cellula
r developmental
process;GO:0050
767//regulation
of
neurogenesis;GO
:0050770//regula

tion of
axonogenesis;G
O:0050773//regu
lation of dendrite
development;GO
:0050789//regula
tion of biological
process;GO:0050
790//regulation
of catalytic
activity;GO:0050
793//regulation
of developmental
process;GO:0050
794//regulation
of cellular
process;GO:0050
803//regulation
of synapse
structure or
activity;GO:0050
804//modulation
of chemical
synaptic
transmission;GO:
0050807//regulat
ion of synapse
organization;GO:
0050896//respon
se to
stimulus;GO:005
1056//regulation

of small GTPase mediated signal transduction;GO:0051128//regulation of cellular component organization;GO:0051239//regulation of multicellular organismal process;GO:0051336//regulation of hydrolase activity;GO:0051345//positive regulation of hydrolase activity;GO:0051716//cellular response to stimulus;GO:0051960//regulation of nervous system development;GO:0060284//regulation of cell development;GO:0060998//regulation of dendritic spine

development;GO:0061001//regulation of dendritic spine morphogenesis;GO:0065007//biological regulation;GO:0065008//regulation of biological quality;GO:0065009//regulation of molecular function;GO:0071840//cellular component organization or biogenesis;GO:0090529//cell septum assembly;GO:0090630//activation of GTPase activity;GO:0099175//regulation of postsynapse organization;GO:0099177//regulation of trans-synaptic signaling;GO:0120035//regulatio

														n of plasma membrane bounded cell projection organization;GO:1902531//regulation of intracellular signal transduction;GO:2000026//regulation of multicellular organismal development	
ncbi_113138095	KCNG4	1.380667	0.022667	0.024667	0.113	0	XP_026176027.1 potassium voltage-gated channel subfamily G mem	-	-	-	-	GO:0016020//membrane	GO:0005216//ion channel activity	GO:0006811//ion transport;GO:0051260//protein homooligomerization;GO:0055085//transmembrane transport	- -

							ber 4-like [Mastacembelus armatus]								
ncbi_113138228	CD151	14.36033	5.198667	5.308333	6.918	5.563	XP_026176302.1 CD151 antigen-like [Mastacembelus armatus]	-	-	-	-	GO:0005575//cellular_component;GO:0005576//extracellular region;GO:0005604//basement membrane;GO:0005622//intracellular anatomical structure;GO:0005737//cytoplasm;GO:0005829//cytosol;GO:0005886//plasma membrane;GO:0005887//integral component of plasma membrane;GO:0009326//formate dehydrogenase complex;GO:0009986//cell	GO:0003674//molecular_function;GO:0005102//signaling receptor binding;GO:0005178//integrin binding;GO:0005488//binding;GO:0005515//protein binding;GO:0008863//formate dehydrogenase (NAD+) activity;GO:0009055//electron transfer activity;GO:0044877//protein-containing complex binding;GO:0050839//cell	GO:0001775//cell activation;GO:0002009//morphogenesis of an epithelium;GO:0002011//morphogenesis of an epithelial sheet;GO:0002376//immune system process;GO:0006928//movement of cell or subcellular component;GO:0006950//response to stress;GO:0007044//cell-substrate junction assembly;GO:00	- -

surface;GO:0016020//membrane;GO:0016021//integral component of membrane;GO:0031012//extracellular matrix;GO:0031224//intrinsic component of membrane;GO:0031226//intrinsic component of plasma membrane;GO:0062023//collagen-containing extracellular matrix;GO:0071944//cell periphery	adhesion molecule binding	07154//cell communication;GO:0007155//cell adhesion;GO:0007165//signal transduction;GO:0007166//cell surface receptor signaling pathway;GO:0008150//biological_process;GO:0008283//cell population proliferation;GO:0009061//anaerobic respiration;GO:0009611//response to wounding;GO:0009653//anatomical structure morphogenesis;GO:0009888//tissue development;GO:0009987//cellular process;GO:0016043//cellular

component
organization;GO:
0016477//cell
migration;GO:00
22607//cellular
component
assembly;GO:00
22610//biological
adhesion;GO:002
3052//signaling;
GO:0030100//reg
ulation of
endocytosis;GO:
0030334//regulat
ion of cell
migration;GO:00
30335//positive
regulation of cell
migration;GO:00
31581//hemides
mosome
assembly;GO:00
32502//developm
ental
process;GO:0032
879//regulation
of
localization;GO:
0032943//monon
uclear cell
proliferation;GO:
0034329//cell

junction
assembly;GO:00
34330//cell
junction
organization;GO:
0040011//locomo
tion;GO:0040012
//regulation of
locomotion;GO:0
040017//positive
regulation of
locomotion;GO:0
042060//wound
healing;GO:0042
098//T cell
proliferation;GO:
0042110//T cell
activation;GO:00
44085//cellular
component
biogenesis;GO:0
044319//wound
healing,
spreading of
cells;GO:004532
1//leukocyte
activation;GO:00
45807//positive
regulation of
endocytosis;GO:
0046649//lymph
ocyte

activation;GO:00
46651//lymphocy
te
proliferation;GO:
0048518//positiv
e regulation of
biological
process;GO:0048
522//positive
regulation of
cellular
process;GO:0048
729//tissue
morphogenesis;G
O:0048856//anat
omical structure
development;GO
:0048870//cell
motility;GO:005
0789//regulation
of biological
process;GO:0050
794//regulation
of cellular
process;GO:0050
896//response to
stimulus;GO:005
1049//regulation
of
transport;GO:005
1050//positive
regulation of

transport;GO:005
1128//regulation
of cellular
component
organization;GO:
0051130//positiv
e regulation of
cellular
component
organization;GO:
0051179//localiz
ation;GO:005127
0//regulation of
cellular
component
movement;GO:0
051272//positive
regulation of
cellular
component
movement;GO:0
051674//localizat
ion of
cell;GO:0051716
//cellular
response to
stimulus;GO:006
0429//epithelium
development;GO
:0060627//regula
tion of vesicle-
mediated

														transport;GO:0065007//biological regulation;GO:0070661//leukocyte proliferation;GO:0071840//cellular component organization or biogenesis;GO:0090504//epiboly;GO:0090505//epiboly involved in wound healing;GO:2000145//regulation of cell motility;GO:2000147//positive regulation of cell motility	
ncbi_113131383	Rgs12	2.631	0.728	0.729	0.885	0.838	XP_026176452.1 regulator of G-protein signaling	-	-	-	-	GO:0019031//viral envelope;GO:0030288//outer membrane-bounded periplasmic space	GO:0004252//serine-type endopeptidase activity;GO:0005515//protein binding;GO:0030695//GTPase regulator activity	GO:0006508//proteolysis;GO:0006950//response to stress;GO:0007165//signal transduction	- -

36							12-like isoform X1 [Mastacembelus armatus]								
ncbi_113138903	Nkx6-3	17.65067	0.081	0.591667	0.097667	1.623667	XP_026177532.1 homeobox protein Nkx-6.3 [Mastacembelus armatus]	-	-	-	-	GO:0005575//cellular_component;GO:0005622//intracellular anatomical structure;GO:0005634//nucleus;GO:0043226//organelle;GO:0043227//membrane-bounded organelle;GO:0043229//intracellular organelle;GO:0043231//intracellular membrane-bounded organelle	GO:0000981//DNA-binding transcription factor activity, RNA polymerase II-specific;GO:0001228//DNA-binding transcription activator activity, RNA polymerase II-specific;GO:0003674//molecular_function;GO:0003676//nucleic acid binding;GO:0003677//DNA binding;GO:0003	GO:0001709//cell fate determination;GO:0002065//columnar/cuboidal epithelial cell differentiation;GO:0002067//glandular epithelial cell differentiation;GO:0006139//nucleobase-containing compound metabolic process;GO:0006351//transcription, DNA-templated;GO:0006355//regulatio	Homeobox

700//DNA-binding transcription factor activity;GO:0005488//binding;GO:0043565//sequence-specific DNA binding;GO:0097159//organic cyclic compound binding;GO:0140110//transcription regulator activity;GO:1901363//heterocyclic compound binding	n of transcription, DNA-templated;GO:0006357//regulation of transcription by RNA polymerase II;GO:0006366//transcription by RNA polymerase II;GO:0006725//cellular aromatic compound metabolic process;GO:0006807//nitrogen compound metabolic process;GO:0008150//biological_process;GO:0008152//metabolic process;GO:0009058//biosynthetic process;GO:0009059//macromolecule biosynthetic process;GO:0009888//tissue development;GO:0009889//regula

tion of
biosynthetic
process;GO:0009
891//positive
regulation of
biosynthetic
process;GO:0009
893//positive
regulation of
metabolic
process;GO:0009
987//cellular
process;GO:0010
467//gene
expression;GO:0
010468//regulati
on of gene
expression;GO:0
010556//regulati
on of
macromolecule
biosynthetic
process;GO:0010
557//positive
regulation of
macromolecule
biosynthetic
process;GO:0010
604//positive
regulation of
macromolecule
metabolic

process;GO:0010
628//positive
regulation of
gene
expression;GO:0
016070//RNA
metabolic
process;GO:0018
130//heterocycle
biosynthetic
process;GO:0019
219//regulation
of nucleobase-
containing
compound
metabolic
process;GO:0019
222//regulation
of metabolic
process;GO:0019
438//aromatic
compound
biosynthetic
process;GO:0030
154//cell
differentiation;G
O:0030855//epith
elial cell
differentiation;G
O:0030856//regu
lation of
epithelial cell

differentiation;GO:0030857//negative regulation of epithelial cell differentiation;GO:0031323//regulation of cellular metabolic process;GO:0031325//positive regulation of cellular metabolic process;GO:0031326//regulation of cellular biosynthetic process;GO:0031328//positive regulation of cellular biosynthetic process;GO:0032502//developmental process;GO:0032774//RNA biosynthetic process;GO:0034641//cellular nitrogen compound

metabolic
process;GO:0034
645//cellular
macromolecule
biosynthetic
process;GO:0034
654//nucleobase-
containing
compound
biosynthetic
process;GO:0043
170//macromolec
ule metabolic
process;GO:0044
237//cellular
metabolic
process;GO:0044
238//primary
metabolic
process;GO:0044
249//cellular
biosynthetic
process;GO:0044
260//cellular
macromolecule
metabolic
process;GO:0044
271//cellular
nitrogen
compound
biosynthetic
process;GO:0045

165//cell fate commitment;GO:0045595//regulation of cell differentiation;GO:0045596//negative regulation of cell differentiation;GO:0045893//positive regulation of transcription, DNA-templated;GO:0045935//positive regulation of nucleobase-containing compound metabolic process;GO:0045944//positive regulation of transcription by RNA polymerase II;GO:0046483//heterocycle metabolic process;GO:0048518//positive regulation of biological

process;GO:0048519//negative regulation of biological process;GO:0048522//positive regulation of cellular process;GO:0048523//negative regulation of cellular process;GO:0048856//anatomical structure development;GO:0048869//cellular developmental process;GO:0050789//regulation of biological process;GO:0050793//regulation of developmental process;GO:0050794//regulation of cellular process;GO:0051093//negative regulation of developmental process;GO:0051

171//regulation
of nitrogen
compound
metabolic
process;GO:0051
173//positive
regulation of
nitrogen
compound
metabolic
process;GO:0051
239//regulation
of multicellular
organismal
process;GO:0051
252//regulation
of RNA
metabolic
process;GO:0051
254//positive
regulation of
RNA metabolic
process;GO:0060
255//regulation
of
macromolecule
metabolic
process;GO:0060
429//epithelium
development;GO
:0065007//biolog
ical

regulation;GO:00
71704//organic
substance
metabolic
process;GO:0080
090//regulation
of primary
metabolic
process;GO:0090
304//nucleic acid
metabolic
process;GO:0097
659//nucleic
acid-templated
transcription;GO:
1901360//organic
cyclic compound
metabolic
process;GO:1901
362//organic
cyclic compound
biosynthetic
process;GO:1901
576//organic
substance
biosynthetic
process;GO:1902
680//positive
regulation of
RNA
biosynthetic
process;GO:1903

														506//regulation of nucleic acid-templated transcription;GO:1903508//positive regulation of nucleic acid-templated transcription;GO:2000026//regulation of multicellular organismal development;GO:2000112//regulation of cellular macromolecule biosynthetic process;GO:2001141//regulation of RNA biosynthetic process	
ncbi_11131	ADGRD2	1.004	0.209667	0.181	0.173	0.249667	XP_026179293.1 adhesion G-protein	-	-	-	-	GO:0016021//integral component of membrane	GO:0004930//G protein-coupled receptor activity	GO:0007186//G protein-coupled receptor signaling pathway	- -

39889							coupled receptor D2-like isoform X1 [Mastacembelus armatus]								
ncbi_113141144	emp2	7.998333	3.717667	3.524	4.359667	3.303333	XP_026181165.1 epithelial membrane protein 2 [Mastacembelus armatus]	-	-	-	-	GO:0000139//Golgi membrane;GO:0005575//cellular_component;GO:0005622//intracellular anatomical structure;GO:0005634//nucleus;GO:0005737//cytoplasm;GO:0005794//Golgi apparatus;GO:0005829//cytosol;GO:0005886//plasma membrane;GO:0	GO:0003674//molecular_function;GO:0005102//signaling receptor binding;GO:0005178//integrin binding;GO:0005488//binding;GO:0005515//protein binding;GO:0019899//enzyme binding;GO:0019900//kinase binding;GO:0019901//protein kinase	GO:0000003//reproduction;GO:0001667//ameboidal-type cell migration;GO:0001765//membrane raft assembly;GO:0001906//cell killing;GO:0001909//leukocyte mediated cytotoxicity;GO:0001913//T cell mediated cytotoxicity;GO:0001932//regulat	-<br>-

009986//cell surface;GO:0012505//endomembrane system;GO:0016020//membrane;GO:0016021//integral component of membrane;GO:0016324//apical plasma membrane;GO:0031090//organelle membrane;GO:0031224//intrinsic component of membrane;GO:0031410//cytoplasmic vesicle;GO:0031982//vesicle;GO:0031984//organelle subcompartment;GO:0043226//organelle;GO:0043227//membrane-bounded organelle;GO:0043229//intracellu	binding;GO:0044877//protein-containing complex binding;GO:0050839//cell adhesion molecule binding	ion of protein phosphorylation;GO:0001934//positive regulation of protein phosphorylation;GO:0001952//regulation of cell-matrix adhesion;GO:0001954//positive regulation of cell-matrix adhesion;GO:0001977//renal system process involved in regulation of blood volume;GO:0002250//adaptive immune response;GO:0002252//immune effector process;GO:0002376//immune system process;GO:0002443//leukocyte mediated immunity;GO:00

lar organelle;GO:0043231//intracellular membrane-bounded organelle;GO:0045121//membrane raft;GO:0045177//apical part of cell;GO:0071944//cell periphery;GO:0097708//intracellular vesicle;GO:0098588//bounding membrane of organelle;GO:0098590//plasma membrane region;GO:0098791//Golgi apparatus subcompartment;GO:0098857//membrane microdomain	02449//lymphocyte mediated immunity;GO:0002456//T cell mediated immunity;GO:0002460//adaptive immune response based on somatic recombination of immune receptors built from immunoglobulin superfamily domains;GO:0003008//system process;GO:0003013//circulatory system process;GO:0003014//renal system process;GO:0003071//renal system process involved in regulation of systemic arterial blood pressure;GO:0003073//regulation

of systemic
arterial blood
pressure;GO:000
3093//regulation
of glomerular
filtration;GO:000
6810//transport;
GO:0006928//mo
vement of cell or
subcellular
component;GO:0
006955//immune
response;GO:000
6996//organelle
organization;GO:
0007010//cytosk
eleton
organization;GO:
0007015//actin
filament
organization;GO:
0007155//cell
adhesion;GO:000
7160//cell-matrix
adhesion;GO:000
7275//multicellul
ar organism
development;GO
:0007565//female
pregnancy;GO:0
007566//embryo
implantation;GO:

0008015//blood circulation;GO:0008104//protein localization;GO:0008150//biological_process;GO:0008217//regulation of blood pressure;GO:0008219//cell death;GO:0008283//cell population proliferation;GO:0008284//positive regulation of cell population proliferation;GO:0009893//positive regulation of metabolic process;GO:0009966//regulation of signal transduction;GO:0009967//positive regulation of signal transduction;GO:0009987//cellular process;GO:0010562//positive

regulation of
phosphorus
metabolic
process;GO:0010
594//regulation
of endothelial
cell
migration;GO:00
10604//positive
regulation of
macromolecule
metabolic
process;GO:0010
631//epithelial
cell
migration;GO:00
10632//regulatio
n of epithelial
cell
migration;GO:00
10646//regulatio
n of cell
communication;
GO:0010647//po
sitive regulation
of cell
communication;
GO:0010810//reg
ulation of cell-
substrate
adhesion;GO:001
0811//positive

regulation of
cell-substrate
adhesion;GO:001
6043//cellular
component
organization;GO:
0016192//vesicle
-mediated
transport;GO:001
6477//cell
migration;GO:00
16482//cytosolic
transport;GO:001
9220//regulation
of phosphate
metabolic
process;GO:0019
222//regulation
of metabolic
process;GO:0022
414//reproductiv
e
process;GO:0022
603//regulation
of anatomical
structure
morphogenesis;G
O:0022607//cellu
lar component
assembly;GO:00
22610//biological
adhesion;GO:002

3051//regulation
of
signaling;GO:00
23056//positive
regulation of
signaling;GO:00
30029//actin
filament-based
process;GO:0030
030//cell
projection
organization;GO:
0030031//cell
projection
assembly;GO:00
30036//actin
cytoskeleton
organization;GO:
0030048//actin
filament-based
movement;GO:0
030155//regulati
on of cell
adhesion;GO:003
0334//regulation
of cell
migration;GO:00
31323//regulatio
n of cellular
metabolic
process;GO:0031
325//positive

regulation of
cellular
metabolic
process;GO:0031
399//regulation
of protein
modification
process;GO:0031
401//positive
regulation of
protein
modification
process;GO:0031
579//membrane
raft
organization;GO:
0031589//cell-
substrate
adhesion;GO:003
2060//bleb
assembly;GO:00
32147//activation
of protein kinase
activity;GO:0032
268//regulation
of cellular
protein metabolic
process;GO:0032
270//positive
regulation of
cellular protein
metabolic

process;GO:0032501//multicellular organismal process;GO:0032502//developmental process;GO:0032879//regulation of localization;GO:0033036//macromolecule localization;GO:0033674//positive regulation of kinase activity;GO:0034394//protein localization to cell surface;GO:0034613//cellular protein localization;GO:0040011//locomotion;GO:0040012//regulation of locomotion;GO:0042127//regulation of cell population proliferation;GO:

0042325//regulat
ion of
phosphorylation;
GO:0042327//po
sitive regulation
of
phosphorylation;
GO:0043085//po
sitive regulation
of catalytic
activity;GO:0043
534//blood vessel
endothelial cell
migration;GO:00
43542//endotheli
al cell
migration;GO:00
43549//regulatio
n of kinase
activity;GO:0044
057//regulation
of system
process;GO:0044
085//cellular
component
biogenesis;GO:0
044091//membra
ne
biogenesis;GO:0
044093//positive
regulation of
molecular

function;GO:004
4703//multi-
organism
reproductive
process;GO:0044
706//multi-
multicellular
organism
process;GO:0045
022//early
endosome to late
endosome
transport;GO:004
5595//regulation
of cell
differentiation;G
O:0045765//regu
lation of
angiogenesis;GO
:0045785//positiv
e regulation of
cell
adhesion;GO:004
5859//regulation
of protein kinase
activity;GO:0045
860//positive
regulation of
protein kinase
activity;GO:0045
937//positive
regulation of

phosphate
metabolic
process;GO:0046
907//intracellular
transport;GO:004
8518//positive
regulation of
biological
process;GO:0048
522//positive
regulation of
cellular
process;GO:0048
583//regulation
of response to
stimulus;GO:004
8584//positive
regulation of
response to
stimulus;GO:004
8856//anatomical
structure
development;GO
:0048870//cell
motility;GO:005
0789//regulation
of biological
process;GO:0050
790//regulation
of catalytic
activity;GO:0050
793//regulation

of developmental process;GO:0050794//regulation of cellular process;GO:0050878//regulation of body fluid levels;GO:0050896//response to stimulus;GO:0051171//regulation of nitrogen compound metabolic process;GO:0051173//positive regulation of nitrogen compound metabolic process;GO:0051174//regulation of phosphorus metabolic process;GO:0051179//localization;GO:0051234//establishment of localization;GO:0051239//regulation of multicellular

organismal
process;GO:0051
246//regulation
of protein
metabolic
process;GO:0051
247//positive
regulation of
protein metabolic
process;GO:0051
270//regulation
of cellular
component
movement;GO:0
051338//regulati
on of transferase
activity;GO:0051
347//positive
regulation of
transferase
activity;GO:0051
641//cellular
localization;GO:
0051649//establis
hment of
localization in
cell;GO:0051674
//localization of
cell;GO:0051704
//multi-organism
process;GO:0060
255//regulation

of
macromolecule
metabolic
process;GO:0061
024//membrane
organization;GO:
0065007//biologi
cal
regulation;GO:00
65008//regulatio
n of biological
quality;GO:0065
009//regulation
of molecular
function;GO:007
0252//actin-
mediated cell
contraction;GO:0
070727//cellular
macromolecule
localization;GO:
0071709//membr
ane
assembly;GO:00
71840//cellular
component
organization or
biogenesis;GO:0
072657//protein
localization to
membrane;GO:0
072659//protein

localization to plasma membrane;GO:0080090//regulation of primary metabolic process;GO:0090130//tissue migration;GO:0090132//epithelium migration;GO:0097435//supramolecular fiber organization;GO:0098801//regulation of renal system process;GO:0098927//vesicle-mediated transport between endosomal compartments;GO:0120031//plasma membrane bounded cell projection assembly;GO:0120036//plasma membrane

bounded cell projection organization;GO:1901342//regulation of vasculature development;GO:1990778//protein localization to cell periphery;GO:2000026//regulation of multicellular organismal development;GO:2000145//regulation of cell motility;GO:2001044//regulation of integrin-mediated signaling pathway;GO:2001046//positive regulation of integrin-mediated signaling pathway;GO:2001212//regulation of vasculogenesis

ncbi_113141246	gsg1l	0.940667	0	0.209333	0.409	0.123667	XP_026181317.1 germ cell-specific gene 1-like protein [Mastacembelus armatus]	-	-	-	-	GO:0005575//cellular_component;GO:0005886//plasma membrane;GO:0016020//membrane;GO:0016021//integral component of membrane;GO:0030054//cell junction;GO:0031224//intrinsic component of membrane;GO:0045202//synapse;GO:0071944//cell periphery	-	-	- -
ncbi_113141177	Cpm	1.91	0.641333	0.646667	0.845667	0.712	XP_026182099.1 carboxypeptidase M [Mastacembelus	-	-	-	-	GO:0005575//cellular_component;GO:0005576//extracellular region;GO:0005615//extracellular space;GO:0005886//plasma membrane;GO:0009986//cell surface;GO:0016020//membrane;GO:0031224//int	GO:0003674//molecular_function;GO:0003824//catalytic activity;GO:0004180//carboxypeptidase activity;GO:0004181//metallocarboxypeptidase activity;GO:0004185//serine-type carboxypeptidase	GO:0006508//proteolysis;GO:0006518//peptide metabolic process;GO:0006807//nitrogen compound metabolic process;GO:0008150//biological_process;GO:0008152//metabolic process;GO:0009	- -

8	arma tus]		rinsic component of membrane;GO:0031225//anchored component of membrane;GO:0071944//cell periphery	activity;GO:0005488//binding;GO:0008233//peptidase activity;GO:0008235//metalloexopeptidase activity;GO:0008236//serine-type peptidase activity;GO:0008237//metallopeptidase activity;GO:0008238//exopeptidase activity;GO:0008270//zinc ion binding;GO:0016787//hydrolase activity;GO:0016788//hydrolase activity, acting on ester bonds;GO:0017171//serine hydrolase activity;GO:0018578//protocatechuate 3,4-dioxygenase activity;GO:0043	653//anatomical structure morphogenesis;GO:0009987//cellular process;GO:0010467//gene expression;GO:0016485//protein processing;GO:0019538//protein metabolic process;GO:0032502//developmental process;GO:0034641//cellular nitrogen compound metabolic process;GO:0043170//macromolecule metabolic process;GO:0043603//cellular amide metabolic process;GO:0044237//cellular metabolic process;GO:0044238//primary metabolic

													167//ion binding;GO:0043169//cation binding;GO:0046872//metal ion binding;GO:0046914//transition metal ion binding;GO:0070008//serine-type exopeptidase activity;GO:0140096//catalytic activity, acting on a protein	process;GO:0046278//3,4-dihydroxybenzoate metabolic process;GO:0048856//anatomical structure development;GO:0051604//protein maturation;GO:0071704//organic substance metabolic process;GO:1901564//organonitrogen compound metabolic process	
ncbi_11314225	PRMT8	2.007333	0.321333	0.095333	0.657667	0.346333	XP_0261829 70.1 protein arginine N-methyltransferase	-	-	-	-	GO:0005575//cellular_component;GO:0005622//intracellular anatomical structure;GO:0005737//cytoplasm;GO:0005829//cytosol;GO:0005886//plasma membrane;GO:0009898//cytoplasmic side of	GO:0003674//molecular_function;GO:0003824//catalytic activity;GO:0004719//protein-L-isoaspartate (D-aspartate) O-methyltransferase activity;GO:0005488//binding;GO:0005515//protei	GO:0000902//cell morphogenesis;GO:0000904//cell morphogenesis involved in differentiation;GO:0003002//regionalization;GO:0006325//chromatin organization;GO:0006355//regulat	- -

7	8-B-like isoform X1 [Mastacembelus armatus]		plasma membrane;GO:0016020//membrane;GO:0031224//intrinsic component of membrane;GO:0031225//anchored component of membrane;GO:0031226//intrinsic component of plasma membrane;GO:0031235//intrinsic component of the cytoplasmic side of the plasma membrane;GO:0046658//anchored component of plasma membrane;GO:0071944//cell periphery;GO:0098552//side of membrane;GO:0098562//cytoplasmic side of membrane;GO:0098753//anchored component of	n binding;GO:0008168//methyltransferase activity;GO:0008170//N-methyltransferase activity;GO:0008171//O-methyltransferase activity;GO:0008276//protein methyltransferase activity;GO:0008469//histone-arginine N-methyltransferase activity;GO:0008689//3-demethylubiquinone-9 3-O-methyltransferase activity;GO:0008757//S-adenosylmethionine-dependent methyltransferas	ion of transcription, DNA-templated;GO:0006400//tRNA modification;GO:0006412//translation;GO:0006464//cellular protein modification process;GO:0006479//protein methylation;GO:0006744//ubiquinone biosynthetic process;GO:0006807//nitrogen compound metabolic process;GO:0006996//organelle organization;GO:0007275//multicellular organism development;GO:0007389//pattern specification process;GO:0007399//nervous system development;GO

the cytoplasmic side of the plasma membrane	e activity;GO:0016273//arginine N-methyltransferase activity;GO:0016274//protein-arginine N-methyltransferase activity;GO:0016740//transferase activity;GO:0016741//transferase activity, transferring one-carbon groups;GO:0018024//histone-lysine N-methyltransferase activity;GO:0035241//protein-arginine omega-N monomethyltransferase activity;GO:0035242//protein-arginine omega-N asymmetric	:0007417//central nervous system development;GO:0007420//brain development;GO:0008150//biological_process;GO:0008152//metabolic process;GO:0008213//protein alkylation;GO:0008299//isoprenoid biosynthetic process;GO:0009102//biotin biosynthetic process;GO:0009653//anatomical structure morphogenesis;GO:0009790//embryo development;GO:0009792//embryo development ending in birth or egg hatching;GO:0009888//tissue development;GO:0009889//regula

methyltransferase activity;GO:0042054//histone methyltransferase activity;GO:0042802//identical protein binding;GO:0046025//precorrin-6Y C5,15-methyltransferase (decarboxylating) activity;GO:0046406//magnesium protoporphyrin IX methyltransferase activity;GO:0140096//catalytic activity, acting on a protein;GO:1901681//sulfur compound binding;GO:1904047//S-adenosyl-L-methionine	tion of biosynthetic process;GO:0009952//anterior/posterior pattern specification;GO:0009987//cellular process;GO:0010468//regulation of gene expression;GO:0010556//regulation of macromolecule biosynthetic process;GO:0015995//chlorophyll biosynthetic process;GO:0016043//cellular component organization;GO:0016358//dendrite development;GO:0016570//histone modification;GO:0016571//histone methylation;GO:

					binding	0018193//peptidyl-amino acid modification;GO:0018195//peptidyl-arginine modification;GO:0018216//peptidyl-arginine methylation;GO:0018364//peptidyl-glutamine methylation;GO:0019219//regulation of nucleobase-containing compound metabolic process;GO:0019222//regulation of metabolic process;GO:0019538//protein metabolic process;GO:0019919//peptidyl-arginine methylation, to asymmetrical-dimethyl arginine;GO:0021532//neural tube

patterning;GO:00
21903//rostrocau
dal neural tube
patterning;GO:00
21915//neural
tube
development;GO
:0022008//neuro
genesis;GO:0022
607//cellular
component
assembly;GO:00
30030//cell
projection
organization;GO:
0030091//protein
repair;GO:00301
54//cell
differentiation;G
O:0030182//neur
on
differentiation;G
O:0030917//mid
brain-hindbrain
boundary
development;GO
:0031175//neuro
n projection
development;GO
:0031323//regula
tion of cellular
metabolic

process;GO:0031
326//regulation
of cellular
biosynthetic
process;GO:0032
259//methylation
;GO:0032501//m
ulticellular
organismal
process;GO:0032
502//developmen
tal
process;GO:0032
989//cellular
component
morphogenesis;G
O:0032990//cell
part
morphogenesis;G
O:0034969//histo
ne arginine
methylation;GO:
0035246//peptidy
l-arginine N-
methylation;GO:
0035247//peptidy
l-arginine
omega-N-
methylation;GO:
0035295//tube
development;GO
:0036211//protei

n modification process;GO:0043009//chordate embryonic development;GO:0043170//macromolecule metabolic process;GO:0043412//macromolecule modification;GO:0043414//macromolecule methylation;GO:0043933//protein-containing complex subunit organization;GO:0044085//cellular component biogenesis;GO:0044237//cellular metabolic process;GO:0044238//primary metabolic process;GO:0044260//cellular macromolecule metabolic process;GO:0044

267//cellular protein metabolic process;GO:0046140//corrin biosynthetic process;GO:0048468//cell development;GO:0048513//animal organ development;GO:0048666//neuron development;GO:0048667//cell morphogenesis involved in neuron differentiation;GO:0048699//generation of neurons;GO:0048731//system development;GO:0048812//neuron projection morphogenesis;GO:0048813//dendrite morphogenesis;GO:0048856//anatomical structure

development;GO:0048858//cell projection morphogenesis;GO:0048869//cellular developmental process;GO:0050789//regulation of biological process;GO:0050794//regulation of cellular process;GO:0051171//regulation of nitrogen compound metabolic process;GO:0051252//regulation of RNA metabolic process;GO:0051259//protein complex oligomerization;GO:0051260//protein homooligomerization;GO:0051276//chromosome organization;GO:

0060255//regulation of macromolecule metabolic process;GO:0060322//head development;GO:0060429//epithelium development;GO:0065003//protein-containing complex assembly;GO:0065007//biological regulation;GO:0071704//organic substance metabolic process;GO:0071840//cellular component organization or biogenesis;GO:0080090//regulation of primary metabolic process;GO:0120036//plasma membrane bounded cell projection

														organization;GO:0120039//plasma membrane bounded cell projection morphogenesis;GO:1901564//organonitrogen compound metabolic process;GO:1903506//regulation of nucleic acid-templated transcription;GO:2000112//regulation of cellular macromolecule biosynthetic process;GO:2001141//regulation of RNA biosynthetic process	
ncbi_113	dact2	7.839333	1.950333	2.161667	4.579333	2.531333	XP_0261 85967.1 mucin-5AC-like	-	-	-	-	-	-	GO:0009292//genetic transfer	-<br>-

144275							isoform X1 [Mastacembelus armatus]								
ncbi_113144518	nkx3-2	3.672	0.618333	0.248667	0.995333	1.853667	XP_026186414.1 homeobox protein Nkx-3.2 [Mastacembelus armatus]	-	-	-	-	GO:0005575//cellular_component;GO:0005622//intracellular anatomical structure;GO:0005634//nucleus;GO:0005654//nucleoplasm;GO:0031974//membrane-enclosed lumen;GO:0031981//nuclear lumen;GO:0043226//organelle;GO:0043227//membrane-bounded organelle;GO:0043229//intracellular organelle;GO:0043231//intracellular membrane-	GO:0000976//transcription cis-regulatory region binding;GO:0000977//RNA polymerase II transcription regulatory region sequence-specific DNA binding;GO:0000978//RNA polymerase II cis-regulatory region sequence-specific DNA binding;GO:0000981//DNA-binding transcription factor activity, RNA polymerase II-	GO:0000122//negative regulation of transcription by RNA polymerase II;GO:0001501//skeletal system development;GO:0001704//formation of primary germ layer;GO:0001707//mesoderm formation;GO:0001708//cell fate specification;GO:0001710//mesodermal cell fate commitment;GO:0002064//epithelial cell development;GO:0002065//colum	Homeobox

bounded organelle;GO:0043233//organelle lumen;GO:0070013//intracellular organelle lumen	specific;GO:0000987//cis-regulatory region sequence-specific DNA binding;GO:0001067//transcription regulatory region nucleic acid binding;GO:0001227//DNA-binding transcription repressor activity, RNA polymerase II-specific;GO:0003674//molecular_function;GO:0003676//nucleic acid binding;GO:0003677//DNA binding;GO:0003690//double-stranded DNA binding;GO:0003700//DNA-binding transcription factor	nar/cuboidal epithelial cell differentiation;GO:0002066//columnar/cuboidal epithelial cell development;GO:0002376//immune system process;GO:0002520//immune system development;GO:0006139//nucleobase-containing compound metabolic process;GO:0006351//transcription, DNA-templated;GO:0006355//regulation of transcription, DNA-templated;GO:0006357//regulation of transcription by RNA polymerase II;GO:0006366//transcription by

activity;GO:0005488//binding;GO:0043565//sequence-specific DNA binding;GO:0097159//organic cyclic compound binding;GO:0140110//transcription regulator activity;GO:1901363//heterocyclic compound binding;GO:1990837//sequence-specific double-stranded DNA binding	RNA polymerase II;GO:0006725//cellular aromatic compound metabolic process;GO:0006807//nitrogen compound metabolic process;GO:0007275//multicellular organism development;GO:0007368//determination of left/right symmetry;GO:0007369//gastrulation;GO:0007389//pattern specification process;GO:0007423//sensory organ development;GO:0007494//midgut development;GO:0007498//mesoderm development;GO:0007501//mesod

ermal cell fate
specification;GO
:0007507//heart
development;GO
:0007517//muscl
e organ
development;GO
:0007522//viscer
al muscle
development;GO
:0008150//biolog
ical_process;GO:
0008152//metabo
lic
process;GO:0009
058//biosynthetic
process;GO:0009
059//macromolec
ule biosynthetic
process;GO:0009
653//anatomical
structure
morphogenesis;G
O:0009790//emb
ryo
development;GO
:0009792//embry
o development
ending in birth or
egg
hatching;GO:000
9799//specificati

on of
symmetry;GO:00
09855//determina
tion of bilateral
symmetry;GO:00
09887//animal
organ
morphogenesis;G
O:0009888//tissu
e
development;GO
:0009889//regula
tion of
biosynthetic
process;GO:0009
890//negative
regulation of
biosynthetic
process;GO:0009
891//positive
regulation of
biosynthetic
process;GO:0009
892//negative
regulation of
metabolic
process;GO:0009
893//positive
regulation of
metabolic
process;GO:0009
987//cellular

process;GO:0010
467//gene
expression;GO:0
010468//regulati
on of gene
expression;GO:0
010556//regulati
on of
macromolecule
biosynthetic
process;GO:0010
557//positive
regulation of
macromolecule
biosynthetic
process;GO:0010
558//negative
regulation of
macromolecule
biosynthetic
process;GO:0010
604//positive
regulation of
macromolecule
metabolic
process;GO:0010
605//negative
regulation of
macromolecule
metabolic
process;GO:0010
628//positive

regulation of
gene
expression;GO:0
010629//negative
regulation of
gene
expression;GO:0
010941//regulati
on of cell
death;GO:00160
70//RNA
metabolic
process;GO:0018
130//heterocycle
biosynthetic
process;GO:0019
219//regulation
of nucleobase-
containing
compound
metabolic
process;GO:0019
222//regulation
of metabolic
process;GO:0019
438//aromatic
compound
biosynthetic
process;GO:0030
154//cell
differentiation;G
O:0030855//epith

elial cell
differentiation;GO:0031016//pancreas
development;GO:0031323//regulation of cellular
metabolic
process;GO:0031324//negative
regulation of
cellular
metabolic
process;GO:0031325//positive
regulation of
cellular
metabolic
process;GO:0031326//regulation
of cellular
biosynthetic
process;GO:0031327//negative
regulation of
cellular
biosynthetic
process;GO:0031328//positive
regulation of
cellular
biosynthetic

process;GO:0032
330//regulation
of chondrocyte
differentiation;G
O:0032331//nega
tive regulation of
chondrocyte
differentiation;G
O:0032501//mult
icellular
organismal
process;GO:0032
502//developmen
tal
process;GO:0032
774//RNA
biosynthetic
process;GO:0034
641//cellular
nitrogen
compound
metabolic
process;GO:0034
645//cellular
macromolecule
biosynthetic
process;GO:0034
654//nucleobase-
containing
compound
biosynthetic
process;GO:0035

295//tube development;GO:0042471//ear morphogenesis;GO:0042474//middle ear morphogenesis;GO:0042981//regulation of apoptotic process;GO:0043009//chordate embryonic development;GO:0043066//negative regulation of apoptotic process;GO:0043067//regulation of programmed cell death;GO:0043069//negative regulation of programmed cell death;GO:0043170//macromolecule metabolic process;GO:0043583//ear development;GO:0044237//cellula

r metabolic process;GO:0044238//primary metabolic process;GO:0044249//cellular biosynthetic process;GO:0044260//cellular macromolecule metabolic process;GO:0044271//cellular nitrogen compound biosynthetic process;GO:0045165//cell fate commitment;GO:0045595//regulation of cell differentiation;GO:0045596//negative regulation of cell differentiation;GO:0045892//negative regulation of transcription, DNA-templated;GO:0045893//positive

regulation of
transcription,
DNA-
templated;GO:00
45934//negative
regulation of
nucleobase-
containing
compound
metabolic
process;GO:0045
935//positive
regulation of
nucleobase-
containing
compound
metabolic
process;GO:0045
944//positive
regulation of
transcription by
RNA polymerase
II;GO:0046483//
heterocycle
metabolic
process;GO:0048
332//mesoderm
morphogenesis;G
O:0048333//mes
odermal cell
differentiation;G
O:0048468//cell

development;GO
:0048513//animal
organ
development;GO
:0048518//positiv
e regulation of
biological
process;GO:0048
519//negative
regulation of
biological
process;GO:0048
522//positive
regulation of
cellular
process;GO:0048
523//negative
regulation of
cellular
process;GO:0048
534//hematopoiet
ic or lymphoid
organ
development;GO
:0048536//spleen
development;GO
:0048562//embry
onic organ
morphogenesis;G
O:0048565//dige
stive tract
development;GO

:0048568//embryonic organ development;GO:0048598//embryonic morphogenesis;GO:0048645//animal organ formation;GO:0048646//anatomical structure formation involved in morphogenesis;GO:0048705//skeletal system morphogenesis;GO:0048706//embryonic skeletal system development;GO:0048729//tissue morphogenesis;GO:0048731//system development;GO:0048856//anatomical structure development;GO:0048869//cellular developmental process;GO:0050

789//regulation
of biological
process;GO:0050
793//regulation
of developmental
process;GO:0050
794//regulation
of cellular
process;GO:0051
093//negative
regulation of
developmental
process;GO:0051
171//regulation
of nitrogen
compound
metabolic
process;GO:0051
172//negative
regulation of
nitrogen
compound
metabolic
process;GO:0051
173//positive
regulation of
nitrogen
compound
metabolic
process;GO:0051
239//regulation
of multicellular

organismal
process;GO:0051
241//negative
regulation of
multicellular
organismal
process;GO:0051
252//regulation
of RNA
metabolic
process;GO:0051
253//negative
regulation of
RNA metabolic
process;GO:0051
254//positive
regulation of
RNA metabolic
process;GO:0055
123//digestive
system
development;GO
:0060255//regula
tion of
macromolecule
metabolic
process;GO:0060
429//epithelium
development;GO
:0060548//negati
ve regulation of
cell

death;GO:00605
75//intestinal
epithelial cell
differentiation;G
O:0060576//intes
tinal epithelial
cell
development;GO
:0060795//cell
fate commitment
involved in
formation of
primary germ
layer;GO:006103
5//regulation of
cartilage
development;GO
:0061037//negati
ve regulation of
cartilage
development;GO
:0061061//muscl
e structure
development;GO
:0065007//biolog
ical
regulation;GO:00
71704//organic
substance
metabolic
process;GO:0072
359//circulatory

system development;GO:0080090//regulation of primary metabolic process;GO:0090304//nucleic acid metabolic process;GO:0090596//sensory organ morphogenesis;GO:0097659//nucleic acid-templated transcription;GO:1901360//organic cyclic compound metabolic process;GO:1901362//organic cyclic compound biosynthetic process;GO:1901576//organic substance biosynthetic process;GO:1902679//negative regulation of RNA biosynthetic

process;GO:1902680//positive regulation of RNA biosynthetic process;GO:1903506//regulation of nucleic acid-templated transcription;GO:1903507//negative regulation of nucleic acid-templated transcription;GO:1903508//positive regulation of nucleic acid-templated transcription;GO:2000026//regulation of multicellular organismal development;GO:2000112//regulation of cellular macromolecule biosynthetic process;GO:2000113//negative regulation of

														cellular macromolecule biosynthetic process;GO:2001141//regulation of RNA biosynthetic process	
ncbi_113131445229	ATOH1	2.564	6.8973333	8.517	5.6133333	7.9493333	XP_033181308.1 protein atonal homolog 1-like [Mastacembelus armatus]	-	-	-	-	-	GO:0046983//protein dimerization activity	-	bHLH
ncbi_1	MCF2	1.92336	0.57996	0.584	0.81833	0.60933	XP_026186636.1 proto-	-	-	-	-	GO:0005575//cellular_component;GO:0005622//intracellular anatomical structure;GO:000	GO:0003674//molecular_function;GO:0005085//guanyl-nucleotide exchange factor activity;GO:0005	GO:0007059//chromosome segregation;GO:0007154//cell communication;GO:0007165//sig	--

13144646		67	67		33	33	oncogene DBL [Mastacembelus armatus]	5737//cytoplasm;GO:0005829//cytosol;GO:0005856//cytoskeleton;GO:0016020//membrane;GO:0043226//organelle;GO:0043228//non-membrane-bounded organelle;GO:0043229//intracellular organelle;GO:0043232//intracellular non-membrane-bounded organelle	488//binding;GO:0005515//protein binding;GO:0005524//ATP binding;GO:0019899//enzyme binding;GO:0031267//small GTPase binding;GO:0051020//GTPase binding;GO:0098772//molecular function regulator	nal transduction;GO:0007186//G protein-coupled receptor signaling pathway;GO:0007275//multicellular organism development;GO:0007399//nervous system development;GO:0008150//biological_process;GO:0009966//regulation of signal transduction;GO:0009987//cellular process;GO:0010646//regulation of cell communication;GO:0010721//negative regulation of cell development;GO:0010769//regulation of cell morphogenesis involved in differentiation;G

O:0010771//negative regulation of cell morphogenesis involved in differentiation;GO:0010941//regulation of cell death;GO:0010942//positive regulation of cell death;GO:0010975//regulation of neuron projection development;GO:0010977//negative regulation of neuron projection development;GO:0022008//neurogenesis;GO:0022603//regulation of anatomical structure morphogenesis;GO:0022604//regulation of cell morphogenesis;GO:0023051//regulation of

signaling;GO:0023052//signaling;GO:0030154//cell differentiation;GO:0030261//chromosome condensation;GO:0031344//regulation of cell projection organization;GO:0031345//negative regulation of cell projection organization;GO:0032501//multicellular organismal process;GO:0032502//developmental process;GO:0035023//regulation of Rho protein signal transduction;GO:0035556//intracellular signal transduction;GO:0042981//regulation of apoptotic process;GO:0043

065//positive
regulation of
apoptotic
process;GO:0043
067//regulation
of programmed
cell
death;GO:00430
68//positive
regulation of
programmed cell
death;GO:00455
95//regulation of
cell
differentiation;G
O:0045596//nega
tive regulation of
cell
differentiation;G
O:0045664//regu
lation of neuron
differentiation;G
O:0045665//nega
tive regulation of
neuron
differentiation;G
O:0046578//regu
lation of Ras
protein signal
transduction;GO:
0048518//positiv
e regulation of

biological
process;GO:0048
519//negative
regulation of
biological
process;GO:0048
522//positive
regulation of
cellular
process;GO:0048
523//negative
regulation of
cellular
process;GO:0048
583//regulation
of response to
stimulus;GO:004
8699//generation
of
neurons;GO:004
8731//system
development;GO
:0048856//anato
mical structure
development;GO
:0048869//cellula
r developmental
process;GO:0050
767//regulation
of
neurogenesis;GO
:0050768//negati

ve regulation of
neurogenesis;GO
:0050770//regula
tion of
axonogenesis;G
O:0050771//nega
tive regulation of
axonogenesis;G
O:0050789//regu
lation of
biological
process;GO:0050
793//regulation
of developmental
process;GO:0050
794//regulation
of cellular
process;GO:0050
896//response to
stimulus;GO:005
1056//regulation
of small GTPase
mediated signal
transduction;GO:
0051093//negativ
e regulation of
developmental
process;GO:0051
128//regulation
of cellular
component
organization;GO:

0051129//negativ
e regulation of
cellular
component
organization;GO:
0051239//regulat
ion of
multicellular
organismal
process;GO:0051
241//negative
regulation of
multicellular
organismal
process;GO:0051
716//cellular
response to
stimulus;GO:005
1960//regulation
of nervous
system
development;GO
:0051961//negati
ve regulation of
nervous system
development;GO
:0060284//regula
tion of cell
development;GO
:0065007//biolog
ical
regulation;GO:00

														65009//regulation of molecular function;GO:0120035//regulation of plasma membrane bounded cell projection organization;GO:1902531//regulation of intracellular signal transduction;GO:2000026//regulation of multicellular organismal development	
ncbi_113145556	SLC35A2	26.69667	11.79467	12.211	12.934	12.68733	XP_026188194.1 UDP-galactose translocator isoform	-	-	-	-	GO:0000139//Golgi membrane;GO:0005575//cellular_component;GO:0005622//intracellular anatomical structure;GO:0005634//nucleus;GO:0005737//cytoplasm;GO:0005783//endoplasmic reticulum;GO:00	GO:0003674//molecular_function;GO:0005215//transporter activity;GO:0005338//nucleotide-sugar transmembrane transporter activity;GO:0005459//UDP-galactose transmembrane	GO:0005975//carbohydrate metabolic process;GO:0005996//monosaccharide metabolic process;GO:0006012//galactose metabolic process;GO:0006810//transport;GO:0006811//ion transport;GO:000	- -

2	X1 [Mastacembelus armatus]		05794//Golgi apparatus;GO:0005795//Golgi stack;GO:0005797//Golgi medial cisterna;GO:0012505//endomembrane system;GO:0016020//membrane;GO:0016021//integral component of membrane;GO:0031090//organelle membrane;GO:0031224//intrinsic component of membrane;GO:0031984//organelle subcompartment;GO:0031985//Golgi cisterna;GO:0043226//organelle;GO:0043227//membrane-bounded organelle;GO:0043229//intracellular	transporter activity;GO:0005463//UDP-N-acetylgalactosamine transmembrane transporter activity;GO:0015165//pyrimidine nucleotide-sugar transmembrane transporter activity;GO:0015932//nucleobase-containing compound transmembrane transporter activity;GO:0022857//transmembrane transporter activity;GO:1901505//carbohydrate derivative transmembrane transporter activity	6820//anion transport;GO:0008150//biological_process;GO:0008152//metabolic process;GO:0008643//carbohydrate transport;GO:0008645//hexose transmembrane transport;GO:0015711//organic anion transport;GO:0015749//monosaccharide transmembrane transport;GO:0015757//galactose transmembrane transport;GO:0015780//nucleotide-sugar transmembrane transport;GO:0015789//UDP-N-acetylgalactosamine transmembrane transport;GO:0015931//nucleobase

organelle;GO:0043231//intracellular membrane-bounded organelle;GO:0098588//bounding membrane of organelle;GO:0098791//Golgi apparatus subcompartment	-containing compound transport;GO:0019318//hexose metabolic process;GO:0034219//carbohydrate transmembrane transport;GO:0034220//ion transmembrane transport;GO:0044238//primary metabolic process;GO:0044281//small molecule metabolic process;GO:0051179//localization;GO:0051234//establishment of localization;GO:0055085//transmembrane transport;GO:0071702//organic substance transport;GO:0071704//organic substance metabolic

														process;GO:0071705//nitrogen compound transport;GO:0072334//UDP-galactose transmembrane transport;GO:0090481//pyrimidine nucleotide-sugar transmembrane transport;GO:0098656//anion transmembrane transport;GO:1901264//carbohydrate derivative transport	
ncbi_113146196	Jph2	4.220667	1.447667	1.316667	1.761333	1.403	XP_026189311.1 junctophilin-2 [Mastacembelus armatus]	-	-	-	-	GO:0005575//cellular_component;GO:0005622//intracellular anatomical structure;GO:0005737//cytoplasm;GO:0005783//endoplasmic reticulum;GO:0005789//endoplasmic reticulum membrane;GO:0005886//plasma	GO:0001786//phosphatidylserine binding;GO:0003674//molecular_function;GO:0005215//transporter activity;GO:0005216//ion channel activity;GO:0005217//intracellular ligand-gated ion channel activity;GO:0005261//cation	GO:0006810//transport;GO:0006811//ion transport;GO:0006812//cation transport;GO:0006816//calcium ion transport;GO:0006873//cellular ion homeostasis;GO:0006874//cellular calcium ion	- -

membrane;GO:0012505//endomembrane system;GO:0014701//junctional sarcoplasmic reticulum membrane;GO:0016020//membrane;GO:0016021//integral component of membrane;GO:0016528//sarcoplasm;GO:0016529//sarcoplasmic reticulum;GO:0030016//myofibril;GO:0030017//sarcomere;GO:0030018//Z disc;GO:0030314//junctional membrane complex;GO:0031090//organelle membrane;GO:0031224//intrinsic component of membrane;GO:0031674//I band;GO:003198	channel activity;GO:0005262//calcium channel activity;GO:0005488//binding;GO:0005543//phospholipid binding;GO:0005546//phosphatidylinositol-4,5-bisphosphate binding;GO:0005547//phosphatidylinositol-3,4,5-trisphosphate binding;GO:0008289//lipid binding;GO:0008324//cation transmembrane transporter activity;GO:0010314//phosphatidylinositol-5-phosphate binding;GO:0015075//ion transmembrane transporter activity;GO:0015085//calcium ion	homeostasis;GO:0006875//cellular metal ion homeostasis;GO:0007154//cell communication;GO:0007165//signal transduction;GO:0007204//positive regulation of cytosolic calcium ion concentration;GO:0007275//multicellular organism development;GO:0008150//biological_process;GO:0009966//regulation of signal transduction;GO:0009967//positive regulation of signal transduction;GO:0009987//cellular process;GO:0010522//regulation of calcium ion transport into

4//organelle subcompartment;GO:0032991//protein-containing complex;GO:0033017//sarcoplasmic reticulum membrane;GO:0042175//nuclear outer membrane-endoplasmic reticulum membrane network;GO:0043226//organelle;GO:0043227//membrane-bounded organelle;GO:0043228//non-membrane-bounded organelle;GO:0043229//intracellular organelle;GO:0043231//intracellular membrane-bounded organelle;GO:0043232//intracellular non-membrane-	transmembrane transporter activity;GO:0015267//channel activity;GO:0015276//ligand-gated ion channel activity;GO:0015278//calcium-release channel activity;GO:0015318//inorganic molecular entity transmembrane transporter activity;GO:0022803//passive transmembrane transporter activity;GO:0022834//ligand-gated channel activity;GO:0022836//gated channel activity;GO:0022839//ion gated channel activity;GO:0022857//transmembrane transporter activity;GO:0022	cytosol;GO:0010524//positive regulation of calcium ion transport into cytosol;GO:0010646//regulation of cell communication;GO:0010647//positive regulation of cell communication;GO:0010959//regulation of metal ion transport;GO:0016202//regulation of striated muscle tissue development;GO:0019722//calcium-mediated signaling;GO:0019725//cellular homeostasis;GO:0019932//second-messenger-mediated signaling;GO:0022898//regulation of

bounded organelle;GO:0043292//contractile fiber;GO:0071944//cell periphery;GO:0098588//bounding membrane of organelle;GO:0098827//endoplasmic reticulum subcompartment;GO:0099080//supramolecular complex;GO:0099081//supramolecular polymer;GO:0099512//supramolecular fiber	890//inorganic cation transmembrane transporter activity;GO:0032266//phosphatidylinositol-3-phosphate binding;GO:0035091//phosphatidylinositol binding;GO:0043167//ion binding;GO:0043168//anion binding;GO:0046873//metal ion transmembrane transporter activity;GO:0070273//phosphatidylinositol-4-phosphate binding;GO:0070300//phosphatidic acid binding;GO:0072341//modified amino acid binding;GO:0080025//phosphatidylinositol-3,5-	transmembrane transporter activity;GO:0023051//regulation of signaling;GO:0023052//signaling;GO:0023056//positive regulation of signaling;GO:0030001//metal ion transport;GO:0030003//cellular cation homeostasis;GO:0032409//regulation of transporter activity;GO:0032411//positive regulation of transporter activity;GO:0032412//regulation of ion transmembrane transporter activity;GO:0032414//positive regulation of ion transmembrane transporter

bisphosphate binding;GO:0099094//ligand-gated cation channel activity;GO:0099604//ligand-gated calcium channel activity;GO:1901981//phosphatidylinositol phosphate binding;GO:1902936//phosphatidylinositol bisphosphate binding	activity;GO:0032501//multicellular organismal process;GO:0032502//developmental process;GO:0032879//regulation of localization;GO:0034220//ion transmembrane transport;GO:0034762//regulation of transmembrane transport;GO:0034764//positive regulation of transmembrane transport;GO:0034765//regulation of ion transmembrane transport;GO:0034767//positive regulation of ion transmembrane transport;GO:0035556//intracellular signal transduction;GO:

0042592//homeo
static
process;GO:0043
269//regulation
of ion
transport;GO:004
3270//positive
regulation of ion
transport;GO:004
4093//positive
regulation of
molecular
function;GO:004
8518//positive
regulation of
biological
process;GO:0048
519//negative
regulation of
biological
process;GO:0048
522//positive
regulation of
cellular
process;GO:0048
523//negative
regulation of
cellular
process;GO:0048
583//regulation
of response to
stimulus;GO:004

8584//positive
regulation of
response to
stimulus;GO:004
8634//regulation
of muscle organ
development;GO
:0048856//anato
mical structure
development;GO
:0048878//chemi
cal
homeostasis;GO:
0050789//regulat
ion of biological
process;GO:0050
793//regulation
of developmental
process;GO:0050
794//regulation
of cellular
process;GO:0050
801//ion
homeostasis;GO:
0050848//regulat
ion of calcium-
mediated
signaling;GO:00
50850//positive
regulation of
calcium-
mediated

signaling;GO:00
50896//response
to
stimulus;GO:005
1049//regulation
of
transport;GO:005
1050//positive
regulation of
transport;GO:005
1179//localizatio
n;GO:0051209//r
elease of
sequestered
calcium ion into
cytosol;GO:0051
234//establishme
nt of
localization;GO:
0051239//regulat
ion of
multicellular
organismal
process;GO:0051
279//regulation
of release of
sequestered
calcium ion into
cytosol;GO:0051
281//positive
regulation of
release of

sequestered calcium ion into cytosol;GO:0051282//regulation of sequestering of calcium ion;GO:0051283//negative regulation of sequestering of calcium ion;GO:0051480//regulation of cytosolic calcium ion concentration;GO:0051641//cellular localization;GO:0051649//establishment of localization in cell;GO:0051716//cellular response to stimulus;GO:0051924//regulation of calcium ion transport;GO:0051928//positive regulation of calcium ion

transport;GO:005
5024//regulation
of cardiac muscle
tissue
development;GO
:0055065//metal
ion
homeostasis;GO:
0055074//calciu
m ion
homeostasis;GO:
0055080//cation
homeostasis;GO:
0055082//cellular
chemical
homeostasis;GO:
0055085//transm
embrane
transport;GO:006
0314//regulation
of ryanodine-
sensitive
calcium-release
channel
activity;GO:0060
316//positive
regulation of
ryanodine-
sensitive
calcium-release
channel
activity;GO:0060

341//regulation of cellular localization;GO:0060401//cytosolic calcium ion transport;GO:0060402//calcium ion transport into cytosol;GO:0065007//biological regulation;GO:0065008//regulation of biological quality;GO:0065009//regulation of molecular function;GO:0070588//calcium ion transmembrane transport;GO:0072503//cellular divalent inorganic cation homeostasis;GO:0072507//divalent inorganic cation homeostasis;GO:0097553//calcium ion transmembrane import into

cytosol;GO:0098
655//cation
transmembrane
transport;GO:009
8660//inorganic
ion
transmembrane
transport;GO:009
8662//inorganic
cation
transmembrane
transport;GO:009
8771//inorganic
ion
homeostasis;GO:
1901019//regulat
ion of calcium
ion
transmembrane
transporter
activity;GO:1901
021//positive
regulation of
calcium ion
transmembrane
transporter
activity;GO:1901
861//regulation
of muscle tissue
development;GO
:1902531//regula
tion of

intracellular
signal
transduction;GO:
1902533//positiv
e regulation of
intracellular
signal
transduction;GO:
1903169//regulat
ion of calcium
ion
transmembrane
transport;GO:190
4062//regulation
of cation
transmembrane
transport;GO:190
4064//positive
regulation of
cation
transmembrane
transport;GO:190
4427//positive
regulation of
calcium ion
transmembrane
transport;GO:200
0026//regulation
of multicellular
organismal
development;GO
:2001257//regula

tion of cation
channel
activity;GO:2001
259//positive
regulation of
cation channel
activity

Transcriptomics testing

1 RNA Extraction, library construction and sequencing

Total RNA was extracted using Trizol reagent kit (Invitrogen, Carlsbad, CA, USA) according to the manufacturer's protocol. RNA quality was assessed on an Agilent 2100 Bioanalyzer (Agilent Technologies, Palo Alto, CA, USA) and checked using RNase free agarose gel electrophoresis. After total RNA was extracted, eukaryotic mRNA was enriched by Oligo (dT) beads. Then the enriched mRNA was fragmented into short fragments using fragmentation buffer and reversely transcribed into cDNA by using NEBNext Ultra RNA Library Prep Kit for Illumina (NEB #7530, New England Biolabs, Ipswich, MA, USA). The purified double-stranded cDNA fragments were end repaired, A base added, and ligated to Illumina sequencing adapters. The ligation reaction was purified with the AMPure XP Beads (1.0X). And polymerase chain reaction (PCR) amplified. The resulting cDNA library was sequenced using Illumina Novaseq6000 by Gene Denovo Biotechnology Co. (Guangzhou, China).

2 Bioinformatics analysis

Filtering of Clean Reads

Reads obtained from the sequencing machines includes raw reads containing adapters or low-quality bases which will affect the following assembly and analysis. Thus, to get high quality clean reads, reads were further filtered by fastp (version 0.18.0) [1]. The parameters were as follows:

removing reads containing adapters;

removing reads containing more than 10% of unknown nucleotides(N);

removing low quality reads containing more than 50% of low quality (Q-value≤20) bases.

Alignment with Ribosome RNA (rRNA)

Short reads alignment tool Bowtie2 (version 2.2.8) was used for mapping reads to ribosome RNA (rRNA) database [2]. The rRNA mapped reads then will be removed. The remaining clean reads were further used in assembly and gene abundance calculation.

Alignment with Reference Genome

An index of the reference genome was built, and paired-end clean reads were mapped to the reference genome using HISAT2. 2.4 and other parameters set as a default [3].

Quantification of Gene Abundance

The mapped reads of each sample were assembled by using StringTie v1.3.1 in a reference-based approach [4, 5]. For each transcription region, a FPKM (fragment per kilobase of transcript per million mapped reads) value was calculated to quantify its expression abundance and variations, using RSEM software [6]. The FPKM method can eliminate the influence of different gene lengths and sequencing data amount on the calculation of gene expression. Therefore, the calculated gene expression can be directly used for comparing the difference of gene expression among samples.

The mapped reads of each sample were assembled by using StringTie v1.3.1 in a reference-based approach. For each transcription region, a TPM (Transcripts Per Kilobase of exon model per Million mapped reads) value was calculated to quantify its expression abundance and variations, using RSEM software [4-6]. The TPM method can eliminate the influence of different gene lengths and sequencing data amount on the calculation of gene expression. Therefore, the calculated gene expression can be directly used for comparing the difference of gene expression among samples.

3 Relationship analysis of samples

Correlation Analysis of Replicas

Correlation analysis was performed by R. Correlation of two parallel experiments provides the evaluation of the reliability of experimental results as well as operational stability. The correlation coefficient between two replicas was calculated to evaluate repeatability between samples. The closer the correlation

coefficient gets to 1, the better the repeatability between two parallel experiments.

Principal Component Analysis

Principal component analysis (PCA) was performed with R package gmodels (http://www.r-project.org/) in this experience. PCA is a statistical procedure that converts hundreds of thousands of correlated variables (gene expression) into a set of values of linearly uncorrelated variables called principal components. PCA is largely used to reveal the structure/relationship of the samples/dates.

Differentially expressed genes (DEGs)

RNAs differential expression analysis was performed by DESeq2 software between two different groups (and by edgeR between two samples) [7, 8]. The genes/transcripts with the parameter of false discovery rate (FDR) below 0.05 and absolute fold change≥2 was considered differentially expressed genes/transcripts.

GO Enrichment Analysis

Gene Ontology (GO) is an international standardized gene functional classification system which offers a dynamic-updated controlled vocabulary and a strictly defined concept to comprehensively describe properties of genes and their products in any organism [9]. GO has three ontologies: molecular function, cellular component, and biological process. The basic unit of GO is GO-term. Each GO-term belongs to a type of ontology.

GO enrichment analysis provides all GO terms that significantly enriched in DEGs comparing to the genome background, and filter the DEGs that correspond to biological functions. Firstly, all DEGs were mapped to GO terms in the Gene Ontology database (http://www.geneontology.org/), gene numbers were calculated for every term, significantly enriched GO terms in DEGs comparing to the genome background were defined by hypergeometric test. The calculated p-value were gone through FDR Correction, taking FDR ≤ 0.05 as a threshold.

GO terms meeting this condition were defined as significantly enriched GO terms in DEGs. This analysis was able to recognize the main biological functions that DEGs exercise.

Pathway Enrichment Analysis

Genes usually interact with each other to play roles in certain biological functions. Pathway-based analysis helps to further understand genes biological functions. KEGG is the major public pathway-related database [10]. Pathway enrichment analysis identified significantly enriched metabolic pathways or signal transduction pathways in DEGs comparing with the whole genome background. The calculated p-value was gone through FDR Correction, taking FDR ≤ 0.05 as a threshold. Pathways meeting this condition were defined as significantly enriched pathways in DEGs

Disease Ontology Enrichment Analysis (only apply in human genome reference)

The Disease Ontology (DO) has been developed as a standardized ontology for human disease with the purpose of providing the biomedical community with consistent, reusable, and sustainable descriptions of human disease terms, phenotype characteristics and related medical vocabulary disease concepts [11]. DO enrichment analysis identified significantly enriched human disease DO terms in DEGs comparing with the whole genome background.

The calculated p-value was gone through FDR Correction, taking FDR ≤ 0.05 as a threshold. Do terms meeting this condition were defined as significantly enriched Doterms in DEGs

Reactome Enrichment Analysis (only apply in human genome reference)

The Reactome is a free online database of biological pathways. The core unit of the Reactome data model is the reaction. Entities (nucleic acids, proteins, complexes, and small molecules) participating in reactions form a network of biological interactions and are grouped into pathways [12, 13]. Examples of biological pathways in Reactome include signaling, innate and acquired immune function, transcriptional regulation, translation, apoptosis, and classical intermediary metabolism.

Reactome enrichment analysis identified significantly enriched reactions in DEGs comparing with the whole genome background. The calculated p-value was gone through FDR Correction, taking FDR ≤ 0.05 as a threshold. Reactions meeting this condition were defined as significantly enriched Reactions in DEGs

Reference

[1] Chen S, Zhou Y, Chen Y et al. fastp: an ultra-fast all-in-one FASTQ preprocessor.[J]. Bioinformatics (Oxford, England), 2018,34(17):i884-i890.

[2] Langmead B, Salzberg S L. Fast gapped-read alignment with Bowtie 2.[J]. NAT METHODS, 2012,9(4):357-359.

[3] Kim D, Langmead B, Salzberg S L. HISAT: a fast spliced aligner with low memory requirements.[J]. NAT METHODS, 2015,12(4):357-360.

[4] Pertea M, Pertea G M, Antonescu C M et al. StringTie enables improved reconstruction of a transcriptome from RNA-seq reads.[J]. NAT BIOTECHNOL, 2015,33(3):290-295.

[5] Pertea M, Kim D, Pertea G M et al. Transcript-level expression analysis of RNA-seq experiments with HISAT, StringTie and Ballgown.[J]. NAT PROTOC, 2016,11(9):1650-1667.

[6] Li B, Dewey C N. RSEM: accurate transcript quantification from RNA-Seq data with or without a reference genome.[J]. BMC BIOINFORMATICS, 2011,12:323.

[7] Love M I, Huber W, Anders S. Moderated estimation of fold change and dispersion for RNA-seq data with DESeq2.[J]. GENOME BIOL, 2014,15(12):550.

[8] Robinson M D, McCarthy D J, Smyth G K. edgeR: a Bioconductor package for differential expression analysis of digital gene expression data.[J]. Bioinformatics (Oxford, England), 2010,26(1):139-140.

[9] Ashburner M, Ball C A, Blake J A et al. Gene ontology: tool for the unification of biology. The Gene Ontology Consortium.[J]. NAT GENET, 2000,25(1):25-29.

[10] Kanehisa M, Goto S. KEGG: kyoto encyclopedia of genes and genomes.[J]. NUCLEIC ACIDS RES, 2000,28(1):27-30.

[11] Schriml L M, Arze C, Nadendla S et al. Disease Ontology: a backbone for disease semantic integration.[J]. NUCLEIC ACIDS RES, 2012,40(Database issue):D940-D946.

[12] Croft D, O'Kelly G, Wu G et al. Reactome: a database of reactions, pathways and biological processes.[J]. NUCLEIC ACIDS RES, 2011,39(Database issue):D691-D697.

[13] Jassal B, Matthews L, Viteri G et al. The reactome pathway knowledgebase.[J]. NUCLEIC ACIDS RES, 2020,48(D1):D498-D503.

Experimental Methods

1. Chemicals

Ammonium acetate (NH4AC) was purchased from Sigma Aldrich, Acetonitrile was purchased from Merck, ammonium hydroxide (NH4OH) and methanol were purchased from Fisher.

2. Sample Collection and Preparation

Animal Tissues

The animal tissues (e.g., mice liver) were quickly frozen in liquid nitrogen immediately after dissection. Then the tissue were cut on dry ice (~80 mg) into an Eppendorf tube (2 mL). The tissue samples with 200 μL of H2O and five ceramic beads were homogenized using the homogenizer. 800 μL methanol/acetonitrile (1:1, v/v) were added to homogenized solution for metabolite extraction.

The mixture was centrifuged for 15 min (14000 g, 4 °C). The supernatant was dried in a vacuum centrifuge. For LC-MS analysis, the samples were re-dissolved in 100 μL acetonitrile/water (1:1, v/v) solvent.

To monitor the stability and repeatability of instrument analysis, quality control (QC) samples were prepared by pooling 10 μL of each sample and analyzed together with the other samples. The QC samples were inserted regularly and analyzed in every 5 samples.

3. LC-MS/MS Analysis

Analysis was performed using an UHPLC (1290 Infinity LC, Agilent Technologies) coupled to a quadrupole time-of-flight (AB Sciex TripleTOF 6600) in Shanghai Applied Protein Technology Co., Ltd.

For HILIC separation, samples were analyzed using a 2.1 mm × 100 mm ACQUIY UPLC BEH 1.7 µm column (waters, Ireland). In both ESI positive and negative modes, the mobile phase contained A=25 mM ammonium acetate and 25 mM ammonium hydroxide in water and B= acetonitrile. The gradient was 85% B for 1 min and was linearly reduced to 65% in 11 min, and then was reduced to 40% in 0.1 min and kept for 4 min, and then increased to 85% in 0.1 min, with a 5 min re-equilibration period employed.

For RPLC separation, a 2.1 mm × 100 mm ACQUIY UPLC HSS T3 1.8 µm column (Waters,

Ireland) was used. In ESI positive mode, the mobile phase contained A= water with

0.1% formic acid and B= acetonitrile with 0.1% formic acid; and in ESI negative mode, the mobile phase contained A=0.5 mM ammonium fluoride in water and B= acetonitrile. The gradient was 1%B for 1.5 min and was linearly increased to 99% in 11.5 min and kept for 3.5 min. Then it was reduced to 1% in 0.1 min and a

3.4 min of re-equilibration period was employed. The gradients were at a flow rate of 0.3mL/min, and the column temperatures were kept constant at 25 ℃. A 2 μL aliquot of each sample was injected.

The ESI source conditions were set as follows: Ion Source Gas1 (Gas1) as 60, Ion Source Gas2 (Gas2) as 60, curtain gas (CUR) as 30, source temperature: 600 ℃, IonSpray Voltage Floating (ISVF) ± 5500 V. In MS only acquisition, the instrument was set to acquire over the m/z range 60-1000 Da, and the accumulation time for TOF MS scan was set at 0.20 s/spectra. In auto MS/MS acquisition, the instrument was set to acquire over the m/z range 25-1000 Da, and the accumulation time for product ion scan was set at 0.05 s/spectra. The product ion scan is acquired using information dependent acquisition (IDA) with high sensitivity mode selected. The parameters were set as follows: the collision energy (CE) was fixed at 35 V with ± 15 eV; declustering potential (DP), 60 V (+) and−60 V (−); exclude isotopes within 4 Da, candidate ions to monitor per cycle: 10.

4. Data processing

The raw MS data were converted to MzXML files using ProteoWizard MSConvert before importing into freely available XCMS software. For peak picking, the following parameters were used: centWave m/z = 10 ppm, peakwidth = c (10, 60), prefilter = c (10, 100). For peak grouping, bw = 5, mzwid = 0.025, minfrac = 0.5 were used. CAMERA (Collection of Algorithms of MEtabolite pRofile Annotation) was sued for annotation of isotopes and adducts. In the extracted ion features, only the variables having more than 50% of the nonzero measurement values in at least one group were kept. Compound identification of metabolites was performed by comparing of accuracy m/z value (<10 ppm), and MS/MS spectra with an in-house database established with available authentic standards.The missing data were filled by KNN (K-Nearest Neighbor) method, and the extreme values were deleted. Finally, the total peak area of the data was normalized to ensure the parallelism between samples and metabolites.

5. Data quality control

Positive ion mode (POS) and negative ion mode (NEG) are both used to detect metabolites, which can make the metabolite coverage higher and the detection effect better. In the process of subsequent data analysis, the positive and negative ion models are analyzed separately. QC samples are usually used for quality control when the study of metabolomics is based on mass spectrometry (Dunn et al., 2011). Theoretically, QC samples are the same. But there will be systematic errors in the process of sample extraction, detection or analysis, which will lead to differences between QC samples. Principle component analysis (PCA) was performed by R language gmodels (v2.18.1) (Available online: https://CRAN.R-project.org/package=gmodels). The denser the distribution of QC is the more reliable results is.

6. Cluster analysis

6.1 Hierarchical cluster analysis

A dendrogram is a diagram that shows the hierarchical relationship between objects. Hierarchical cluster analysis (HCA) was performed on the data and the resulting dendrogram was calculated using the average linkage method (Yuan et al., 2012).

6.2 Cluster heatmap analysis

Data were normalized by z-score and hierarchical clustered by R package pheatmap. The heatmap can display the expression patterns of metabolites in all samples.

6.3 Correlation analysis of replicas

Variability of metabolite composition and abundance between samples can be quantified by correlation data between samples. Correlation analysis was performed by R and drawing heatmap by R package pheatmap. The closer the correlation coefficient gets to 1, the higher the similarity of metabolic composition and abundance are between samples.

7. Multivariate statistical analysis

7.1 PCA analysis

For a preliminary visualization of differences between different groups of samples, the unsupervised dimensionality reduction method principal component analysis (PCA)

was applied in all samples using R package models (http://www.r-project.org/) (Yuan et al., 2012). PCA is a statistical procedure that converts hundreds of thousands of correlated metabolites variables into a set of values of linearly uncorrelated variables called principal components.

7.2 PLS-DA

Partial least squares discriminant analysis (PLS-DA) is a supervised dimensionality reduction method in which class memberships are coded in matrix form into Y to better distinguish the metabolomics profile of two groups by screening variables correlated to class memberships (Worley et al., 2013). PLS- DA was applied in comparison groups using R package ropls (http://www.r-project.org/).

7.3 OPLS-DA

Orthogonal projection to latent structures-discriminant analysis (OPLS-DA) is an extension of PLS-DA which incorporates an Orthogonal Signal Correction (OSC) filter into a PLS model (Bylesjo et al., 2006). The basic concept in OPLS is to separate the systematic variation in X into two parts, one that is correlated to Y and one that is not correlated (orthogonal) with Y. Only the Y- predictive variation is used to model the data. OPLS-DA was applied in comparison groups using R package models (http://www.r-project.org/).

The OPLS-DA model was further validated by cross-validation and permutation test (Saccenti et al., 2014). For cross-validation, the data was partitioned into seven subsets, where each of the subsets was then used as a validation set. R2 indicated the total variation in the data matrix that was explained by the model. Predictive ability (Q2) values represented the most recognized diagnostic statistical parameter to validate the OPLS-DA model in metabolomics.

Acceptable predictive model is considered for Q2 value greater than 0.4. Good predictive model is considered for Q2 value greater than 0.9. Permutation test randomly permutes class labels for 200 times and then produces a distribution of R2’ values and Q2’ values. In essence, a reliable model should yield significantly larger R2 and Q2 value compared to R2’ and Q2’ values generated from random models using the same data set.

8. Differential metabolites analysis

8.1 Differential metabolites identify

A variable importance in projection (VIP) score of (O)PLS model was applied to

rank the metabolites that best distinguished between two groups. The threshold of VIP was set to 1. In addition, T-test was also used as a univariate analysis for screening differential metabolites. Those with a p value of T test$<$0.05 and VIP$\geq$1 were considered differential metabolites between two groups.

8.2 Differential metabolites volcano analysis

To understand the regulation of differential metabolites, the fold changes of abundance between two groups were further calculated to draw the volcano plot.

8.3 Differential metabolites VIP statistics

The abundance of differential metabolites in the same group was normalized by z-score. And then, the VIP score of OPLS-DA was used to draw the graph. The top 15 metabolites are shown in the variable importance in projection (VIP) score plot in descending order (Yoon et al., 2020).

8.4 Differential metabolites correlation heatmap

Pearson correlation coefficient and p-value were calculated by R function, cor. And cor.test. The closer the correlation coefficient gets to 1, the higher the similarity of metabolic abundance are. Correlations were called significant when $p \leq 0.05$ (Rao et al., 2016). And correlation heatmap was drawn by R corrplot package.

8.5 Differential metabolites cluster heatmap

The abundances of differential metabolites were normalized by z-score and hierarchical clustered by R package pheatmap to show the accumulation differences between two groups.

8.6 Differential metabolites z-score analysis

Z-score (standard fraction) is based on the relative content conversion of metabolites, which is used to measure the relative content of metabolites at the same level. The calculating formula is as follows:

$z = (x – \mu) / \sigma$.

Here x is the abundance of metabolite, μ is the mean of the abundance of metabolite in every sample, σ is the standard deviation (Chen et al., 2015).

8.7 Differential metabolites ROC analysis

ROC (Receiver Operating Characteristic) curve analysis by R pROC package to evaluate the predictive power of each of the discriminant metabolites. The cutpoint was

determined for each biomarker by searching for those that yielded both high sensitivity and specificity. ROC curves were then plotted based on the set of optimal sensitivity and specificity values. Area under the curve (AUC) was computed via numerical integration of the ROC curves. The metabolite signature that has the largest area under the ROC curve was identified as having the strongest predictive power for discrimating two groups (Wei et al., 2011). The ROC curve was analyzed using R package pROC (Robin et al., 2011)according to the significant different metabolites between two groups.

9. Pathway analysis

9.1 KEGG annotations and enrichment analysis of differential expressed metabolites

Kyoto Encyclopedia of Genes and Genomes (KEGG) (Kanehisa et al., 2000) is the major public pathway-related database that includes not only genes but metabolites. Metabolites were mapped to KEGG metabolic pathways for annotation and enrichment analysis. Pathway enrichment analysis identified significantly enriched metabolic pathways or signal transduction pathways in differential metabolites comparing with the whole background. The calculating formula is as follows: Here N is the number of all metabolites that with KEGG annotation, n is the number of differential metabolites in N, M is the number of all metabolites annotated to specific pathways, and m is number of differential metabolites in M. The calculated p-value was gone through FDR Correction, taking FDR$\leqslant$0.05 as a threshold. Pathways meeting this condition were defined as significantly enriched pathways in differential metabolites.

9.2 MSEA

Metabolic Set Enrichment Analysis (MSEA) (Xia et al., 2010) was also used to evaluate for pathway over-representation using the MetaboAnalyst module. The Small Molecule Pathway Database (SMPDB) library was used for the analysis, and global test for the quantitative enrichment analysis(QEA) by R package MSEAp

10. Trend analysis

Metabolites expression pattern analysis is used to cluster metabolites of similar expression patterns for multiple samples (at least 3 in a specific time point, space, or

treatment dose size order). To examine the expression pattern of all annotated metabolites, the expression data of each sample (in the order of treatment) were normalized to 0, log2 (v1/v0), log2(v2/v0), and then clustered by Short Time-series Expression Miner software (STEM, version 1.3.11). The parameters were set as follows:

1) Maximum Unit Change in Model Profiles between Time Points is 1;

2) Maximum output profiles number is 20 (similar profiles will be merged).

The clustered profiles with p-value ≤0.05 were considered as significant profiles. Then the metabolites in all or each profile were subjected to KEGG pathway enrichment analysis. Through the hypothesis test of the p-value calculation and FDR (Benjamini et al., 1995) correction, the Pathways with Qvalue≤0.05 were defined as significant enriched pathways.

References

1. Chen, S.; Zhou, Y.; Chen, Y.; Gu, J. fastp: an ultra-fast all-in-one FASTQ preprocessor. *Bioinformatics (Oxford, England)* **2018**, 34, i884-i890.
2. Langmead, B.; Salzberg, S.L. Fast gapped-read alignment with Bowtie 2. *Nat Methods* **2012**, 9, 357-9.
3. Kim, D.; Langmead, B.; Salzberg, S.L. HISAT: a fast spliced aligner with low memory requirements. *Nat Methods* **2015**, 12, 357-60.
4. Pertea, M.; Pertea, G.M.; Antonescu, C.M.; Chang, T.; Mendell, J.T.; Salzberg, S.L. StringTie enables improved reconstruction of a transcriptome from RNA-seq reads. *Nat Biotechnol* **2015**, 33, 290-5.
5. Pertea, M.; Kim, D.; Pertea, G.M.; Leek, J.T.; Salzberg, S.L. Transcript-level expression analysis of RNA-seq experiments with HISAT, StringTie and Ballgown. *Nat Protoc* **2016**, 11, 1650-67.
6. Li, B.; Dewey, C.N. RSEM: accurate transcript quantification from RNA-Seq data with or without a reference genome. *Bmc Bioinformatics* **2011**, 12, 323.

7. Love, M.I.; Huber, W.; Anders, S. Moderated estimation of fold change and dispersion for RNA-seq data with DESeq2. *Genome Biol* **2014**, 15, 550.
8. Robinson, M.D.; McCarthy, D.J.; Smyth, G.K. edgeR: a Bioconductor package for differential expression analysis of digital gene expression data. *Bioinformatics (Oxford, England)* **2010**, 26, 139-40.
9. Ashburner, M.; Ball, C.A.; Blake, J.A.; Botstein, D.; Butler, H.; Cherry, J.M.; Davis, A.P.; Dolinski, K.; Dwight, S.S.; Eppig, J.T.; et al. Gene ontology: tool for the unification of biology. The Gene Ontology Consortium. *Nat Genet* **2000**, 25, 25-9.
10. Kanehisa, M.; Goto, S. KEGG: kyoto encyclopedia of genes and genomes. *Nucleic Acids Res* **2000**, 28, 27-30.
11. Schriml, L.M.; Arze, C.; Nadendla, S.; Chang, Y.W.; Mazaitis, M.; Felix, V.; Feng, G.; Kibbe, W.A. Disease Ontology: a backbone for disease semantic integration. *Nucleic Acids Res* **2012**, 40, D940-6.
12. Croft, D.; O'Kelly, G.; Wu, G.; Haw, R.; Gillespie, M.; Matthews, L.; Caudy, M.; Garapati, P.; Gopinath, G.; Jassal, B.; et al. Reactome: a database of reactions, pathways and biological processes. *Nucleic Acids Res* **2011**, 39, D691-7.
13. Jassal, B.; Matthews, L.; Viteri, G.; Gong, C.; Lorente, P.; Fabregat, A.; Sidiropoulos, K.; Cook, J.; Gillespie, M.; Haw, R.; et al. The reactome pathway knowledgebase. *Nucleic Acids Res* **2020**, 48, D498-D503.
